# Supplementary material for: Prevalence and correlates of depressive symptoms among gay, bisexual and other men who have sex with men in the PROUD randomised clinical trial of HIV pre-exposure prophylaxis
Source: BMJ Open. 2019 Dec 10;9(12):e031085. doi: 10.1136/bmjopen-2019-031085 (PMC6924847; doi:10.1136/bmjopen-2019-031085)
Supplement: Author's manuscript [file bmjopen-2019-031085.draft_revisions.pdf]

BMJ Open

BMJ Open is committed to open peer review. As part of this commitment we make the peer review history of every article we publish publicly available.

When an article is published we post the peer reviewers' comments and the authors' responses online. We also post the versions of the paper that were used during peer review. These are the versions that the peer review comments apply to.

The versions of the paper that follow are the versions that were submitted during the peer review process. They are not the versions of record or the final published versions. They should not be cited or distributed as the published version of this manuscript.

BMJ Open is an open access journal and the full, final, typeset and author-corrected version of record of the manuscript is available on our site with no access controls, subscription charges or pay-per-view fees (http://bmjopen.bmj.com).

If you have any questions on BMJ Open's open peer review process please email info.bmjopen@bmj.com

BMJ Open

Prevalence and correlates of depressive symptoms among gay, bisexual and other men who have sex with men in the PROUD trial of HIV pre-exposure prophylaxis

Journal:	*BMJ Open*
Manuscript ID	bmjopen-2019-031085
Article Type:	Research
Date Submitted by the Author:	15-Apr-2019

Complete List of Authors:	Miltz, Ada; University College London<br>Lampe, F; University College London<br>McCormack, Sheena; University College London, MRC CTU<br>Dunn, David; University College London, MRC CTU<br>White, Ellen; University College London, MRC CTU<br>Rodger, Alison; University College London<br>Phillips, Andrew; University College London<br>Sherr, Lorraine; University College London<br>Sullivan, Ann ; Chelsea and Westminster Healthcare NHS Trust<br>Reeves, Iain; Homerton University Hospital NHS Trust, Sexual Health<br>Clarke, Amanda; Elton John Centre, Sussex House<br>Gafos, Mitzy; London School of Hygiene and Tropical Medicine

Keywords:	MENTAL HEALTH, EPIDEMIOLOGY, GENITOURINARY MEDICINE, PUBLIC HEALTH

SCHOLARONE™
Manuscripts

Prevalence and correlates of depressive symptoms among gay, bisexual and other men who have sex with men in the PROUD trial of HIV pre-exposure prophylaxis

Ada Miltz[1*], Fiona C Lampe[1], Sheena McCormack[2], David Dunn[2], Ellen White[2], Alison Rodger[1], Andrew Phillips[1], Lorraine Sherr[1], Ann K Sullivan[3], Iain Reeves[4], Amanda Clarke[5], Mitzy Gafos[6]

*Corresponding author, email: Ada.Miltz.11@ucl.ac.uk, telephone number: +44 20801 68058

Author affiliations

[1]Institute for Global Health, University College London, London, UK

[2] MRC Clinical Trials Unit, University College London, London, UK

[3] Chelsea and Westminster Hospital NHS Trust, London, UK

[4] Homerton Hospital NHS Trust, London, UK

[5] Elton John Centre, Sussex House, Brighton, UK

[6] Department of Global Health and Development, London School of Hygiene and Tropical Medicine, London, UK

For peer review only

Abstract

Objectives: The aim of this analysis is to: (i) present the prevalence of clinically significant depressive symptoms at baseline and follow-up of participants in the PROUD clinical trial of HIV pre-exposure prophylaxis (PrEP), examining changes in prevalence over time and (ii) investigate the association of socio-demographic, lifestyle, and psychosocial factors with depressive symptoms.

Methods: PROUD was an open label randomised trial evaluating the benefit of PrEP for 544 HIV-negative gay, bisexual, and other men who have sex with men (GBMSM) in England (enrolment 2012-2014; follow-up at quarterly visits until study end in October 2016). Modified Poisson regression and generalized estimating equations (GEEs) were used to examine cross-sectional associations of socio-demographic and lifestyle factors with depressive symptoms at baseline, and of psychosocial measures with depressive symptoms using the combined 12- and 24-month follow-up data. Prevalence ratios were presented unadjusted and adjusted (aPR) for age, born in the UK, sexual identity, university education, London study clinic site, calendar time (and follow-up time-point).

Results: Depressive symptoms (PHQ-9 score of ≥10) increased significantly from baseline (9.1%; 49/540) to the 12-month (14.4%; 59/410) and 24-month (14.4%; 48/333) follow-up visits, possibly explained by the suggestion of underreporting at baseline. The prevalence of depressive symptoms did not differ by study trial arm, at any time-point. In the analysis of baseline data, younger men, those who were not employed, and those who reported recent crystal methamphetamine use, were more likely to report depressive symptoms. In combined analysis of 12- and 24-month data, measures of intimate partner violence (IPV) (aPR, with lifetime IPV victimization 2.57 [95% CI: 1.71, 3.86]), internalized homophobia (aPR 1.91 [95% CI: 1.29, 2.83]), and concealment of sexual identity (aPR 1.75 [95% CI: 1.16, 2.65]), were strongly associated with depressive symptoms.

Conclusions: There is a high concomitant burden of psychosocial factors with depression among GBMSM.

Trial registration: ISRCTN (ISRCTN94465371) and ClinicalTrials.gov (NCT02065986).

For peer review only - http://bmjopen.bmj.com/site/about/guidelines.xhtml

Strengths and limitations of this study

- This is the first UK study to collect longitudinal data on depression prevalence over time among gay, bisexual and other men who have sex with men (GBMSM).
- This is the first UK study to investigate associations between a range of psychosocial factors and depression among GBMSM.
- Although chemsex drug use was investigated in this study (use of mephedrone, GHB/GBL, and/or methamphetamine), chemsex was not explicitly measured, as participants were not asked whether drug use took place in a sexual context.
- Since the data were analysed cross-sectionally, the direction of associations may be unclear for some factors, such as drug use or IPV.
- Given that the level of income/financial stability was not measured in this study, there is a possibility of unmeasured confounding.

Introduction

Depressive symptomatology encompasses feelings of worthlessness and hopelessness, anhedonia, sleep and appetite disturbance, and suicidal thoughts, which may be rooted in traumatic events (perhaps early on in life), and may reflect impaired neural functioning [1]. Depression can lead to loss of life through suicide, breakdown of family structures, and loss of workplace productivity [2]. Depression may also lower a sense of self-efficacy, cultivating the perception of being incapable of influencing events and social conditions that significantly impact on one's life, with negative implications for self-care and health seeking behaviours [3]. There is some evidence to suggest that those who experience depressive symptoms may be more likely to engage in escapism as a coping strategy, which may be linked to increased drug and alcohol use and sexual risk behaviour associated with STI/HIV transmission [4]. Maladaptive coping mechanisms in the context of depression may prevent individuals from recognizing and avoiding abusive relationships with an intimate partner [5].

Symptoms of depression have been found to be associated with increased sexual risk behaviour [6 7] in studies of gay, bisexual and other men who have sex with men (GBMSM), mainly in the U.S. It has been suggested in syndemic theory that it is the synergistic interaction of co-occurring psychosocial health problems, childhood sexual abuse, drug use, intimate partner violence (IPV), and depression that compound the risk of HIV infection among GBMSM [8]. In recent years there has been a visible shift in the UK research agenda to address mental health & wellbeing among the general population and sexual minorities in particular, for whom it is being increasingly recognized that the burden of

For peer review only - http://bmjopen.bmj.com/site/about/guidelines.xhtml

psychological distress may be higher [9]. As a result, a number of UK studies have examined the prevalence of, and factors associated with, depressive symptoms among GBMSM [10-15]. In half of these studies however, the sample size was too small to allow for a meaningful analysis (<200 GBMSM) of associated factors [10 12 13], and one limitation of the larger studies is that data were not collected on key psychosocial measures such as IPV and internalized homophobia [11 14 15], which may be linked to depression. There is a need to better understand correlates of depression among GBMSM in the UK.

This analysis uses data from the PROUD randomised clinical trial, which evaluated the impact of pre-exposure prophylaxis (PrEP) on HIV incidence, and collected data on a range of psychosocial factors. The aim is to: (i) present the prevalence of depressive symptoms at baseline and follow-up, examining changes in prevalence over time and (ii) investigate the association of socio-demographic, lifestyle, and psychosocial factors with depressive symptoms. The link between psychosocial factors, including depressive symptoms, and sexual risk behaviour among GBMSM in PROUD has been addressed in a separate paper.

Methods

The PROUD trial was a multi-centre, pragmatic open label randomised trial evaluating the benefit of PrEP as part of a package of HIV risk reduction interventions for HIV-negative GBMSM and trans women [16]. Only three trans women enrolled in PROUD, and therefore data cannot be presented separately for trans women. Participants were enrolled from routine attenders at 13 genitourinary medicine (GUM) clinics in England between November 2012 and April 2014. Volunteers were eligible if they met the following criteria: male at birth; aged 18 years or over; HIV-negative on the day of enrolment or in the past four weeks; reported condomless anal sex (CAS) in the past three months; and expected to have CAS again in the next three months [16]. Volunteers were randomised 1:1 to an immediate start of daily oral PrEP or a deferred start (after one year). Participants had the opportunity to remain in follow-up for at least two years. The study was reviewed and approved by London Bridge Research Ethics Committee (12/LO/1289).

Patient and Public Involvement

In line with the MRC CTU Patient and Public Involvement policy and the UNAIDS/AVAC Good Participatory Practice Guidelines, the PROUD pilot study team used a number of mechanisms to

For peer review only - http://bmjopen.bmj.com/site/about/guidelines.xhtml

promote effective and appropriate engagement in the research project (http://www.proud.mrc.ac.uk/about/patient-and-public-involvement-ppi/).

Clinically significant depressive symptoms

The Patient Health Questionnaire (PHQ-9) was used to measure the prevalence of depressive symptoms at baseline and on an approximately annual basis (month 12 and 24). In the PHQ-9, participants are asked to rate how often over the past two weeks they have experienced nine specific problems [17]. A depressive symptom score is generated by summing responses for each question, ranging from 'not at all' (coded as 0) to 'nearly every day' (coded as 3). In this paper, a missing response was coded as 0 as there appeared to be a common response pattern in which only those experiences that had occurred were ticked on the questionnaire. A cut-off point of 10 or greater indicates a possible clinically significant depressive condition [17].

The PROUD study team reported that part way through the trial enrolment process a change in PHQ-9 reporting policy was instated, which continued to be utilized throughout follow-up. From April 2013, participants were informed that after self-completing the questionnaire they would be asked to disclose their response to the suicidal/self-harm ideation question on the PHQ-9 ('Thoughts that you would be better off dead, or of hurting yourself in some way') to trial clinical staff. This followed a recommendation from the Trial Steering Committee as a result of one man attempting to take his life during the course of the study.

At the baseline visit men were asked by study staff to note down any medication they were taking. All men who specified that they were taking a medication that has been licensed for treatment of depression were identified.

Other psychosocial measures

For all measures described below, a missing response was considered to indicate absence of the psychosocial factor.

Intimate partner violence

Five questions about emotional, physical, and sexual IPV victimization and IPV perpetration, based on the 'Health and Relationships survey' [15], were asked at month-12 and 24 (see Table 3, footnote [h]).

For peer review only - http://bmjopen.bmj.com/site/about/guidelines.xhtml

Measures of recreational drug use, including sexualized drug use

At baseline, participants were asked to report whether they had used recreational drugs in the past three months. A measure of chemsex was derived, which included the use of at least one of the following drugs: mephedrone, GHB/GBL, or methamphetamine. The phenomenon of chemsex involves the use of these particular drugs before/during sex to enhance sexual experience [18].

At the 12-month and 24-month questionnaires, participants were asked whether, in the past three months, they had engaged in sex after using recreational drugs, which may be a proxy measure for chemsex.

Internalized homophobia

Reporting negative attitudes towards gay sexuality on one or more of the eight questions, from the 26-item Internalized Homophobia Scale [19], asked at month-12 and 24, was considered to be indicative of internalized homophobia (see Table 3 footnote [i]).

Statistical analysis

Prevalence of depressive symptoms (PHQ-9≥10) was compared between trial arms at each time-point using χ^2 tests and across time-points using McNemar's χ^2 test to account for paired responses.

Associations of socio-demographic and lifestyle factors with depressive symptoms (PHQ-9≥10) at baseline were assessed using modified Poisson regression with a robust variance estimator, in order to produce prevalence ratios (PRs) [20]. Generalized estimating equations (GEEs) were used to assess the cross-sectional association of psychosocial factors with depressive symptoms, including all data points from the 12 and 24-month questionnaires. GEEs were fitted using Poisson models with a log link and compound symmetry for the correlational structure.

Associations are presented unadjusted and adjusted for: age, university education, born in the UK, self-reported sexual identity, ongoing relationship, study region (assessed at baseline only, except age), and calendar time. In GEEs, there was no adjustment for 'ongoing relationship' as this status may change over time, and follow-up time-point was additionally adjusted for.

For each measure and at each time-point, the proportion of missing responses was <5%. All analyses were performed in STATA statistical software (version 13) [21] and reported according to the STROBE

For peer review only - http://bmjopen.bmj.com/site/about/guidelines.xhtml

For peer review only

guidelines. A sensitivity analysis was undertaken excluding missing values when defining each variable. The findings were very similar to the main analysis.

Results

There were 544 participants enrolled in PROUD. In total, 540 completed a baseline questionnaire, 410 (76%) completed a 12-month questionnaire, and 333 (62%) completed a 24-month questionnaire (Table 1). Overall, 96% of men identified as gay and 3% as bisexual (includes three trans women). Eighty-two percent of men were of white ethnicity; median age was 35 years (Interquartile range [IQR]: 29-42 years) at baseline (Table 2). Psychosocial characteristics of participants are presented in Table 3. Briefly, lifetime IPV was reported by 45% of participants for victimization and 20% for perpetration, and 42% of participants indicated internalized homophobia at month-12. The prevalence of CAS with ≥2 partners in the past three months was: 76% at baseline, 74% at month-12, and 75% at month-24.

Prevalence of depressive symptoms across trial arms and time-points

The prevalence of depressive symptoms was 9.1% (49/540) at baseline, 14.4% (59/410) at month-12, and 14.4% (48/333) at month-24. There was no difference between men with depressive symptoms and men without, at baseline, in terms of completing the 12-month questionnaire (22.5% vs. 24.2%; χ^2 test p=0.780) and at month-12 in terms of completing the 24-month questionnaire (27.1% vs. 24.8%; χ^2 test p=0.702). Findings were similar when stratifying by trial arm.

The prevalence of depressive symptoms was slightly higher in the immediate than the deferred PrEP group at baseline and at subsequent time-points, but prevalence did not differ significantly between trial arms at months 12 and 24 (Figure 1a). The within-person increase in prevalence observed from baseline to month-12 and from baseline to month-24 was significant (Figure 1b & Table 1a – 1c) but there was no difference from month-12 to month-24.

Overall, 8.5% (46/540) of men reported antidepressant use at baseline; 20.4% (n=10) of men with depressive symptoms (PHQ-9≥10) and 7.3% (n=36) of men without. Eighty five men reported symptoms (PHQ-9≥10) or antidepressant use (15.7% overall), of whom 54.1% (n=46) were receiving antidepressant medication.

For peer review only - http://bmjopen.bmj.com/site/about/guidelines.xhtml

Association of socio-demographic and lifestyle factors with depressive symptoms (PHQ-9≥10) at baseline

Younger men and men who were not employed, reported methamphetamine use in the past three months, and completed their baseline questionnaire before the first quarter of 2013, were more likely to report depressive symptoms in unadjusted and adjusted analysis (see Table 2)

Association of psychosocial factors with depressive symptoms at the 12- and 24-month questionnaire

The prevalence of depressive symptoms was two to three-fold higher among men who reported: ever having been a victim of IPV, IPV victimization in the past year, ever having perpetrated IPV, perpetration of IPV in the past year, internalized homophobia, and not being 'out' to all/almost all friends, work colleagues, and family, in unadjusted and adjusted analysis (see Table 3).

Exploration of changes in depression prevalence

The prevalence of suicidal ideation on PHQ-9 was significantly higher for those who completed their baseline questionnaire before April 1st 2013 (n=71) compared to on or after April 1st 2013, from which time they were asked to disclose their response (n=469) (19.7% vs. 9.6%; Pearson χ^2 test p=0.011). Similarly, the prevalence of depressive symptoms (PHQ-9≥10) was significantly higher among men who completed their baseline questionnaire before April 1st 2013 (21.1% vs. 7.3%; Pearson χ^2 test p<0.001). A possible calendar time effect can also be seen in Table 2, and this relationship remained after adjusting for socio-demographics. There was evidence of an increase in depression from baseline to month-12 for the 353 men with baseline completion on/after April 1st 2013 and 12-month data (7.9% vs. 14.7%), but not for the 57 men with baseline completion before April 1st 2013 and 12-month data (17.5% vs. 12.3%; McNemar's χ^2 test p-values 0.366 and <0.001 respectively). When investigating the modifying effect of baseline completion date on the association between follow-up time-point and depressive symptoms in GEEs, the interaction p-value was 0.004: PR at month-12 vs. baseline, 0.61 95% CI: 0.28, 1.34; PR 1.97 95% CI: 1.41, 2.75, before and after April 1st 2013 respectively.

Discussion

In the PROUD trial of GBMSM reporting very high levels of CAS partners, younger men, those who were not employed, those who reported methamphetamine use, IPV, internalized homophobia, and concealment of sexual identity, were more likely to report depressive symptoms. Depression

For peer review only - http://bmjopen.bmj.com/site/about/guidelines.xhtml

prevalence did not differ significantly between trial arms at any time-point, which was in line with that reported in the iPrEX (placebo controlled) trial [22].

Findings in PROUD suggest that some men may have underreported depressive symptoms at baseline after April 2013. It is possible that newly enrolled participants may have been worried about their inclusion in the trial if they disclosed thoughts of suicide or self-harm to the investigator, and that such concerns may have dissipated over time once participation in the study was established. Patient and public involvement with PROUD participants also highlighted the potential of greater familiarity with the study over time resulting in increased reporting of depressive symptoms at follow-up visits (http://www.proud.mrc.ac.uk/). Other suggested explanations for an actual increase in depressive symptoms included the possibility of increased socio-economic hardship in a period of national financial instability, risk taking behaviours in relation to CAS and chemsex, or PrEP-related stigma. However, we are unable to definitely identify a reason for an actual increase in depressive symptoms after baseline with the available data.

The depression prevalence observed in PROUD at month-12 (14.4%) and 24 (14.4%) is comparable to that found in the AURAH cross-sectional survey of HIV-negative GBMSM attending GUM services in England, 2013-2014 (PHQ-9≥10): 12.4% (n=166/1340) and 15.0% (n=122/815) among GBMSM reporting recent sex (with or without a condom) and recent CAS respectively [14]. Depression prevalence at annual follow-up visits in PROUD was also broadly similar to other studies of GBMSM in the UK [10-15], although comparison is made difficult due to different study settings and measures of depression, and the recruitment of individuals living with HIV in most studies (Textbox 1). However, the prevalence of depressive symptoms observed at follow-up in PROUD, was almost twice that observed in a random sample of men and women in England (2007/2008); 7% reported PHQ-9≥10 [23], although socio-demographic differences may confound this comparison.

In studies that have collected data on depressive symptoms, the proportion of participants reporting any evidence of current depression may be higher when taking into account treatment for depression as well as symptoms. In the PROUD trial, 36 (78%) of the 46 men reporting antidepressant use at baseline had no current depressive symptoms, although there may have been underreporting.

In recent European studies, younger age, markers of lower socio-economic status, lower levels of a supportive network, reporting a bisexual identity [11 14 24], and recreational drug use [14 24], appear to be

For peer review only - http://bmjopen.bmj.com/site/about/guidelines.xhtml

important factors for depression in the GBMSM literature. Mephedrone and crystal methamphetamine use have also been linked to acute symptoms of depression in a qualitative study of 30 gay men in London [25]. Chemsex associated drug use has been found to be strongly associated with depressive symptoms among UK GBMSM in the AURAH study, including after adjustment for socio-demographic factors [14]. Although the pattern of associations was similar in the PROUD trial, some of the differences observed (i.e. between sexual identities, number of recreational drugs used, and any chemsex drug use) were not statistically significant. It may be that due to the possible underreporting of depression at baseline and the limited sample size of this trial, differences that may exist were not detected. It is also important to note that this group consisted of a unique sample of men who all reported very high levels of CAS partners and agreed to participate in an RCT, and therefore associations may differ from other samples of GBMSM.

There is some evidence to suggest that childhood sexual abuse (CSA) is associated with depression [26] among GBMSM in the U.S. In the PROUD trial, a very young age at anal sex debut was not associated with depression. This measure may be a poor proxy for CSA as it is not likely to reflect or pick up all sexual abuse experienced. Strong associations were found of IPV victimization and perpetration with depressive symptoms in the PROUD trial. Evidence from a recent meta-analysis of mostly U.S. studies suggests that IPV is associated with depression among GBMSM [27]. One UK study (GUM clinic attendees, 2010-2011) has investigated this link and found that depression (HADS≥8) was significantly elevated among men reporting IPV perpetration in the past year (20.7% vs. 11.5%), but the association was attenuated after adjusting for socio-demographics (OR 3.7 95% CI: 1.0, 14.6; $p=0.060$) [15]. Adjusting for income may have resulted in the attenuation and obscured the association. Depression is highly correlated with markers of financial security and financial inequalities within intimate partnerships may increase the risk of controlling and abusive behaviours. It was not possible to investigate the confounding effect of income/financial security in PROUD.

In Meyer's minority stress model, perpetual negative feedback from others, (direct or indirect) homophobic stereotyping and prejudice, can lead to internalized homophobia, pervasive expectations of rejection, and non-disclosure of one's sexual orientation, with implications for poor mental health outcomes [28]. In a recent qualitative study of GBMSM in the UK (N=21), several men described negative and traumatic experiences surrounding the disclosure of a sexual minority status, which led to feelings of guilt or depression [29]. Similarly, in PROUD, a measure of internalized homophobia and a lower degree of disclosure of sexual orientation was strongly associated with depressive symptoms.

For peer review only - http://bmjopen.bmj.com/site/about/guidelines.xhtml

Strengths and limitations

This is the first UK study to collect longitudinal data on depression prevalence over time among GBMSM, and investigate associations with a range of psychosocial factors. There was no explicit measure of chemsex in this paper. The examination of factors associated with depression was based on cross-sectional analysis, utilising all available observations. Direction of associations may be unclear for some factors, such as drug use or IPV. For this reason, as well as the possibility of unmeasured confounders, inferences regarding causal factors for depression are limited.

Conclusions

Our findings add to the growing body of research in other high-income countries suggesting a high concomitant burden of sexual minority related stress, substance use, and IPV with depression among GBMSM. Since these psychosocial measures are relevant to GBMSM at high-risk of HIV and seeking PrEP, it is recommended that training on awareness and enquiry about psychosocial issues should be enhanced in sexual health services.

Ethics approval and consent to participate

The study was reviewed and approved by London Bridge Research Ethics Committee (12/LO/1289). Written informed consent was obtained from all participants. The study protocol is available online (http://www.proud.mrc.ac.uk/about/study-protocol/).

Data Sharing Statement

The PROUD data is held at MRC CTU at UCL, which encourages optimal use of data by employing a controlled access approach to data sharing, incorporating a transparent and robust system to review requests and provide secure data access consistent with the relevant ethics committee approvals. All requests for data are considered and can be initiated by contacting proud.mrcctu@ucl.ac.uk.

Competing interests

The PROUD study was provided drug free of charge by Gilead Sciences plc. which also distributed it to participating clinics and provided funds for additional diagnostic tests for HCV and drug levels. AP has received payments for presentations made at meetings sponsored by Gilead in spring 2015. EW has had tuition fees and a stipend paid by Gilead. AC received advisory board fees from Gilead Sciences plc. and GSK/ViiV; speaker fees from Gilead and conferences bursaries from Gilead & Janssen. SM reports grants from the European Union H2020 scheme, EDCTP 2, the National Institute

For peer review only - http://bmjopen.bmj.com/site/about/guidelines.xhtml

For peer review only

of Health Research, and Gilead Sciences; other support from Gilead Sciences, and the Population Council Microbicide Advisory Board; and is Chair of the Project Advisory Committee for USAID grant awarded to CONRAD to develop tenofovir-based products for use by women (non-financial).

Authors' contributions

Conceived and designed the study: MG FCL ARM SM DD EW AR AP LS AC AKS IR. Analyzed the data: ARM FCL. Wrote the paper: ARM MG FCL. All authors read and approved the final manuscript.

Funding

Sheena McCormack and David Dunn were supported by a Medical Research Council grant (MRC_UU_12023/23).

Acknowledgements

We thank all PROUD trial participants.

The PROUD study group:

David I. Dolling, Monica Desai, Alan McOwan, Richard Gilson, Amanda Clarke, Martin Fisher, Gabriel Schembri, Ann K. Sullivan, Nicola Mackie, Iain Reeves, Mags Portman, Vanessa Apea, John Saunders, Julie Fox, Jake Bayley, Michael Brady, Killian Quinn, Christine Bowman, Clarie Dewsnap, Charles J. Lacey, Stephen Taylor, David White, Simone Antonucci, Mitzy Gafos, Sheena McCormack, Owen N. Gill, David T. Dunn, and Anthony Nardone.

PROUD clinic teams:

Drew Clark, Paul Davis, James Hand, Machel Hunt, Rebecca Neale, Jackie O'Connell, Liat Sarner, John Saunders, Louise Terry, Angelina Twumasi, Salina Tsui, Dayan Vijeratnam, Ryan Whyte, Andy Williams, Sian Gately, Gerry Gilleran, Jill Lyons, Chris McCormack, Katy Moore, Cathy Stretton, Alex Acheampong, Michael Bramley, Marion Campbell, Ruby Chowdhry, Stewart Eastwood, Babs Fennell, Wendy Hadley, Kerry Hobbs, Sarah Kirk, Nicky Perry, Charlotte Rawlinson, Celia Richardson, Claire Richardson, Mark Roche, Emma Simpkin, Simon Shaw, Elisa Souto, Julia Williams, Elaney Youssef, Tristan Barber, Cindy Eliot, Serge Fedele, Chris Higgs, Kathryn McCormick, Alexandra Meijer, Sam Pepper, Jane Rowlands, Gurmit Singh, Alfredo SolerCarracedo, Sonali Sonecha, David Taylor, Lervina Thomas, Frederick Attakora, Marina Bourke, Richard Castles, Rebecca Clark, Anke De-Masi, Veronica Espa, Rumbidzai Hungwe, Martin Lincoln, Sifiso Mguni, Rhianon Nevin-Dolan, Hannah Alexander, Lucy Campbell, Sophie Candfield, Shema Doshi, Olivia Liddle, Larissa Mulka, Priyanka Saigal, James

For peer review only - http://bmjopen.bmj.com/site/about/guidelines.xhtml

For peer review only

Stevenson, James Boateng, Brynn Chappell, Susanna Currie, Carolyn Davies, Dornubari Lebari, Matthew Phillips, Lisa Southon, Sarah Thorpe, Anna Vas, Chris Ward, Claire Warren, Stephanie Yau , Alejandro Arenas-Pinto, Asma Ashraf, Matthew Bolton, Lewis Haddow, Sara McNamara, Ana Milinkovic, June Minton, Dianne Morris, Clare Oakland, Steve O'Farrell, Pierre Pellegrino, Sarah Pett, Nina Vora, Carmel Young, Taris Zarko-Flynn, Wilbert Ayap, Ling Jun Chen, Adam Croucher, Sarah Fidler, Kristin Kuldanek, Ken Legg, Agathe Leon, Nadia Naous, Severine Rey, Judith Zhou, Margaret-Anne Bevan, Nina Francia, Eleanor Hamlyn, Lisa Hurley, Helen Iveson, Isabelle Jendrulek, Tammy Murray, Alice Sharp, Andrew Skingsley, Chi Kai Tam, Al Teague, Caroline Thomas, Juan-Manuel Tiraboschi, Christine Brewer, Richard Evans, Jan Gravely, Gary Lamont, Fabiola Martin, Georgina Morris, Sarah Russell-Sharpe, John Wightman, Anthony Bains, Gill Bell, Terry Cox, Charlie Hughes, Hannah Loftus, Naomi Sutton, Debbie Talbot, Vince Tucker.

For peer review only

Table 1a: Changes in number of men reporting depressive symptoms (PHQ-9≥10) from baseline to the 12-month questionnaire

Depressive symptoms (PHQ-9≥10) at baseline:	Depressive symptoms (PHQ-9≥10) at month-12:		
	No	Yes	Total number of men
No	335 (90.1%)	37 (10.0%)	372 (100.0%)
Yes	16 (42.1%)	22 (57.9%)	38 (100.0%)
Total number of men	351 (85.6%)	59 (14.4%)	410

Table 1b: Changes in number of men reporting depressive symptoms (PHQ-9≥10) from baseline to the 24-month questionnaire

Depressive symptoms (PHQ-9≥10) at baseline:	Depressive symptoms (PHQ-9≥10) at month-24:		
	No	Yes	Total number of men
No	264 (87.7%)	37 (12.3%)	301 (100.0%)
Yes	21 (65.6%)	11 (34.4%)	32 (100.0%)
Total number of men	285 (85.6%)	48 (14.4%)	333

Table 1c: Changes in number of men reporting depressive symptoms (PHQ-9≥10) from the 12- to 24-month questionnaire

Depressive symptoms (PHQ-9≥10) at month-12:	Depressive symptoms (PHQ-9≥10) at month-24:		
	No	Yes	Total number of men
No	235 (89.0%)	29 (11.0%)	264 (100.0%)
Yes	27 (62.8%)	16 (37.2%)	43 (100.0%)
Total number of men	262 (85.3%)	45 (14.7%)	307

For peer review only - http://bmjopen.bmj.com/site/about/guidelines.xhtml

Table 2: Unadjusted and adjusted associations of socio-demographic and lifestyle factors with depressive symptoms at baseline in PROUD

N=540 men at baseline			Depressive symptoms (PHQ-9≥10)					
		N (%)	%	*p-value*[a]	Unadjusted PR [95% CI]	Overall *p-value*[d]	Adjusted[e] PR [95% CI]	*Overall p-value*[d]
Calendar time (year & calendar quarter [Q])[f]	2012 Q4 – 2013 Q1	71 (13.2%)	21.1%	*0.001*	1	*0.003*	1	*0.004*
	2013 Q2	85 (15.7%)	2.4%	*0.20*[b]	0.11 [0.03, 0.47]	*0.25*[b]	0.12 [0.03, 0.47]	*0.26*[b]
	2013 Q3	113 (20.9%)	7.1%		0.34 [0.15, 0.75]		0.34 [0.15, 0.76]	
	2013 Q4	119 (22.0%)	8.4%		0.40 [1.19, 0.84]		0.39 [0.18, 0.83]	
	2014 Q1 – Q2	152 (28.2%)	9.2%		0.44 [0.22, 0.85]		0.45 [0.23, 0.88]	
Age	<25	54 (10.0%)	22.2%	*0.003*	3.63 [1.44, 9.13]	*0.006*	3.49 [1.44, 8.44]	*0.003*
	25-29	96 (17.8%)	8.3%	*0.002*[b]	1.36 [0.49, 3.78]	*0.003*[b]	1.22 [0.43, 3.51]	*0.004*[b]
	30-34	114 (21.1%)	12.3%		2.01 [0.80, 5.02]		1.97 [0.78, 4.97]	
	35-39	97 (18.0%)	6.2%		1.01 [0.34, 3.03]		0.96 [0.31, 3.00]	
	40-44	81 (15.0%)	3.7%		0.60 [0.16, 2.35]		0.60 [0.16, 2.31]	
	45+	98 (18.2%)	6.1%		1		1	
Born in the UK and white ethnicity	Yes, white	287 (53.4%)	9.1%	*0.32*[c]	1	*0.37*	1	*0.44*
	Yes, BAME	35 (6.5%)	14.3%		1.58 [0.65, 3.84]		0.93 [0.41, 2.09]	
	No, white	152 (28.3%)	6.6%		0.73 [0.36, 1.47]		0.77 [0.36, 1.65]	
	No, BAME	64 (11.9%)	12.5%		1.38 [0.65, 2.91]		1.59 [0.72, 3.54]	
Self-reported sexual identity[g]	Gay	513 (95.7%)	9.0%	*0.46*[c]	1	*0.50*	1	*0.64*
	Bisexual	17 (3.2%)	13.0%		1.45 [0.49, 4.33]		1.26 [0.48, 3.31]	
	Straight	6 (1.1%)						
University Education	Yes	327 (60.6%)	8.9%	*0.84*	1	*0.84*	1	*0.94*
	No/missing	213 (39.4%)	9.4%		1.06 [0.61, 1.82]		0.98 [0.54, 1.77]	
Employed	Yes	439 (81.3%)	6.8%	*<0.001*	1	*<0.001*	1	*0.001*
	No/missing	101 (18.7%)	18.8%		2.75 [1.62, 4.69]		2.52 [1.45, 4.37]	
Ongoing relationship	Yes	246 (45.6%)	6.5%	*0.06*	1	*0.06*	1	*0.15*
	No/missing	294 (54.4%)	11.2%		1.73 [0.97, 3.06]		1.50 [0.86, 2.63]	
Study region	London	375 (69.4%)	9.3%	*0.75*	1	*0.75*	1	*0.66*
	Outside London	165 (30.6%)	8.5%		0.91 [0.50, 1.64]		0.86 [0.44, 1.69]	
Higher-risk drinking[h]	No/missing	367 (68.0%)	8.5%	*0.46*	1	*0.46*	1	*0.48*
	Yes	173 (32.0%)	10.4%		1.23 [0.71, 2.14]		1.25 [0.68, 2.29]	

For peer review only

For peer review only - http://bmjopen.bmj.com/site/about/guidelines.xhtml

Table 2: Unadjusted and adjusted associations of socio-demographic and lifestyle factors with depressive symptoms at baseline in PROUD (continued)

N=540 men at baseline			Depressive symptoms (PHQ-9≥10)					
		N (%)	%	*p-value*[a]	Unadjusted PR [95% CI]	Overall *p-value*[d]	Adjusted[e] PR [95% CI]	Overall *p-value*[d]
Recreational drug use (past 3 months)	0/missing<br>1<br>2-4<br>5+	148 (27.4%)<br>87 (16.1%)<br>159 (29.4%)<br>146 (27.0%)	10.1%<br>4.6%<br>6.9%<br>13.0%	*0.11*<br>*0.40*[b]	1<br>0.45 [0.16, 1.32]<br>0.68 [0.32, 1.44]<br>1.28 [0.68, 2.43]	0.13<br>*0.45*[b]	1<br>0.57 [0.20, 1.64]<br>0.89 [0.39, 2.02]<br>1.34 [0.71, 2.56]	0.32<br>*0.32*[b]
Chemsex-associated drug use[i] (crystal meth, mephedrone or GHB/GBL in past 3 months)	No/missing<br>Yes	309 (57.2%)<br>231 (42.8%)	8.1%<br>10.4%	*0.36*	1<br>1.28 [0.75, 2.19]	*0.36*	1<br>1.31 [0.77, 2.23]	*0.33*
Crystal meth (past 3 months)	No/missing<br>Yes	442 (81.9%)<br>98 (18.2%)	7.7%<br>15.3%	*0.02*	1<br>1.99 [1.13, 3.51]	*0.02*	1<br>1.95 [1.06, 3.61]	*0.03*
Mephedrone (past 3 months)	No/missing<br>Yes	343 (63.5%)<br>197 (36.5%)	9.0%<br>9.1%	*0.97*	1<br>1.01 [0.58, 1.76]	*0.97*	1<br>0.99 [0.56, 1.73]	*0.96*
GHB/GBL (past 3 months)	No/missing<br>Yes	371 (68.7%)<br>169 (31.3%)	8.4%<br>10.7%	*0.39*	1<br>1.27 [0.73, 2.21]	*0.39*	1<br>1.23 [0.72, 2.11]	*0.46*
Ketamine (past 3 months)	No/missing<br>Yes	451 (83.5%)<br>89 (16.5%)	8.4%<br>12.4%	*0.24*	1<br>1.47 [0.78, 2.76]	*0.24*	1<br>1.43 [0.76, 2.71]	*0.27*
Cocaine (past 3 months)	No/missing<br>Yes	401 (74.3%)<br>139 (25.7%)	8.5%<br>10.8%	*0.41*	1<br>1.27 [0.72, 2.27]	*0.41*	1<br>1.14 [0.66, 1.97]	*0.64*
Ecstasy/MDMA (past 3 months)	No/missing<br>Yes	450 (83.3%)<br>90 (16.7%)	8.4%<br>12.2%	*0.26*	1<br>1.45 [0.77, 2.72]	*0.25*	1<br>1.31 [0.69, 2.49]	*0.41*

BAME= Black, Asian and minority ethnic.
[a] Pearson χ^2 test.
[b] Test for trend.
[c] Fisher's exact test.
[d] p-value by Wald test using modified Poisson regression with a robust variance estimator in order to produce adjusted prevalence ratios.
[e] Age (included as four categories: <25, 25-29, 30-39, 40+), born in the UK, sexual identity (gay or bisexual/straight), university education, relationship status, London study clinic site, and calendar time.
[f] Some calendar quarters (Q4 2012 & Q1 2013; Q1-Q2 2014) were combined due to small cell counts.
[g] A dichotomized version of self-reported sexual identity is investigated in analysis due to very small numbers: gay or bisexual/straight.

For peer review only - http://bmjopen.bmj.com/site/about/guidelines.xhtml

[h] Based on the first two questions of the WHO AUDIT-C questionnaire [30]: higher-risk score of ≥6 or lower risk/no alcohol consumption score of <6 (including a missing response to both questions). Of note, the response options included in PROUD for the first WHO AUDIT-C question ('How often have you had a drink containing alcohol in the last 90 days?') were 1 frequency lower than that specified in the WHO questionnaire, for instance, '2 or 3 times a month' instead of '2 to 4 times a month', and 'once or twice a week' instead of '2 to 3 times a week'. It is therefore possible, that the prevalence of higher-risk alcohol consumption in PROUD may be slightly overestimated.
[i] Chemsex is the use of psychoactive substances during/immediately before sex to stimulate sexual arousal [18]. It is of note, however, that men were not asked in PROUD whether these drugs had been taken before or during sex.

Table 3: Unadjusted and adjusted associations of psychosocial factors with depressive symptoms using 12- and 24-month PROUD questionnaire data in GEE models

				Depressive symptoms (PHQ-9≥10)			
N=436 men, observations=743 (using data from men who completed the 12-month or 24-month questionnaire in GEE models)		12-month [N=410] n(%)	24-month [N=333] n(%)	Unadjusted PR [95% CI]	Overall p-value[a]	Adjusted[b] PR [95% CI]	Overall p-value[a]
Had sex after using recreational drugs (past 3 months)<br>N=436[c], Obs=743[d] ; N=432[e], Obs=737[f]	No/missing<br>Yes	186 (45.4%)<br>224 (54.6%)	174 (52.3%)<br>159 (47.8%)	1<br>1.38 [0.93, 2.06]	*0.11*	1<br>1.38 [0.92, 2.07]	*0.12*
Age≤15 years at anal sex debut<br>N=433[c], Obs=716[d] ; N=429[e], Obs=710[f]	No<br>Yes	307 (77.7%)<br>88 (22.3%)	251 (78.2%)<br>70 (21.8%)	1<br>1.27 [0.80, 2.02]	*0.31*	1<br>1.20 [0.75, 1.94]	*0.45*
Age<13 years at anal sex debut [g]<br>N=433[c], Obs=716[d] ; N=429[e], Obs=710[f]	No<br>Yes	373 (94.4%)<br>22 (5.6%)	302 (94.1%)<br>19 (5.9%)	1<br>0.71 [0.26, 1.94]	0.51	1<br>0.72 [0.27, 1.97]	*0.526*
Any IPV victimization [h]<br>N=436[c], Obs=743[d] ; N=432[e], Obs=737[f]	No/missing<br>Yes	226 (55.1%)<br>184 (44.9%)	199 (59.8%)<br>134 (40.2%)	1<br>2.45 [1.63, 3.67]	*<0.001*	1<br>2.56 [1.70, 3.85]	*<0.001*
Any IPV victimization in the past year [h]<br>N=436[c], Obs=743[d] ; N=432[e], Obs=737[f]	No/missing<br>Yes	346 (84.4%)<br>64 (15.6%)	284 (85.3%)<br>49 (14.7%)	1<br>2.82 [1.88, 4.22]	*<0.001*	1<br>2.96 [1.97, 4.46]	*<0.001*
Any IPV perpetration [h]<br>N=436[c], Obs=743[d] ; N=432[e], Obs=737[f]	No/missing<br>Yes	330 (80.5%)<br>80 (19.5%)	273 (82.0%)<br>60 (18.0%)	1<br>2.83 [1.89, 4.22]	*<0.001*	1<br>2.86 [1.90, 4.30]	*<0.001*
Any IPV perpetration in the past year [h]<br>N=436[c], Obs=743[d] ; N=432[e], Obs=737[f]	No/missing<br>Yes	378 (92.2%)<br>32 (7.8%)	310 (93.1%)<br>23 (6.9%)	1<br>3.40 [2.13, 5.41]	*<0.001*	1<br>3.36 [2.06, 5.47]	*<0.001*
Any negative views about gay sexuality [i] (marker of internalised homophobia)<br>N=436[c], Obs=743[d] ; N=432[e], Obs=737[f]	No/missing<br>Yes	239 (58.3%)<br>171 (41.7%)	195 (58.6%)<br>138 (41.4%)	1<br>1.92 [1.30, 2.83]	*0.001*	1<br>1.91 [1.28, 2.83]	*0.001*
'Out' to all/almost all friends, work colleagues and close family [j]<br>N=434[c], Obs=727[d] ; N=431[e], Obs=722[f]	Yes<br>No	206 (51.6%)<br>193 (48.4%)	166 (50.6%)<br>162 (49.4%)	1<br>1.75 [1.17, 2.62]	*0.007*	1<br>1.71 [1.13, 2.59]	*0.012*

[a] p-value by Wald test using GEE models.

For peer review only

For peer review only - http://bmjopen.bmj.com/site/about/guidelines.xhtml

[b] Age (included as four categories: <25, 25-29, 30-39, 40+), born in the UK, sexual identity (gay or bisexual/straight), university education, London study clinic site, calendar time (year & calendar quarter), and follow-up time-point (month-12, month-24).
[c] Number of men contributing observations to the unadjusted model.
[d] Number of observations examined in the unadjusted model.
[e] Number of men contributing observations to the adjusted model.
[f] Number of observations examined in the adjusted model.
[g] Reporting anal intercourse at a very young age was investigated since this measure may include experiences of childhood sexual abuse (CSA). However, since it was not known whether the male sexual debut partner with whom the participant referred was an adult/significantly older and if they felt forced, there was most likely measurement error when attempting to capture experiences of CSA among PROUD participants.
[h] Participants were asked five questions about IPV victimization and five questions about IPV perpetration. Each question had the following four response options: never, yes- more than 1 year ago, yes within the last year with former partner, and yes within the last year with current partner. The five IPV victimization questions included the following: 'Have you ever felt frightened of the behaviour of a partner', 'Have you ever needed to ask a partner's permission to work, go shopping, visit relatives, or visit friends (i.e. beyond the usual of being considerate to and checking with a partner)', 'Have you ever been hit, slapped, kicked or otherwise physically hurt by a partner', 'Have you ever been forced to have sex or made to engage in some sexual activity when you did not want to', and 'Have you ever been forced to have sex without a condom when you did not want to'. The five IPV perpetration questions were identical but asked whether the participant had perpetrated these behaviours. A positive response to any one of the five respective questions was considered to indicate lifetime IPV victimization or lifetime IPV perpetration.
[i] Participants were asked eight questions surrounding attitudes towards gay sexuality based on the 26-item Internalized Homophobia Scale (IHS) [19]. Each question had the following five response options: strongly agree, agree, neutral or uncertain, disagree, and strongly disagree. The eight questions included the following: (i) 'I feel comfortable in gay bars', (ii) 'I feel comfortable being seen in public with an obviously transgender/gay person', (iii) 'I feel comfortable discussing homosexuality in a public situation', (iv)'I feel comfortable being a transgender/gay man', (v) 'Homosexuality is morally acceptable to me', (vi) 'Even if I could change my sexual orientation I wouldn't', (vii) 'Obviously effeminate homosexual men make me feel uncomfortable' and (viii) 'Social situations with transgender/gay men make me feel uncomfortable'. Responding with 'strongly agree' or 'agree' to questions (vii) or (viii), or with 'strongly disagree' or 'disagree' to questions (i), (ii), (iii), (iv), (v), or (vi) was considered to indicate having negative attitudes towards gay sexuality.
[j] Participants were asked how many of their (i) work colleagues, (ii) friends, and (iii) close family, know that they are gay/transgender/have sex with men. Response options for groups (i)-(iii) included: all/almost all, >half, <half, few, and none.

For peer review only - http://bmjopen.bmj.com/site/about/guidelines.xhtml

Textbox 1: Prevalence of depression on symptom questionnaires in studies of UK GBMSM

Study	Recruitment dates	Sampling strategy	Size of GBMSM sample	Measure of depression	Depression prevalence
Cross-sectional survey of GBMSM in London[13]	1999	Consecutive sampling of GBMSM attending a GUM clinic	122	HADS score of ≥8	18.8%
Cross-sectional survey of GBMSM in London[15]	2010-2011	Consecutive sampling of GBMSM attending GUM clinics	519	HADS score of ≥8	12%
Natsal-3[10]	2010-2012	Multistage probability sampling of the general British population	190	PHQ-2 score of ≥3	8.9%
Cross-sectional survey of GBMSM in London[12]	2016	Volunteer sampling of users of a geosocial-networking app for GBMSM	179	PHQ-2 score of ≥3	22.3%
Gay & Bisexual Men's Health Survey[11]	2011	Snowball sampling of GBMSM	5416	PHQ-9 score of ≥10	21.3%
AURAH[14]	2013-2014	Consecutive sampling of HIV-negative GBMSM attending GUM clinics in England	1340 sexually active GBMSM	PHQ-9 score of ≥10	12.4%
PROUD	2012-2014 baseline<br>2013-2015 month-12<br>2014-2016 month-24	Volunteer sampling of HIV-negative GBMSM attending GUM clinics and community organizations, and GBMSM users of social media	540<br>410<br>333	PHQ-9 score of ≥10	9.1%<br>14.4%<br>14.4%

For peer review only

For peer review only - http://bmjopen.bmj.com/site/about/guidelines.xhtml

References:

1. Beck AT. The evolution of the cognitive model of depression and its neurobiological correlates. *Am J Psychiatry* 2008;165(8):969-77. doi: 10.1176/appi.ajp.2008.08050721 [published Online First: 2008/07/17]
2. McManus S, Bebbington P, Jenkins R, et al. Mental Health and Wellbeing in England: Adult Psychiatric Morbidity Survey 2014. Leeds: NHS Digital, 2016.
3. Bandura A. Self-efficacy. The exercise of control. New York: W.H. Freeman and Company 1997.
4. McKirnan DJ, Ostrow DG, Hope B. Sex, drugs and escape: a psychological model of HIV-risk sexual behaviours. *AIDS Care* 1996;8(6):655-69. doi: 10.1080/09540129650125371 [published Online First: 1996/12/01]
5. Herek GM, Sims C, editors. *Sexual orientation and violent victimization: hate crimes and intimate partner violence among gay and bisexual males in the United States*. New York: Oxford University Press, 2008.
6. Chen YH, Raymond HF. Associations between depressive syndromes and HIV risk behaviors among San Francisco men who have sex with men. *AIDS Care* 2017:1-5. doi: 10.1080/09540121.2017.1307925 [published Online First: 2017/04/04]
7. Millar BM, Starks TJ, Grov C, et al. Sexual Risk-Taking in HIV-Negative Gay and Bisexual Men Increases with Depression: Results from a U.S. National Study. *AIDS Behav* 2017;21(6):1665-75. doi: 10.1007/s10461-016-1507-6 [published Online First: 2016/08/01]
8. Parsons JT, Grov C, Golub SA. Sexual compulsivity, co-occurring psychosocial health problems, and HIV risk among gay and bisexual men: further evidence of a syndemic. *Am J Public Health* 2012;102(1):156-62. doi: 10.2105/ajph.2011.300284 [published Online First: 2011/11/19]
9. Semlyen J, King M, Varney J, et al. Sexual orientation and symptoms of common mental disorder or low wellbeing: combined meta-analysis of 12 UK population health surveys. *BMC Psychiatry* 2016;16:67.
10. Mercer CH, Prah P, Field N, et al. The health and well-being of men who have sex with men (MSM) in Britain: Evidence from the third National Survey of Sexual Attitudes and Lifestyles (Natsal-3). *BMC Public Health* 2016;16(525) doi: 10.1186/s12889-016-3149-z
11. Hickson F, Davey C, Reid D, et al. Mental health inequalities among gay and bisexual men in England, Scotland and Wales: a large community-based cross-sectional survey. *J Public Health (Oxf)* 2017;39(2):266-73. doi: doi:10.1093/pubmed/fdw021
12. Goedel WC, Hagen D, Halkitis PN, et al. Post-exposure prophylaxis awareness and use among men who have sex with men in London who use geosocial-networking smartphone applications. *AIDS Care* 2017;29(5):579-86. doi: 10.1080/09540121.2016.1259455 [published Online First: 2016/12/03]
13. Beck A, McNally I, Petrak J. Psychosocial predictors of HIV/STI risk behaviours in a sample of homosexual men. *Sex Transm Infect* 2003;79(2):142-6. [published Online First: 2003/04/12]
14. Miltz AR, Rodger AJ, Sewell J, et al. Clinically significant depressive symptoms and sexual behaviour among men who have sex with men. *The British Journal of Psychiatry Open* 2017;3(3):127-37. doi: 10.1192/bjpo.bp.116.003574 [published Online First: 2017/05/17]
15. Bacchus LJ, Buller AM, Ferrari G, et al. Occurrence and impact of domestic violence and abuse in gay and bisexual men: A cross sectional survey. *Int J STD AIDS* 2017;28(1):16-27. doi: 10.1177/0956462415622886 [published Online First: 2016/01/09]
16. McCormack S, Dunn DT, Desai M, et al. Pre-exposure prophylaxis to prevent the acquisition of HIV-1 infection (PROUD): effectiveness results from the pilot phase of a pragmatic open-label randomised trial. *Lancet* 2016;387(10013):53-60. doi: 10.1016/s0140-6736(15)00056-2 [published Online First: 2015/09/14]
17. Kroenke K, Spitzer RL, Williams JB. The PHQ-9: validity of a brief depression severity measure. *J Gen Intern Med* 2001;16(9):606-13. [published Online First: 2001/09/15]

For peer review only - http://bmjopen.bmj.com/site/about/guidelines.xhtml

18. Weatherburn P, Hickson F, Reid D, et al. Motivations and values associated with combining sex and illicit drugs ('chemsex') among gay men in South London: findings from a qualitative study. *Sex Transm Infect* 2017;93:153-54.
19. Ross MW, Rosser BR. Measurement and correlates of internalized homophobia: a factor analytic study. *J Clin Psychol* 1996;52(1):15-21. doi: 10.1002/(sici)1097-4679(199601)52:1<15::aid-jclp2>3.0.co;2-v [published Online First: 1996/01/01]
20. Zou G. A modified poisson regression approach to prospective studies with binary data. *Am J Epidemiol* 2004;159(7):702-6. [published Online First: 2004/03/23]
21. STATA statistical software. 13 ed [program]. Texas, 2009.
22. Defechereux PA, Mehrotra M, Liu AY, et al. Depression and Oral FTC/TDF Pre-exposure Prophylaxis (PrEP) Among Men and Transgender Women Who Have Sex With Men (MSM/TGW). *AIDS Behav* 2016;20(7):1478-88. doi: 10.1007/s10461-015-1082-2 [published Online First: 2015/06/17]
23. Paranjothy S, Gallacher J, Amlot R, et al. Psychosocial impact of the summer 2007 floods in England. *BMC Public Health* 2011;11:145. doi: 10.1186/1471-2458-11-145 [published Online First: 2011/03/05]
24. Vanden Berghe W, Nostlinger C, Laga M. Syndemic and other risk factors for unprotected anal intercourse among an online sample of Belgian HIV negative men who have sex with men. *AIDS Behav* 2014;18(1):50-8. doi: 10.1007/s10461-013-0516-y [published Online First: 2013/05/18]
25. Bourne A, Reid D, Hickson F, et al. "Chemsex" and harm reduction need among gay men in South London. *Int J Drug Policy* 2015;26(12):1171-6. doi: 10.1016/j.drugpo.2015.07.013 [published Online First: 2015/08/25]
26. Mimiaga MJ, Noonan E, Donnell D, et al. Childhood sexual abuse is highly associated with HIV risk-taking behavior and infection among MSM in the EXPLORE Study. *J Acquir Immune Defic Syndr* 2009;51(3):340-8. doi: 10.1097/QAI.0b013e3181a24b38 [published Online First: 2009/04/16]
27. Buller AM, Devries KM, Howard LM, et al. Associations between intimate partner violence and health among men who have sex with men: a systematic review and meta-analysis. *PLoS Med* 2014;11(3):e1001609. doi: 10.1371/journal.pmed.1001609 [published Online First: 2014/03/07]
28. Meyer IH. Prejudice, social stress, and mental health in lesbian, gay, and bisexual populations: conceptual issues and research evidence. *Psychol Bull* 2003;129(5):674-97. doi: 10.1037/0033-2909.129.5.674 [published Online First: 2003/09/06]
29. Gourlay A, Fox J, Gafos M, et al. A qualitative study exploring the social and environmental context of recently acquired HIV infection among men who have sex with men in South-East England. *BMJ Open* 2017;7(8):e016494. doi: 10.1136/bmjopen-2017-016494 [published Online First: 2017/08/31]
30. Babor TF, Higgins-Biddle JC, Saunders JB, et al. The Alcohol Use Disorders Identification Test. Guidelines for Use in Primary Care: Department of Mental Health and Substance Dependence, World Health Organization, 2001.

For peer review only

For peer review only - http://bmjopen.bmj.com/site/about/guidelines.xhtml

Figure 1a: Prevalence of depressive symptoms (PHQ-9≥10) by trial arm and time-point, among all men with data at each time-point in PROUD

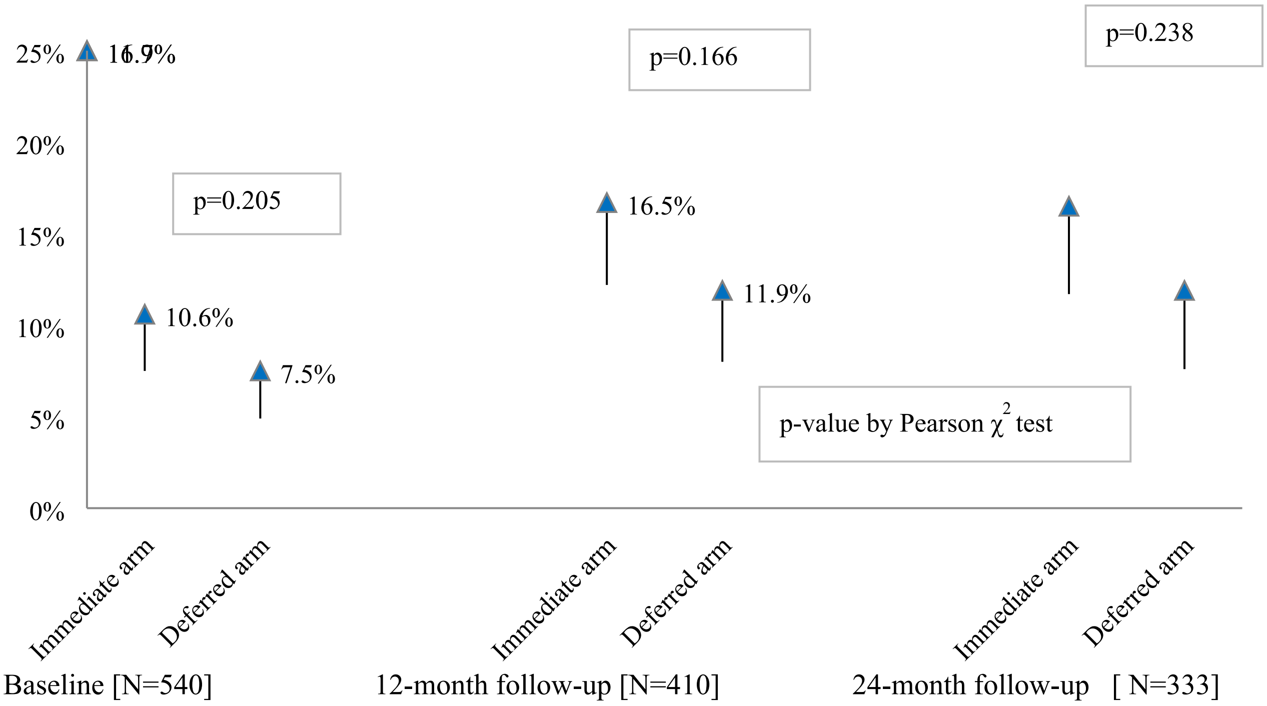

eview only

Figure 1b: Changes in prevalence of depressive symptoms (PHQ-9≥10) over time among men with data at two time-points (randomised groups are combined) in PROUD

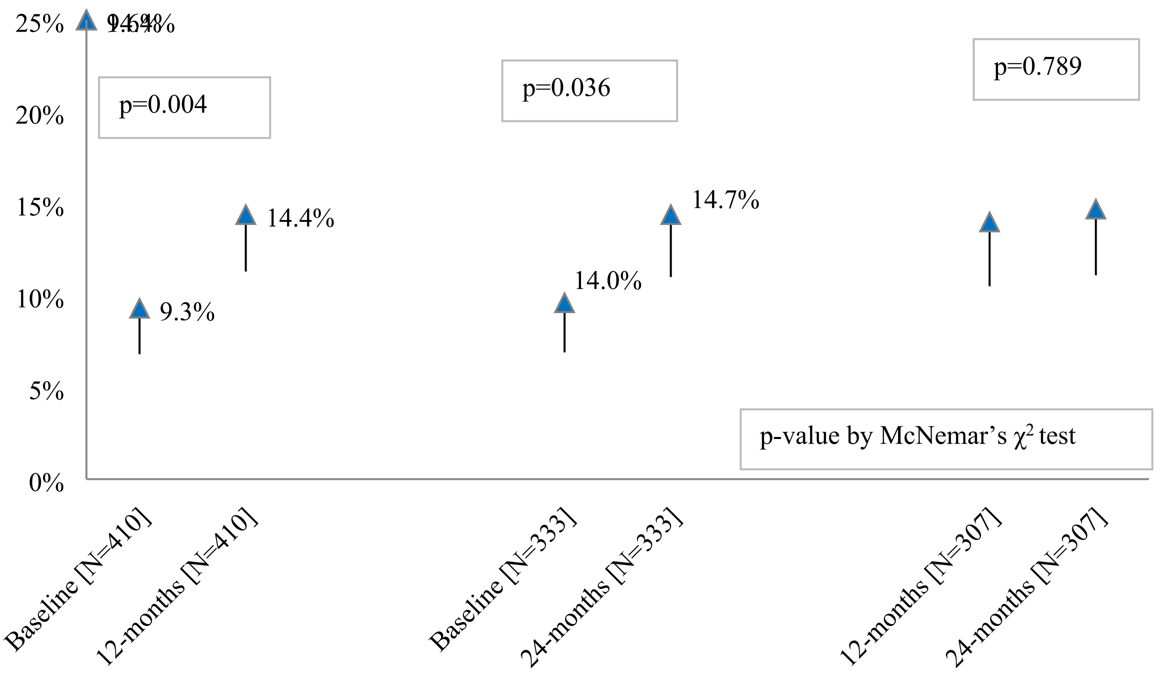

view only

STROBE Statement—Checklist of items that should be included in reports of ***cross-sectional studies***

	Item No	Recommendation	Page No
**Title and abstract**	1	(*a*) Indicate the study's design with a commonly used term in the title or the abstract	1-2
		(*b*) Provide in the abstract an informative and balanced summary of what was done and what was found	2
**Introduction**			
Background/rationale	2	Explain the scientific background and rationale for the investigation being reported	3-4
Objectives	3	State specific objectives, including any prespecified hypotheses	4
**Methods**			
Study design	4	Present key elements of study design early in the paper	5-8
Setting	5	Describe the setting, locations, and relevant dates, including periods of recruitment, exposure, follow-up, and data collection	5
Participants	6	(*a*) Give the eligibility criteria, and the sources and methods of selection of participants	5
Variables	7	Clearly define all outcomes, exposures, predictors, potential confounders, and effect modifiers. Give diagnostic criteria, if applicable	5-7
Data sources/ measurement	8*	For each variable of interest, give sources of data and details of methods of assessment (measurement). Describe comparability of assessment methods if there is more than one group	5-7
Bias	9	Describe any efforts to address potential sources of bias	7-8
Study size	10	Explain how the study size was arrived at	5
Quantitative variables	11	Explain how quantitative variables were handled in the analyses. If applicable, describe which groupings were chosen and why	7-8
Statistical methods	12	(*a*) Describe all statistical methods, including those used to control for confounding	7-8
		(*b*) Describe any methods used to examine subgroups and interactions	/
		(*c*) Explain how missing data were addressed	5-8
		(*d*) If applicable, describe analytical methods taking account of sampling strategy	7-8
		(*e*) Describe any sensitivity analyses	8
**Results**			
Participants	13*	(a) Report numbers of individuals at each stage of study—eg numbers potentially eligible, examined for eligibility, confirmed eligible, included in the study, completing follow-up, and analysed	8-9
		(b) Give reasons for non-participation at each stage	8-9
		(c) Consider use of a flow diagram	/
Descriptive data	14*	(a) Give characteristics of study participants (eg demographic, clinical, social) and information on exposures and potential confounders	8-9
		(b) Indicate number of participants with missing data for each variable of interest	8
Outcome data	15*	Report numbers of outcome events or summary measures	9
Main results	16	(*a*) Give unadjusted estimates and, if applicable, confounder-adjusted estimates and their precision (eg, 95% confidence interval). Make clear which confounders were adjusted for and why they were included	9-10

For peer review only - http://bmjopen.bmj.com/site/about/guidelines.xhtml

		(*b*) Report category boundaries when continuous variables were categorized	/
		(*c*) If relevant, consider translating estimates of relative risk into absolute risk for a meaningful time period	/
Other analyses	17	Report other analyses done—eg analyses of subgroups and interactions, and sensitivity analyses	8
**Discussion**			
Key results	18	Summarise key results with reference to study objectives	11
Limitations	19	Discuss limitations of the study, taking into account sources of potential bias or imprecision. Discuss both direction and magnitude of any potential bias	13
Interpretation	20	Give a cautious overall interpretation of results considering objectives, limitations, multiplicity of analyses, results from similar studies, and other relevant evidence	13
Generalisability	21	Discuss the generalisability (external validity) of the study results	12
**Other information**			
Funding	22	Give the source of funding and the role of the funders for the present study and, if applicable, for the original study on which the present article is based	14

*Give information separately for exposed and unexposed groups.

Note: An Explanation and Elaboration article discusses each checklist item and gives methodological background and published examples of transparent reporting. The STROBE checklist is best used in conjunction with this article (freely available on the Web sites of PLoS Medicine at http://www.plosmedicine.org/, Annals of Internal Medicine at http://www.annals.org/, and Epidemiology at http://www.epidem.com/). Information on the STROBE Initiative is available at www.strobe-statement.org.

For peer review only

BMJ Open

Prevalence and correlates of depressive symptoms among gay, bisexual and other men who have sex with men in the PROUD randomized clinical trial of HIV pre-exposure prophylaxis

Journal:	*BMJ Open*
Manuscript ID	bmjopen-2019-031085.R1
Article Type:	Original research
Date Submitted by the Author:	23-Oct-2019
Complete List of Authors:	Miltz, Ada; University College London<br>Lampe, F; University College London<br>McCormack, Sheena; University College London, MRC CTU<br>Dunn, David; University College London, MRC CTU<br>White, Ellen; University College London, MRC CTU<br>Rodger, Alison; University College London<br>Phillips, Andrew; University College London<br>Sherr, Lorraine; University College London<br>Sullivan, Ann ; Chelsea and Westminster Healthcare NHS Trust<br>Reeves, Iain; Homerton University Hospital NHS Trust, Sexual Health<br>Clarke, Amanda; Elton John Centre, Sussex House<br>Gafos, Mitzy; London School of Hygiene and Tropical Medicine
**Primary Subject Heading**:	Epidemiology
Secondary Subject Heading:	Mental health, Public health
Keywords:	MENTAL HEALTH, EPIDEMIOLOGY, GENITOURINARY MEDICINE, PUBLIC HEALTH

SCHOLARONE™ Manuscripts

Prevalence and correlates of depressive symptoms among gay, bisexual and other men who have sex with men in the PROUD randomized clinical trial of HIV pre-exposure prophylaxis

Ada Miltz[1*], Fiona C Lampe[1], Sheena McCormack[2], David Dunn[2], Ellen White[2], Alison Rodger[1], Andrew Phillips[1], Lorraine Sherr[1], Ann K Sullivan[3], Iain Reeves[4], Amanda Clarke[5], Mitzy Gafos[6]

*Corresponding author, email: Ada.Miltz.11@ucl.ac.uk, telephone number: +44 20801 68058

Author affiliations

[1]Institute for Global Health, University College London, London, UK

[2] MRC Clinical Trials Unit, University College London, London, UK

[3] Chelsea and Westminster Hospital NHS Trust, London, UK

[4] Homerton Hospital NHS Trust, London, UK

[5] Brighton & Sussex University Hospital NHS Trust, Brighton & Sussex Medical School, Brighton, UK

[6] Department of Global Health and Development, London School of Hygiene and Tropical Medicine, London, UK

For peer review only

For peer review only - http://bmjopen.bmj.com/site/about/guidelines.xhtml

Abstract

Objectives: The aim of this analysis is to: (i) assess the prevalence of clinically significant depressive symptoms at baseline and follow-up for participants in the PROUD trial of HIV pre-exposure prophylaxis (PrEP), examining changes in prevalence over time and (ii) investigate the association of socio-economic and psychosocial factors with depression.

Methods: PROUD was an open label randomised trial evaluating the benefit of PrEP for 544 HIV-negative gay, bisexual, and other men who have sex with men (GBMSM) in England. Enrolment was between 2012 and 2014, with at least two years follow-up. Prevalence of depression (score≥10 on PHQ-9, Patient Health Questionnaire) was assessed and compared across time-points (using McNemar's χ^2 tests) and between trial arms (using χ^2 tests). Cross-sectional associations with socio-economic and psychosocial factors were examined using baseline data in modified Poisson regression models and combined 12- and 24-month follow-up data in generalized estimating equations (GEEs). Prevalence ratios were presented unadjusted and adjusted (aPR) for age, UK birth, sexual identity, university education, London study clinic site, and calendar time (and follow-up time-point in GEEs).

Results: Depression increased significantly from baseline (9.1%; 49/540) to the 12-month (14.4%; 59/410) and 24-month (14.4%; 48/333) follow-ups, possibly explained by underreporting at baseline. The prevalence of depression did not differ by study trial arm, at any time-point. In the baseline analysis, younger age, unemployment, and crystal methamphetamine use, was associated with depression. In combined analysis of 12- and 24-month data, measures of intimate partner violence (IPV) (lifetime IPV victimization aPR 2.57 [95% CI: 1.71, 3.86]), internalized homophobia (aPR 1.91 [95% CI: 1.29, 2.83]), and concealment of sexual identity (aPR 1.75 [95% CI: 1.16, 2.65]), were strongly associated with depression.

Conclusions: There is a high concomitant burden of psychosocial factors with depression among GBMSM.

Trial registration: ISRCTN (ISRCTN94465371) and ClinicalTrials.gov (NCT02065986).

For peer review only - http://bmjopen.bmj.com/site/about/guidelines.xhtml

Strengths and limitations of this study

- This is the first UK study to collect data on depression prevalence over time among gay, bisexual and other men who have sex with men (GBMSM).
- This is the first UK study to investigate associations between a range of psychosocial factors and depression among GBMSM.
- Since the data were analysed cross-sectionally, the direction of associations may be unclear for some factors.
- This study may have lacked power to detect the presence of some associations.
- There is a possibility of unmeasured confounding in this study, as information on childhood sexual abuse, income, and levels of social support was not collected.

Introduction

There is consistent evidence that depression prevalence is elevated among gay and bisexual men compared to their heterosexual counterparts in high-income countries [1]. Two theoretical models have put forward explanations for the elevated prevalence observed among sexual minorities: minority stress theory and syndemic theory. Minority stress theory describes the psychosocial consequences of being in continual conflict with a discriminatory and heteronormative social environment. Perpetual negative feedback from others is thought to lead to a process of self-stigmatization termed internalized homophobia, whereby antigay social values/attitudes are directed towards the self. Internalized homophobia often results in deep conflict and poor self-regard with negative consequences such as pervasive expectations of rejection in one's life and concealment of one's sexual identity. Concealment adds to mental distress by disallowing individuals to affiliate with people of the same sexual identity [2]. Two U.S. studies of gay, bisexual and other men who have sex with men (GBMSM) found strong associations between perceived discrimination on the basis of sexual orientation and depressive symptoms [3 4]. Internalized homophobia may explain this association. Syndemic theory proposes that concomitant widespread use of recreational drugs and experiences of intimate partner violence (IPV) among sexual minority men may be to blame for elevated levels of depression, as causal, and bidirectional, relationships are postulated between drug use, IPV, and depression. The synergistic interaction of these co-occurring factors is thought to result in an exaggerated risk of poor health outcomes [5]. A number of U.S. studies have found strong associations of recreational drug use [3 6 7] and IPV [8] with depressive symptoms. Recently, studies have also found a link between chemsex and depression [9-11]. Chemsex is a cultural phenomenon among a sub-group of gay identified men, which was first described in the UK [12]. It is the intentional use of

For peer review only - http://bmjopen.bmj.com/site/about/guidelines.xhtml

psychoactive substances (usually one or more of mephedrone, gamma-hydroxybutrate/gamma-butyrolactone [GHB/GBL] and methamphetamine) to stimulate sexual arousal, facilitate different sexual practices, and prolong sexual episodes.

A number of UK studies have examined the prevalence of, and factors associated with, depressive symptoms among GBMSM [13-18]. In several of these studies however, power to assess associations was limited due to small sample size (<200 GBMSM) [13 15 16], and one limitation of the larger studies is that data were not collected on possible psychosocial risk factors (internalized homophobia and IPV) [14 17 18]. There is a need to better understand correlates of depression among GBMSM in the UK.

This analysis uses data from the PROUD randomised clinical trial, which evaluated the impact of pre-exposure prophylaxis (PrEP) on HIV incidence, and collected data on a range of psychosocial factors. The aim is to: (i) present the prevalence of depressive symptoms at baseline and follow-up, examining changes in prevalence over time and (ii) drawing on minority stress theory and syndemic theory, investigate the association of demographic, socio-economic and psychosocial factors with depressive symptoms.

Methods

PROUD trial

The PROUD trial was a multi-centre, pragmatic open label randomised trial evaluating the benefit of PrEP as part of a package of HIV risk reduction interventions for HIV-negative GBMSM and trans women [19]. Only three trans women enrolled in PROUD, and therefore data cannot be presented separately for trans women. The study was reviewed and approved by London Bridge Research Ethics Committee (12/LO/1289).

Routine attenders at 13 genitourinary medicine (GUM) clinics in England were enrolled between November 2012 and April 2014. Volunteers were eligible if they met the following criteria: male sex at birth; aged 18 years or over; HIV-negative on the day of enrolment or in the past four weeks; reported condomless anal sex (CAS) in the past three months; and expected to have CAS again in the next three months [19]. Volunteers were randomised 1:1 to an immediate start of daily oral PrEP or a deferred start after one year. However, during follow-up, an unexpectedly high incidence of HIV was observed in the deferral arm (9.0 per 100 person-years, 90% CI: 6.1, 12.8), which led to the decision

For peer review only - http://bmjopen.bmj.com/site/about/guidelines.xhtml

by the trial steering committee in October 2014 to offer all participants PrEP. Participants had the opportunity to remain in follow-up for at least two years.

Patient and Public Involvement

The PROUD pilot study team used a number of mechanisms to promote effective and appropriate participant engagement in the research project (http://www.proud.mrc.ac.uk/about/patient-and-public-involvement-ppi/). A number of meetings or discussion forums were hosted throughout the course of the study, in which participants were actively involved in the research process. Participants were informed of these forums via the study team, advertisements in the clinics, or the study website. The types of topics participants were involved in included: (i) development of the trial protocol, including questionnaires, participant information sheets and consent forms, (ii) assessing and improving study procedures, recruitment and retention strategies, (iii) assessing and improving public involvement and communication plans, and (iv) development of plans for result dissemination and post-study access to study product. Patient and public involvement activities with a small group of PROUD participants (N=13), in the form of a short qualitative survey, was also undertaken as part of this depression project; responses are considered in the discussion.

Hypothesized relationships and selection of variables

Causal relationships with depressive symptoms were hypothesized for socio-economic factors (younger age, lower levels of educational attainment, unemployment, and having no ongoing relationship) and psychosocial measures (IPV, early age at anal sex debut [a possible marker of childhood sexual abuse], recreational drug use, alcohol use, internalized homophobia, and concealment of sexual identity) collected in PROUD. Hypotheses were based on minority stress theory and syndemic theory as well as observed relationships in previous studies, although such relationships have not been proved to be causal.

Measures

Depressive symptoms

The Patient Health Questionnaire (PHQ-9) was used to measure the prevalence of depressive symptoms at baseline and on an approximately annual basis (month-12 and 24). In the PHQ-9, participants are asked to rate how often over the past two weeks they have experienced nine specific problems (anhedonia, feeling hopeless, trouble sleeping, feeling tired, appetite problems, feeling like a failure, trouble concentrating, being slow/restless, and suicidal ideation/self-harm) [20].

For peer review only - http://bmjopen.bmj.com/site/about/guidelines.xhtml

Response options range from 'not at all' (coded as 0) to 'nearly every day' (coded as 3). In this paper, a missing response was coded as 0. Summing responses from each question generates a depressive symptom score. A cut-off point of 10 or greater for the total score indicates a possible clinically significant depressive condition [20]. In a U.S. validation study of primary care and obstetrics/ gynaecology patients, the vast majority of patients (93%) with no depressive disorder diagnosed by a (blinded) mental health professional had a PHQ-9 score less than 10, while 88% with a major depression diagnosis had scores of 10 or greater [20].

The PROUD study team reported that part way through the trial enrolment process a change in PHQ-9 reporting policy was instated, which continued to be utilized throughout follow-up. From April 2013, participants were informed that after self-completing the questionnaire they would be asked to disclose their response to the suicidal/self-harm ideation question on the PHQ-9 ('Thoughts that you would be better off dead, or of hurting yourself in some way') to trial clinical staff. The Trial Steering Committee recommended this change in reporting policy as a result of one man attempting to take his life during the course of the study.

At the baseline visit men were asked by study staff to note down any medication they were taking. All men who specified that they were taking a medication that has been licensed for treatment of depression were identified.

Intimate partner violence

At month-12 and 24, participants were asked five questions about IPV victimization and five questions about IPV perpetration, based on the 'Health and Relationships survey' [18]. Each question had the following four response options: never, more than 1 year ago, within the last year with former partner, and within the last year with current partner. The five IPV victimization questions included the following: 'Have you ever felt frightened of the behaviour of a partner', 'Have you ever needed to ask a partner's permission to work, go shopping, visit relatives, or visit friends (i.e. beyond the usual of being considerate to and checking with a partner)', 'Have you ever been hit, slapped, kicked or otherwise physically hurt by a partner', 'Have you ever been forced to have sex or made to engage in some sexual activity when you did not want to', and 'Have you ever been forced to have sex without a condom when you did not want to'. The five IPV perpetration questions were identical but asked whether the participant had perpetrated these behaviours. A positive response to any one of the five respective questions was considered to indicate lifetime IPV victimization or lifetime IPV perpetration. A missing response was included in the 'no IPV' category.

For peer review only - http://bmjopen.bmj.com/site/about/guidelines.xhtml

Age at first anal sex

Data on age at anal sex debut with a male were collected at month-12 and 24. Reporting anal intercourse at: (i) 12 years or younger and (ii) 15 years or younger, was investigated. Measure (i) may include experiences of childhood sexual abuse (CSA), however, since the age of the partner and the nature of the encounter was not known, this measure may not reflect or identify all sexual abuse experienced.

Recreational drug use and alcohol use

At baseline, participants were asked to report whether they had used recreational drugs in the past three months. Chemsex drug use was defined as use of mephedrone, GHB/GBL, and/or methamphetamine in the past three months. It is of note that men were not asked in PROUD whether these drugs had been taken before or during sex. At the 12-month and 24-month questionnaires, participants were asked whether, in the past three months, they had engaged in sex after using recreational drugs, which may also be a proxy measure for chemsex.

At baseline, a measure of higher risk alcohol consumption was investigated based on the first two questions of the WHO AUDIT-C questionnaire [21]. Participants were asked about the frequency of their alcohol consumption in the past three months, with response options: never (coded as 0), monthly or less (codes as 1), 2 or 3 times a month (coded as 2), once or twice a week (coded as 3), 3 or more times a week (coded as 4). Participants were also asked about their usual alcohol intake in units on a typical day when they are drinking, with responses 1-2 units (coded as 0), 3-4 (coded as 1), 5-6 (coded as 2), 7-9 (coded as 3), and 10+ (coded as 4). Summing responses from both questions resulted in an alcohol use score ranging from 0 to 8. A score of ≥6 was considered to indicate higher risk alcohol consumption. A missing response was included in the lower risk/no alcohol consumption category (score of <6).

Internalized homophobia and concealment of sexual identity

At month-12 and 24, participants were asked eight questions surrounding attitudes towards gay sexuality, based on the 26-item Internalized Homophobia Scale (four scales with internal reliabilities of 0.85, 0.69, 0.64, and 0.62) [22]. Each question had the following response options: strongly agree, agree, neutral or uncertain, disagree, and strongly disagree. The eight questions included the following: (i) 'I feel comfortable in gay bars', (ii) 'I feel comfortable being seen in public with an obviously transgender/gay person', (iii) 'I feel comfortable discussing homosexuality in a public situation', (iv)'I feel comfortable being a transgender/gay man', (v) 'Homosexuality is morally

For peer review only - http://bmjopen.bmj.com/site/about/guidelines.xhtml

acceptable to me', (vi) 'Even if I could change my sexual orientation I wouldn't', (vii) 'Obviously effeminate homosexual men make me feel uncomfortable' and (viii) 'Social situations with transgender/gay men make me feel uncomfortable'. Responding with 'strongly agree' or 'agree' to questions (vii) or (viii), or with 'strongly disagree' or 'disagree' to questions (i), (ii), (iii), (iv), (v), or (vi) was considered to indicate internalized homophobia.

At month-12 and 24, participants were also asked how many of their (i) work colleagues, (ii) friends, and (iii) close family, know that they are gay/transgender/have sex with men. Response options for groups (i)-(iii) included: all/almost all, >half, <half, few, and none. Participants who did not report being 'out' to all/almost all friends, work colleagues and close family were considered to indicate concealment of their sexual identity.

Statistical analysis

Prevalence of depressive symptoms (PHQ-9≥10) was compared between trial arms at each time-point using χ^2 tests and across time-points (trial arms combined) using McNemar's χ^2 test to account for paired responses.

Unadjusted and adjusted associations of demographic, socio-economic, and psychosocial (drinking and drug use) factors with depressive symptoms (PHQ-9≥10) at baseline were assessed using modified Poisson regression with a robust variance estimator, in order to produce prevalence ratios (PRs) [23]. Seven socio-demographic factors assumed not to be on hypothesized causal pathways were adjusted for: Age group (<25, 25-29, 30-39, or 40+ years), born in the UK (yes or no/missing), sexual identity (gay or bisexual/straight), university education (yes or no/missing), ongoing relationship (yes or no/missing), London study clinic site (yes or no), and calendar time (year and calendar quarter). For each factor investigated, separate socio-demographic adjusted models were constructed.

Generalized estimating equations (GEEs) were used to assess the cross-sectional association of psychosocial factors (sexualized drug use, age at anal sex debut, IPV, internalized homophobia, 'outness') with depressive symptoms, including data from the 12 and 24-month questionnaires. GEEs were used to account for multiple responses from individual participants, and were fitted using Poisson models with a log link and compound symmetry for the correlational structure. In the GEEs, adjustment was made for the same set of baseline socio-demographic factors described above; with the exceptions that age and calendar time were included as time-updated variables, baseline 'ongoing relationship' was not adjusted for as this status may change over time, and additional

For peer review only - http://bmjopen.bmj.com/site/about/guidelines.xhtml

adjustment was made for follow-up time-point (month-12, month-24). Use of GEE models in this way represents a pooled cross-sectional analysis. A longitudinal analysis was not feasible as the number of 'incident' cases of PHQ-9 depression was low; of the 233 men who appeared to have no history of depression based on PROUD data (PHQ-9<10 at baseline and month-12 and no antidepressant use, as captured at baseline only), 22 reported clinically significant symptoms (PHQ-9≥10) at month-24.

For each measure and at each time-point, the proportion of missing responses was <5%. For psychosocial measures (depression, drinking, drug use, sexualized drug use, internalized homophobia, and IPV), missing responses were considered to indicate that the event did not occur. There appeared to be a common response pattern in which only those experiences that had occurred were ticked on the questionnaire. Given the very low proportion of missing responses, missing values were considered to indicate a lower level of educational attainment (i.e. no university degree), no ongoing relationship, and no current employment. A sensitivity analysis was undertaken excluding missing values when defining each variable. The findings were very similar to the main analysis. All analyses were performed in STATA statistical software (version 13) [24] and reported according to the STROBE guidelines.

Results

There were 544 participants enrolled in PROUD. In total, 540 completed a baseline questionnaire, of whom 410 (76%) completed a 12-month questionnaire, and 333 (62%) completed a 24-month questionnaire. Overall, 96% of men identified as gay and 3% as bisexual (includes three trans women). Eighty-two percent of men were of white ethnicity; median age was 35 years (Interquartile range [IQR]: 29-42 years) at baseline (Table 1). The prevalence of psychosocial measures collected at baseline and follow-up time-points are presented in Tables 1 and 2. Briefly, lifetime IPV was reported by 45% of participants for victimization and 20% for perpetration at month-12, and 42% of participants indicated internalized homophobia at month-12. The prevalence of CAS with ≥2 partners in the past three months was: 76% at baseline, 74% at month-12, and 75% at month-24.

Prevalence of depressive symptoms across trial arms and time-points

The prevalence of depressive symptoms was 9.1% (49/540) at baseline, 14.4% (59/410) at month-12, and 14.4% (48/333) at month-24. In terms of changes in individual depression status between time-points, among the 410 men who completed the baseline and 12-month questionnaire, 38 men

For peer review only - http://bmjopen.bmj.com/site/about/guidelines.xhtml

For peer review only

reported depressive symptoms (PHQ-9≥10) at baseline, 22 (57.9%) of whom continued to report this measure of depression at month-12. Of the 372 men who did not report depressive symptoms at baseline, 37 (10.0%) reported it at month-12. Among the 333 men who completed the baseline and 24-month questionnaire, 32 men who reported depressive symptoms (PHQ-9≥10) at baseline, 11 (34.4%) of whom continued to report this measure of depression at month-24. Of the 301 men who did not report depressive symptoms at baseline, 37 (12.3%) reported it at month-24. Finally, among the 307 men who completed both the 12- and 24-month questionnaire, of 43 men who reported depressive symptoms (PHQ-9≥10) at month-12, 16 (37.2%) continued to report this measure of depression at month-24. Of the 264 men who did not report depression at month-12, 29 (11.0%) reported it at month-24.

The prevalence of depressive symptoms was slightly higher in the immediate than the deferred PrEP group at baseline (10.6% versus 7.5%) and at subsequent time-points (16.7% versus 11.9% at month-12; 16.5% versus 11.9% at month-24). Differences between randomised groups were not statistically significant at any time-point (p≥0.2). The within-person increase in prevalence from 9.3% at baseline to 14.4% at month-12 (among 410 men) and from 9.6% at baseline to 14.4% at month-24 (among 333 men) was significant (p≤0.04), but there was no difference from 14.0% at month-12 to 14.7% at month-24 (among 307 men).

Overall, 8.5% (46/540) of men reported antidepressant use at baseline; 20.4% (n=10) of men with depressive symptoms (PHQ-9≥10) and 7.3% (n=36) of men without. There were 85 men (15.7%) who either had depressive symptoms (PHQ-9≥10) or reported antidepressant use, of whom 54.1% (n=46) were receiving antidepressant medication.

Association of socio-economic and psychosocial (drinking and drug use) factors with depressive symptoms (PHQ-9≥10) at baseline

Younger age, unemployment, methamphetamine use in the past three months, and completion of baseline questionnaire before the first quarter of 2013 was associated with higher prevalence of depressive symptoms in unadjusted and adjusted analysis (see Tables 3 and 4).

Association of psychosocial factors (sexualized drug use, age at sex debut, IPV, internalized homophobia, 'outness') with depressive symptoms at the 12- and 24-month questionnaire

The prevalence of depressive symptoms was two to three-fold higher among men who reported: ever having been a victim of IPV, IPV victimization in the past year, ever having perpetrated IPV,

For peer review only - http://bmjopen.bmj.com/site/about/guidelines.xhtml

perpetration of IPV in the past year, internalized homophobia, and concealment of sexual identity, in unadjusted and adjusted analysis (see Table 5).

Exploration of changes in depression prevalence

The baseline prevalence of suicidal ideation on PHQ-9 was significantly higher for men who completed their questionnaire before April 1st 2013 (n=71) compared to men whose completion was on or after April 1st 2013, from which time they were asked to disclose their response (n=469) (19.7% vs. 9.6%; Pearson χ^2 test p=0.011). Similarly, the baseline prevalence of depressive symptoms (PHQ-9≥10) was significantly higher among men who completed their questionnaire before versus on/after April 1st 2013 (21.1% vs. 7.3%; Pearson χ^2 test p<0.001). A possible calendar time effect can also be seen in Table 3, and this relationship remained after adjusting for socio-demographics. There was evidence of an increase in depression from baseline to month-12 for the 353 men with baseline completion on/after April 1st 2013 and 12-month data (7.9% vs. 14.7%), but not for the 57 men with baseline completion before April 1st 2013 and 12-month data (17.5% vs. 12.3%; McNemar's χ^2 test p-values 0.366 and <0.001 respectively). When investigating the modifying effect of baseline completion date on the association between follow-up time-point and depressive symptoms in GEEs, the interaction p-value was 0.004: for month-12 vs. baseline, PR 0.61 95% CI: 0.28, 1.34 for the time period before April 1st 2013 and PR 1.97 95% CI: 1.41, 2.75 for the time period on/after April 1st 2013 respectively.

Discussion

In the PROUD trial of GBMSM reporting very high levels of CAS partners, the prevalence of depressive symptoms by PHQ-9 ranged from 9.1% at baseline to 14.4% at months 12 and 24. Younger men, those who were not employed, those who reported methamphetamine use, IPV, internalized homophobia, and concealment of sexual identity, were more likely to report depressive symptoms. Depression prevalence did not differ significantly between trial arms at any time-point, which was in line with that reported in the iPrEX (placebo controlled) trial of PrEP [25].

The link between psychosocial factors, including depressive symptoms, and sexual risk behaviour among GBMSM in PROUD has been addressed in a separate paper [26]. Unlike in previous studies [16 17], no link was found between depression and CAS. Given the PROUD inclusion criteria, all participants reported recent CAS partners. It may be that symptoms of depression do not distinguish between men reporting higher levels of CAS partners and lower levels of CAS partners.

For peer review only - http://bmjopen.bmj.com/site/about/guidelines.xhtml

In the current study, findings suggest that some men may have underreported depressive symptoms at baseline after April 2013. It is possible that newly enrolled participants may have been worried about their inclusion in the trial if they disclosed thoughts of suicide or self-harm to the investigator, and that such concerns may have dissipated over time once participation in the study was established.

Patient and public involvement activities with a small group of PROUD participants (N=13), in the form of a short qualitative survey, provided some participant perspectives on the change in depressive symptoms over time. Some highlighted the possibility that greater familiarity with the questionnaire and study staff over time may have resulted in increased reporting of depressive symptoms at follow-up visits. Additionally, partaking in a clinical trial was thought to be one of the processes that helps individuals recognize/acknowledge their own depression in an environment where access to support is provided. Other suggested explanations for an actual increase in depressive symptoms included the possibility of greater socio-economic hardship in a period of national financial instability, risk taking behaviours in relation to CAS and chemsex, or PrEP-related stigma. None of the 13 participants specifically theorized that engagement in chemsex increased over the study, but chemsex was a common feature in discourse on depression. Although these participants were informed of the lack of difference between the immediate and deferred PrEP groups with regards to depression prevalence, some men cited that PrEP related side-effects and stigma, as well as fear of loss of access to PrEP after study completion, may have increased over time.

The depression prevalence observed in PROUD at month-12 (14.4%) and 24 (14.4%) is comparable to that found in the AURAH cross-sectional survey of HIV-negative GBMSM attending GUM services in England, 2013-2014 (PHQ-9≥10): 12.4% (n=166/1340) and 15.0% (n=122/815) among GBMSM reporting recent sex (with or without a condom) and recent CAS respectively [17]. Depression prevalence at annual follow-up visits in PROUD was also broadly similar to other studies of GBMSM in the UK [13-18], although comparison is made difficult due to different study settings and measures of depression, and the fact that people with HIV were included in most studies (Textbox 1). The prevalence of depressive symptoms observed at follow-up in PROUD was almost twice that observed in a random sample of men and women in England (2007/2008); 7% reported PHQ-9≥10 [27], although socio-demographic differences may confound this comparison.

For peer review only - http://bmjopen.bmj.com/site/about/guidelines.xhtml

Although most studies based prevalence estimates on depressive symptoms only, the proportion of participants with evidence of current depression is higher when taking into account treatment for depression as well as symptoms. In the PROUD trial, 36 (78%) of the 46 men reporting antidepressant use at baseline were not positive for PHQ-9 depressive symptoms, and the prevalence of depression when considering symptoms or treatment was 15.7%.

In recent European studies, younger age, markers of lower socio-economic status, lower levels of a supportive network, reporting a bisexual identity [14 17 28], and recreational drug use [17 28], appear to be important factors for depression in the GBMSM literature. Mephedrone and crystal methamphetamine use have also been linked to acute symptoms of depression in a qualitative study of 30 gay men in London [9]. Some men reported a sense of loss of intimacy with a sexual partner when engaging in chemsex. There is also some evidence to suggest that group sex environments may leave some individuals vulnerable to mistreatment particularly if drugs are used, given their impact on inhibition and self-regulation [29]. Chemsex associated drug use has been found to be strongly associated with depressive symptoms among UK GBMSM in the AURAH study, including after adjustment for socio-demographic factors [17]. Although the pattern of associations was similar in the PROUD trial, some of the differences observed (i.e. between sexual identities, number of recreational drugs used, and any chemsex drug use) were not statistically significant. It may be that due to the possible underreporting of depression at baseline and the limited sample size of this trial, differences that may exist were not detected. It is also important to note that this sample of men is unique - they all reported very high levels of CAS partners and agreed to participate in an RCT. Therefore, associations may differ from other samples of GBMSM.

There is some evidence to suggest that CSA is associated with depression among GBMSM in the U.S. [30]. In the PROUD trial, a very young age at anal sex debut was not associated with depression. This measure may be a poor proxy for CSA as it is not likely to reflect or pick up all sexual abuse experienced. Strong associations were found of any IPV victimization and any IPV perpetration with depressive symptoms in the PROUD trial. Evidence from a recent meta-analysis of mostly U.S. studies suggests that exposure to any kind of IPV victimization is associated with increased odds of depressive symptoms (pooled OR 1.52 95% CI: 1.24, 1.86) [8]. Similar findings were observed in six recent studies [5 18 31-34]. In one UK study (GUM clinic attendees, 2010-2011), depression (HADS≥8) was significantly elevated among men reporting IPV perpetration in the past year (20.7% vs. 11.5%), but the association was attenuated after adjusting for socio-economic factors (OR 3.7 95% CI: 1.0, 14.6; *p=0.060*) [18]. Adjusting for income may in part have contributed to the attenuation. Financial

For peer review only - http://bmjopen.bmj.com/site/about/guidelines.xhtml

insecurity may be closely linked to depression, and financial inequalities within intimate partnerships may increase the risk of controlling and abusive behaviours. It was not possible to investigate the confounding effect of income/financial security in PROUD.

As described by syndemic theory, recreational drug use, IPV and depression may influence each other in a cyclical pattern, with implications for an exaggerated risk of poor health outcomes [5]. IPV may distort one's perceived self-worth and capacity to influence important outcomes in one's life, which may lead to depressive symptoms [35], in turn depression may heighten one's vulnerability to dysfunctional relationship dynamics as adaptive coping mechanisms are distorted [36-38]. Recreational drug use may cause symptoms of depression, which in turn results in further use of drugs to induce a state of cognitive release and escape from symptoms.

In line with minority stress theory, a measure of internalized homophobia and concealment of sexual identity was strongly associated with depressive symptoms in the PROUD trial. Due to the significance of early life socialization experiences, the minority stress model suggests that residual internalized homophobia may be a permanent fixture throughout the life-course for a sexual minority individual. If individuals integrate a high degree of antigay attitudes into their perception of self, depression may follow [2]. This may in part be the result of constant preoccupation with, and cognitive effort involved in, hiding one's identity [39], which is a profoundly heavy burden to carry. Three factors are hypothesized to reverse the process of internalized homophobia and reduce the risk of mental health distress: (i) family support, (ii) personal resources including personality characteristics, and (iii) group resources/social structural factors including community solidarity and cohesiveness. Very few studies have collected this information. There is a need to better understand correlates of these protective factors, as this may be useful for informing interventions to reduce the burden of depression among sexual minority men.

Strengths and limitations

This is the first UK study to collect data on depression prevalence over time among GBMSM, and investigate associations with a range of psychosocial factors. A change in methodology part way through baseline data collection may have affected the reporting of depressive symptoms, limiting the validity of findings at baseline. Given the relatively small sample size of the PROUD trial, the examination of factors associated with depression was based on cross-sectional analysis, utilising all available observations. Some participants were not included in the GEE analysis of psychosocial correlates due to missing questionnaires at months 12 and 24. However, men with and without

For peer review only - http://bmjopen.bmj.com/site/about/guidelines.xhtml

depressive symptoms (PHQ-9≥10) at baseline had similar proportions completing the 12-month questionnaire (22.5% vs. 24.2%; χ^2 test p=0.780) and those with and without depressive symptoms at the 12-month questionnaire had similar proportions completing the 24-month questionnaire (27.1% vs. 24.8%; χ^2 test p=0.702). When stratifying by trial arm, there remained no difference between men with and without depressive symptoms in terms of proportion completing follow-up questionnaires. Even after including repeated observations in GEE models, the analysis may have lacked power to accurately detect the presence of some associations. Furthermore, the direction of any causality may be unclear for some associations. For this reason, as well as the possibility of unmeasured confounders, inferences regarding causal factors for depression are limited. Although chemsex drug use was investigated in this study, the cultural phenomenon of chemsex was not explicitly measured, as participants were not asked whether these drugs were taken to enhance sexual experiences.

There have been no longitudinal studies that are designed and powered to investigate depression among GBMSM. Future longitudinal analyses would be useful in investigating the suggested causal pathways in this paper. Such a study would require careful consideration in how to measure incident depression, as new symptoms may indicate first episode or recurrent depression. Therefore, capturing data on previous diagnoses and treatment is important. Following participants from a young age is likely to yield the most accurate data with regards to depression incidence. Finally, there is a need to examine associations with measures of family and community support, income, and abuse in childhood, as well as the role of these factors in relationships investigated in this paper.

Conclusions and implications

In the context of interviewer administered surveys or surveys in which interviewers/study staff are made aware of individual responses, participants may be reluctant to disclose information on depressive symptomatology. This may be particularly true in clinical trial settings that are subject to strict inclusion/exclusion criteria, as eligible participants may perceive the disclosure of current depressive symptoms as prohibiting study inclusion. Researchers should be aware of the potential limitations to reporting depression prevalence under these circumstances.

Our findings add to the growing body of research in high-income countries suggesting a high concomitant burden of sexual minority related stress, substance use, and IPV with depression among GBMSM. Since these psychosocial measures are relevant to GBMSM at high-risk of HIV and seeking PrEP, it is recommended that training on awareness and enquiry about psychosocial issues

For peer review only - http://bmjopen.bmj.com/site/about/guidelines.xhtml

should be enhanced in sexual health services. This should include referral to substance use services and culturally appropriate structures set up to address IPV among same-sex male couples. Furthermore, findings suggest that new interventions addressing sexual minority related stresses are needed in order to foster greater community affiliation and support, and curtail stress. Intervening with sexual minority youths in schools and/or in LGBT+ youth groups may prevent the internalizing of homophobic attitudes and the onset of mental health problems. Although it is important to be cognizant of the unique forms of stress experienced by sexual minority men, findings from PROUD also emphasise the link between socio-economic disadvantage and poor mental health. Therefore, emphasis on alleviating economic hardship together with efforts to promote self-acceptance and pride towards one's sexual orientation is recommended when addressing depression among sexual minority men.

In many countries, sexual minorities are being accepted and celebrated more so than ever in history [40-43]. This social climate provides a solid platform from which to launch extensive efforts to eliminate homophobic discrimination in society at large. Such a structural change is likely to be profoundly beneficial to the health and wellbeing of sexual minority people. Efforts may include national educational campaigns and specialised training for health care providers on homophobia and heterosexism.

Ethics approval and consent to participate

The study was reviewed and approved by London Bridge Research Ethics Committee (12/LO/1289). Written informed consent was obtained from all participants. The study protocol is available online (http://www.proud.mrc.ac.uk/about/study-protocol/).

Data Sharing Statement

The PROUD data is held at MRC CTU at UCL, which encourages optimal use of data by employing a controlled access approach to data sharing, incorporating a transparent and robust system to review requests and provide secure data access consistent with the relevant ethics committee approvals. All requests for data are considered and can be initiated by contacting proud.mrcctu@ucl.ac.uk.

Competing interests

The PROUD study was provided drug free of charge by Gilead Sciences plc. which also distributed it to participating clinics and provided funds for additional diagnostic tests for HCV and drug levels. AP has received payments for presentations made at meetings sponsored by Gilead in spring 2015. EW

For peer review only - http://bmjopen.bmj.com/site/about/guidelines.xhtml

has had tuition fees and a stipend paid by Gilead. AC received advisory board fees from Gilead Sciences plc. and GSK/ViiV; speaker fees from Gilead and conferences bursaries from Gilead & Janssen. SM reports grants from the European Union H2020 scheme, EDCTP 2, the National Institute of Health Research, and Gilead Sciences; other support from Gilead Sciences, and the Population Council Microbicide Advisory Board; and is Chair of the Project Advisory Committee for USAID grant awarded to CONRAD to develop tenofovir-based products for use by women (non-financial).

Authors' contributions

Conceived and designed the study: MG FCL ARM SM DD EW AR AP LS AC AKS IR. Analysed the data: ARM. Wrote the paper: ARM MG FCL. All authors read and approved the final manuscript.

Funding

Sheena McCormack and David Dunn were supported by a Medical Research Council grant (MRC_UU_12023/23).

Acknowledgements

We thank all PROUD trial participants.

The PROUD study group:

David I. Dolling, Monica Desai, Alan McOwan, Richard Gilson, Amanda Clarke, Martin Fisher, Gabriel Schembri, Ann K. Sullivan, Nicola Mackie, Iain Reeves, Mags Portman, Vanessa Apea, John Saunders, Julie Fox, Jake Bayley, Michael Brady, Killian Quinn, Christine Bowman, Clarie Dewsnap, Charles J. Lacey, Stephen Taylor, David White, Simone Antonucci, Mitzy Gafos, Sheena McCormack, Owen N. Gill, David T. Dunn, and Anthony Nardone.

PROUD clinic teams:

Drew Clark, Paul Davis, James Hand, Machel Hunt, Rebecca Neale, Jackie O'Connell, Liat Sarner, John Saunders, Louise Terry, Angelina Twumasi, Salina Tsui, Dayan Vijeratnam, Ryan Whyte, Andy Williams, Sian Gately, Gerry Gilleran, Jill Lyons, Chris McCormack, Katy Moore, Cathy Stretton, Alex Acheampong, Michael Bramley, Marion Campbell, Ruby Chowdhry, Stewart Eastwood, Babs Fennell, Wendy Hadley, Kerry Hobbs, Sarah Kirk, Nicky Perry, Charlotte Rawlinson, Celia Richardson, Claire Richardson, Mark Roche, Emma Simpkin, Simon Shaw, Elisa Souto, Julia Williams, Elaney Youssef, Tristan Barber, Cindy Eliot, Serge Fedele, Chris Higgs, Kathryn McCormick, Alexandra Meijer, Sam Pepper, Jane Rowlands, Gurmit Singh, Alfredo SolerCarracedo, Sonali Sonecha, David Taylor, Lervina

For peer review only - http://bmjopen.bmj.com/site/about/guidelines.xhtml

For peer review only

Thomas, Frederick Attakora, Marina Bourke, Richard Castles, Rebecca Clark, Anke De-Masi, Veronica Espa, Rumbidzai Hungwe, Martin Lincoln, Sifiso Mguni, Rhianon Nevin-Dolan, Hannah Alexander, Lucy Campbell, Sophie Candfield, Shema Doshi, Olivia Liddle, Larissa Mulka, Priyanka Saigal, James Stevenson, James Boateng, Brynn Chappell, Susanna Currie, Carolyn Davies, Dornubari Lebari, Matthew Phillips, Lisa Southon, Sarah Thorpe, Anna Vas, Chris Ward, Claire Warren, Stephanie Yau , Alejandro Arenas-Pinto, Asma Ashraf, Matthew Bolton, Lewis Haddow, Sara McNamara, Ana Milinkovic, June Minton, Dianne Morris, Clare Oakland, Steve O'Farrell, Pierre Pellegrino, Sarah Pett, Nina Vora, Carmel Young, Taris Zarko-Flynn, Wilbert Ayap, Ling Jun Chen, Adam Croucher, Sarah Fidler, Kristin Kuldanek, Ken Legg, Agathe Leon, Nadia Naous, Severine Rey, Judith Zhou, Margaret-Anne Bevan, Nina Francia, Eleanor Hamlyn, Lisa Hurley, Helen Iveson, Isabelle Jendrulek, Tammy Murray, Alice Sharp, Andrew Skingsley, Chi Kai Tam, Al Teague, Caroline Thomas, Juan-Manuel Tiraboschi, Christine Brewer, Richard Evans, Jan Gravely, Gary Lamont, Fabiola Martin, Georgina Morris, Sarah Russell-Sharpe, John Wightman, Anthony Bains, Gill Bell, Terry Cox, Charlie Hughes, Hannah Loftus, Naomi Sutton, Debbie Talbot, Vince Tucker.

For peer review only - http://bmjopen.bmj.com/site/about/guidelines.xhtml

For peer review only

Table 1: Prevalence of demographic, socio-economic, and psychosocial (drinking and drug use) factors at baseline in PROUD

Measure:	Category:	Baseline [N=540] n (%)
Calendar time (year & calendar quarter [Q]) of baseline data collection	2012 Q4<br>2013 Q1<br>2013 Q2<br>2013 Q3<br>2013 Q4<br>2014 Q1<br>2014 Q2	8 (1.5%)<br>63 (11.7%)<br>85 (15.7%)<br>113 (20.9%)<br>119 (22.0%)<br>125 (23.2%)<br>27 (5.0%)
Age	<25<br>25-29<br>30-34<br>35-39<br>40-44<br>45+	54 (10.0%)<br>96 (17.8%)<br>114 (21.1%)<br>97 (18.0%)<br>81 (15.0%)<br>98 (18.2%)
Born in the UK and ethnicity	Yes, white<br>Yes, BAME<br>No, white<br>No, BAME	287 (53.4%)<br>35 (6.5%)<br>152 (28.3%)<br>64 (11.9%)
Self-reported sexual identity	Gay<br>Bisexual<br>Straight	513 (95.7%)<br>17 (3.2%)<br>6 (1.1%)
University Education	Yes<br>No/missing	327 (60.6%)<br>213 (39.4%)
Employed	Yes<br>No/missing	439 (81.3%)<br>101 (18.7%)
Ongoing relationship	Yes<br>No/missing	246 (45.6%)<br>294 (54.4%)
Study region	London<br>Outside London	375 (69.4%)<br>165 (30.6%)
Higher-risk drinking	No/missing<br>Yes	367 (68.0%)<br>173 (32.0%)
Recreational drug use (past 3 months)	0/missing<br>1<br>2-4<br>5+	148 (27.4%)<br>87 (16.1%)<br>159 (29.4%)<br>146 (27.0%)
Chemsex-associated drug use (past 3 months)	No/missing<br>Yes	309 (57.2%)<br>231 (42.8%)
Crystal meth (past 3 months)	No/missing<br>Yes	442 (81.9%)<br>98 (18.2%)
Mephedrone (past 3 months)	No/missing<br>Yes	343 (63.5%)<br>197 (36.5%)
GHB/GBL (past 3 months)	No/missing<br>Yes	371 (68.7%)<br>169 (31.3%)
Ketamine (past 3 months)	No/missing<br>Yes	451 (83.5%)<br>89 (16.5%)
Cocaine (past 3 months)	No/missing<br>Yes	401 (74.3%)<br>139 (25.7%)
Ecstasy/MDMA (past 3 months)	No/missing<br>Yes	450 (83.3%)<br>90 (16.7%)

For peer review only - http://bmjopen.bmj.com/site/about/guidelines.xhtml

Table 2: Prevalence of psychosocial factors (sexualized drug use, age at sex debut, IPV, internalized homophobia, 'outness') at follow-up time-points in PROUD

Measure:	Category:	12-month [N=410] n (%)	24-month [N=333] n (%)
Had sex after using recreational drugs (past 3 months)	No/missing<br>Yes	186 (45.4%)<br>224 (54.6%)	174 (52.3%)<br>159 (47.8%)
Age≤15 years at anal sex debut	No<br>Yes	307 (77.7%)<br>88 (22.3%)	251 (78.2%)<br>70 (21.8%)
Age<13 years at anal sex debut	No<br>Yes	373 (94.4%)<br>22 (5.6%)	302 (94.1%)<br>19 (5.9%)
Any IPV victimization	No/missing<br>Yes	226 (55.1%)<br>184 (44.9%)	199 (59.8%)<br>134 (40.2%)
Any IPV victimization in the past year	No/missing<br>Yes	346 (84.4%)<br>64 (15.6%)	284 (85.3%)<br>49 (14.7%)
Any IPV perpetration	No/missing<br>Yes	330 (80.5%)<br>80 (19.5%)	273 (82.0%)<br>60 (18.0%)
Any IPV perpetration in the past year	No/missing<br>Yes	378 (92.2%)<br>32 (7.8%)	310 (93.1%)<br>23 (6.9%)
Internalised homophobia	No/missing<br>Yes	239 (58.3%)<br>171 (41.7%)	195 (58.6%)<br>138 (41.4%)
Concealment of sexual identity	No<br>Yes	206 (51.6%)<br>193 (48.4%)	166 (50.6%)<br>162 (49.4%)

For peer review only

For peer review only - http://bmjopen.bmj.com/site/about/guidelines.xhtml

Table 3: Unadjusted and adjusted associations of demographic and socio-demographic factors with depressive symptoms at baseline in PROUD

N=540 men at baseline		Depressive symptoms (PHQ-9≥10)					
		%	*p-value*[a]	Unadjusted PR [95% CI]	Overall *p-value*[d]	Adjusted[e] PR [95% CI]	*Overall p-value*[d]
Calendar time (year & calendar quarter [Q])[f]	2012 Q4 – 2013 Q1	21.1%	*0.001*	1	*0.003*	1	*0.004*
	2013 Q2	2.4%	*0.20*[b]	0.11 [0.03, 0.47]	*0.25*[b]	0.12 [0.03, 0.47]	*0.26*[b]
	2013 Q3	7.1%		0.34 [0.15, 0.75]		0.34 [0.15, 0.76]	
	2013 Q4	8.4%		0.40 [1.19, 0.84]		0.39 [0.18, 0.83]	
	2014 Q1 – Q2	9.2%		0.44 [0.22, 0.85]		0.45 [0.23, 0.88]	
Age	<25	22.2%	*0.003*	3.63 [1.44, 9.13]	*0.006*	3.49 [1.44, 8.44]	*0.003*
	25-29	8.3%	*0.002*[b]	1.36 [0.49, 3.78]	*0.003*[b]	1.22 [0.43, 3.51]	*0.004*[b]
	30-34	12.3%		2.01 [0.80, 5.02]		1.97 [0.78, 4.97]	
	35-39	6.2%		1.01 [0.34, 3.03]		0.96 [0.31, 3.00]	
	40-44	3.7%		0.60 [0.16, 2.35]		0.60 [0.16, 2.31]	
	45+	6.1%		1		1	
Born in the UK and white ethnicity	Yes, white	9.1%	*0.32*[c]	1	*0.37*	1	*0.44*
	Yes, BAME	14.3%		1.58 [0.65, 3.84]		0.93 [0.41, 2.09]	
	No, white	6.6%		0.73 [0.36, 1.47]		0.77 [0.36, 1.65]	
	No, BAME	12.5%		1.38 [0.65, 2.91]		1.59 [0.72, 3.54]	
Self-reported sexual identity[g]	Gay	9.0%	*0.46*[c]	1	*0.50*	1	*0.64*
	Bisexual	13.0%		1.45 [0.49, 4.33]		1.26 [0.48, 3.31]	
	Straight						
University Education	Yes	8.9%	*0.84*	1	*0.84*	1	*0.94*
	No/missing	9.4%		1.06 [0.61, 1.82]		0.98 [0.54, 1.77]	
Employed	Yes	6.8%	*<0.001*	1	*<0.001*	1	*0.001*
	No/missing	18.8%		2.75 [1.62, 4.69]		2.52 [1.45, 4.37]	
Ongoing relationship	Yes	6.5%	*0.06*	1	*0.06*	1	*0.15*
	No/missing	11.2%		1.73 [0.97, 3.06]		1.50 [0.86, 2.63]	
Study region	London	9.3%	*0.75*	1	*0.75*	1	*0.66*
	Outside London	8.5%		0.91 [0.50, 1.64]		0.86 [0.44, 1.69]	

BAME= Black, Asian and minority ethnic.
[a] Pearson χ^2 test.
[b] Test for trend.
[c] Fisher's exact test.
[d] p-value by Wald test using modified Poisson regression with a robust variance estimator in order to produce adjusted prevalence ratios.

For peer review only - http://bmjopen.bmj.com/site/about/guidelines.xhtml

[e] Age (included as four categories: <25, 25-29, 30-39, 40+), born in the UK, sexual identity (gay or bisexual/straight), university education, relationship status, London study clinic site, and calendar time.
[f] Some calendar quarters (Q4 2012 & Q1 2013; Q1-Q2 2014) were combined due to small cell counts.
[g] A dichotomized version of self-reported sexual identity is investigated in analysis due to very small numbers: gay or bisexual/straight.

For peer review only

Table 4: Unadjusted and adjusted associations of psychosocial factors (drinking and drug use) with depressive symptoms at baseline in PROUD

N=540 men at baseline		Depressive symptoms (PHQ-9≥10)					
		%	*p-value*[a]	Unadjusted PR [95% CI]	Overall *p-value*[d]	Adjusted[e] PR [95% CI]	Overall *p-value*[d]
Higher-risk drinking	No/missing<br>Yes	8.5%<br>10.4%	*0.46*	1<br>1.23 [0.71, 2.14]	*0.46*	1<br>1.25 [0.68, 2.29]	*0.48*
Recreational drug use (past 3 months)	0/missing<br>1<br>2-4<br>5+	10.1%<br>4.6%<br>6.9%<br>13.0%	*0.11*<br>*0.40*[b]	1<br>0.45 [0.16, 1.32]<br>0.68 [0.32, 1.44]<br>1.28 [0.68, 2.43]	0.13<br>*0.45*[b]	1<br>0.57 [0.20, 1.64]<br>0.89 [0.39, 2.02]<br>1.34 [0.71, 2.56]	0.32<br>*0.32*[b]
Chemsex-associated drug use	No/missing<br>Yes	8.1%<br>10.4%	*0.36*	1<br>1.28 [0.75, 2.19]	*0.36*	1<br>1.31 [0.77, 2.23]	*0.33*
Crystal meth (past 3 months)	No/missing<br>Yes	7.7%<br>15.3%	*0.02*	1<br>1.99 [1.13, 3.51]	*0.02*	1<br>1.95 [1.06, 3.61]	*0.03*
Mephedrone (past 3 months)	No/missing<br>Yes	9.0%<br>9.1%	*0.97*	1<br>1.01 [0.58, 1.76]	*0.97*	1<br>0.99 [0.56, 1.73]	*0.96*
GHB/GBL (past 3 months)	No/missing<br>Yes	8.4%<br>10.7%	*0.39*	1<br>1.27 [0.73, 2.21]	*0.39*	1<br>1.23 [0.72, 2.11]	*0.46*
Ketamine (past 3 months)	No/missing<br>Yes	8.4%<br>12.4%	*0.24*	1<br>1.47 [0.78, 2.76]	*0.24*	1<br>1.43 [0.76, 2.71]	*0.27*
Cocaine (past 3 months)	No/missing<br>Yes	8.5%<br>10.8%	*0.41*	1<br>1.27 [0.72, 2.27]	*0.41*	1<br>1.14 [0.66, 1.97]	*0.64*
Ecstasy/MDMA (past 3 months)	No/missing<br>Yes	8.4%<br>12.2%	*0.26*	1<br>1.45 [0.77, 2.72]	*0.25*	1<br>1.31 [0.69, 2.49]	*0.41*

[a] Pearson χ^2 test.
[b] Test for trend.
[c] Fisher's exact test.
[d] p-value by Wald test using modified Poisson regression with a robust variance estimator in order to produce adjusted prevalence ratios.
[e] Age (included as four categories: <25, 25-29, 30-39, 40+), born in the UK, sexual identity (gay or bisexual/straight), university education, relationship status, London study clinic site, and calendar time.

For peer review only - http://bmjopen.bmj.com/site/about/guidelines.xhtml

Table 5: Unadjusted and adjusted associations of psychosocial factors (sexualized drug use, age at sex debut, IPV, internalized homophobia, 'outness') with depressive symptoms using 12- and 24-month PROUD questionnaire data in GEE models

N=436 men, observations=743 (using data from men who completed the 12-month or 24-month questionnaire in GEE models)		Depressive symptoms (PHQ-9≥10)			
		Unadjusted PR [95% CI]	Overall p-value[a]	Adjusted[b] PR [95% CI]	Overall p-value[a]
Had sex after using recreational drugs (past 3 months)<br>N=436[c], Obs=743[d] ; N=432[e], Obs=737[f]	No/missing<br>Yes	1<br>1.38 [0.93, 2.06]	*0.11*	1<br>1.38 [0.92, 2.07]	*0.12*
Age≤15 years at anal sex debut<br>N=433[c], Obs=716[d] ; N=429[e], Obs=710[f]	No<br>Yes	1<br>1.27 [0.80, 2.02]	*0.31*	1<br>1.20 [0.75, 1.94]	*0.45*
Age<13 years at anal sex debut<br>N=433[c], Obs=716[d] ; N=429[e], Obs=710[f]	No<br>Yes	1<br>0.71 [0.26, 1.94]	0.51	1<br>0.72 [0.27, 1.97]	*0.526*
Any IPV victimization<br>N=436[c], Obs=743[d] ; N=432[e], Obs=737[f]	No/missing<br>Yes	1<br>2.45 [1.63, 3.67]	*<0.001*	1<br>2.56 [1.70, 3.85]	*<0.001*
Any IPV victimization in the past year<br>N=436[c], Obs=743[d] ; N=432[e], Obs=737[f]	No/missing<br>Yes	1<br>2.82 [1.88, 4.22]	*<0.001*	1<br>2.96 [1.97, 4.46]	*<0.001*
Any IPV perpetration<br>N=436[c], Obs=743[d] ; N=432[e], Obs=737[f]	No/missing<br>Yes	1<br>2.83 [1.89, 4.22]	*<0.001*	1<br>2.86 [1.90, 4.30]	*<0.001*
Any IPV perpetration in the past year<br>N=436[c], Obs=743[d] ; N=432[e], Obs=737[f]	No/missing<br>Yes	1<br>3.40 [2.13, 5.41]	*<0.001*	1<br>3.36 [2.06, 5.47]	*<0.001*
Internalised homophobia<br>N=436[c], Obs=743[d] ; N=432[e], Obs=737[f]	No/missing<br>Yes	1<br>1.92 [1.30, 2.83]	*0.001*	1<br>1.91 [1.28, 2.83]	*0.001*
Concealment of sexual identity<br>N=434[c], Obs=727[d] ; N=431[e], Obs=722[f]	No<br>Yes	1<br>1.75 [1.17, 2.62]	*0.007*	1<br>1.71 [1.13, 2.59]	*0.012*

[a] p-value by Wald test using GEE models.
[b] Age (included as four categories: <25, 25-29, 30-39, 40+), born in the UK, sexual identity (gay or bisexual/straight), university education, London study clinic site, calendar time (year & calendar quarter), and follow-up time-point (month-12, month-24).
[c] Number of men contributing observations to the unadjusted model.
[d] Number of observations examined in the unadjusted model.
[e] Number of men contributing observations to the adjusted model.
[f] Number of observations examined in the adjusted model.

For peer review only - http://bmjopen.bmj.com/site/about/guidelines.xhtml

Textbox 1: Prevalence of depression on symptom questionnaires in studies of UK GBMSM

Study	Recruitment dates	Sampling strategy	Size of GBMSM sample	Measure of depression	Depression prevalence
Cross-sectional survey of GBMSM in London[16]	1999	Consecutive sampling of GBMSM attending a GUM clinic	122	HADS score of ≥8	18.8%
Cross-sectional survey of GBMSM in London[18]	2010-2011	Consecutive sampling of GBMSM attending GUM clinics	519	HADS score of ≥8	12%
Natsal-3[13]	2010-2012	Multistage probability sampling of the general British population	190	PHQ-2 score of ≥3	8.9%
Cross-sectional survey of GBMSM in London[15]	2016	Volunteer sampling of users of a geosocial-networking app for GBMSM	179	PHQ-2 score of ≥3	22.3%
Gay & Bisexual Men's Health Survey[14]	2011	Snowball sampling of GBMSM	5416	PHQ-9 score of ≥10	21.3%
AURAH[17]	2013-2014	Consecutive sampling of HIV-negative GBMSM attending GUM clinics in England	1340 sexually active GBMSM	PHQ-9 score of ≥10	12.4%
PROUD	2012-2014 baseline<br>2013-2015 month-12<br>2014-2016 month-24	Volunteer sampling of HIV-negative GBMSM attending GUM clinics and community organizations, and GBMSM users of social media	540<br>410<br>333	PHQ-9 score of ≥10	9.1%<br>14.4%<br>14.4%

For peer review only - http://bmjopen.bmj.com/site/about/guidelines.xhtml

References:

1. King M, Semlyen J, Tai SS, et al. A systematic review of mental disorder, suicide, and deliberate self harm in lesbian, gay and bisexual people. *BMC Psychiatry* 2008;8(70) doi: 10.1186/1471-244X-8-70
2. Meyer IH. Prejudice, social stress, and mental health in lesbian, gay, and bisexual populations: conceptual issues and research evidence. *Psychol Bull* 2003;129(5):674-97. doi: 10.1037/0033-2909.129.5.674 [published Online First: 2003/09/06]
3. Klein H. Depression and HIV Risk Taking among Men Who Have Sex with Other Men (MSM) and Who Use the Internet to Find Partners for Unprotected Sex. *J Gay Lesbian Ment Health* 2014;18(2):164-89. doi: 10.1080/19359705.2013.834858 [published Online First: 2014/01/01]
4. Perdue TE, Hagan H, Thiede H, et al. Depression and HIV risk behaviour among Seattle-area injection drug users and young men who have sex with men. *AIDS Educ Prev* 2003;15(1):81-92.
5. Mimiaga MJ, Biello KB, Robertson AM, et al. High prevalence of multiple syndemic conditions associated with sexual risk behavior and HIV infection among a large sample of Spanish- and Portuguese-speaking men who have sex with men in Latin America. *Arch Sex Behav* 2015;44(7):1869-78. doi: 10.1007/s10508-015-0488-2 [published Online First: 2015/07/15]
6. Cochran SD, Mays VM. Burden of psychiatric morbidity among lesbian, gay, and bisexual individuals in the California Quality of Life Survey. *J Abnorm Psychol* 2009;118(3):647-58. doi: 10.1037/a0016501 [published Online First: 2009/08/19]
7. Stall R, Mills TC, Williamson J, et al. Association of co-occurring psychosocial health problems and increased vulnerability to HIV/AIDS among urban men who have sex with men. *Am J Public Health* 2003;93(6):939-42. [published Online First: 2003/05/30]
8. Buller AM, Devries KM, Howard LM, et al. Associations between intimate partner violence and health among men who have sex with men: a systematic review and meta-analysis. *PLoS Med* 2014;11(3):e1001609. doi: 10.1371/journal.pmed.1001609 [published Online First: 2014/03/07]
9. Bourne A, Reid D, Hickson F, et al. "Chemsex" and harm reduction need among gay men in South London. *Int J Drug Policy* 2015;26(12):1171-6. doi: 10.1016/j.drugpo.2015.07.013 [published Online First: 2015/08/25]
10. Sewell J, Miltz A, Lampe FC, et al. Poly drug use, chemsex drug use, and associations with sexual risk behaviour in HIV-negative men who have sex with men attending sexual health clinics. *Int J Drug Policy* 2017;43:33-43. doi: 10.1016/j.drugpo.2017.01.001 [published Online First: 2017/02/13]
11. Pufall EL, Kall M, Shahmanesh M, et al. Sexualized drug use ('chemsex') and high-risk sexual behaviours in HIV-positive men who have sex with men. *HIV Med* 2018;19(4):261-70. doi: 10.1111/hiv.12574 [published Online First: 2018/01/26]
12. Bourne A, Reid D, Hickson F, et al. The Chemsex Study: drug use in sexual settings among gay and bisexual men in Lambeth, Southwark & Lewisham: Sigma Research, 2014.
13. Mercer CH, Prah P, Field N, et al. The health and well-being of men who have sex with men (MSM) in Britain: Evidence from the third National Survey of Sexual Attitudes and Lifestyles (Natsal-3). *BMC Public Health* 2016;16(525) doi: 10.1186/s12889-016-3149-z
14. Hickson F, Davey C, Reid D, et al. Mental health inequalities among gay and bisexual men in England, Scotland and Wales: a large community-based cross-sectional survey. *J Public Health (Oxf)* 2017;39(2):266-73. doi: doi:10.1093/pubmed/fdw021
15. Goedel WC, Hagen D, Halkitis PN, et al. Post-exposure prophylaxis awareness and use among men who have sex with men in London who use geosocial-networking smartphone applications. *AIDS Care* 2017;29(5):579-86. doi: 10.1080/09540121.2016.1259455 [published Online First: 2016/12/03]
16. Beck A, McNally I, Petrak J. Psychosocial predictors of HIV/STI risk behaviours in a sample of homosexual men. *Sex Transm Infect* 2003;79(2):142-6. [published Online First: 2003/04/12]
17. Miltz AR, Rodger AJ, Sewell J, et al. Clinically significant depressive symptoms and sexual behaviour among men who have sex with men. *The British Journal of Psychiatry Open* 2017;3(3):127-37. doi: 10.1192/bjpo.bp.116.003574 [published Online First: 2017/05/17]
18. Bacchus LJ, Buller AM, Ferrari G, et al. Occurrence and impact of domestic violence and abuse in gay and bisexual men: A cross sectional survey. *Int J STD AIDS* 2017;28(1):16-27. doi: 10.1177/0956462415622886 [published Online First: 2016/01/09]
19. McCormack S, Dunn DT, Desai M, et al. Pre-exposure prophylaxis to prevent the acquisition of HIV-1 infection (PROUD): effectiveness results from the pilot phase of a pragmatic open-label randomised

For peer review only - http://bmjopen.bmj.com/site/about/guidelines.xhtml

trial. *Lancet* 2016;387(10013):53-60. doi: 10.1016/s0140-6736(15)00056-2 [published Online First: 2015/09/14]
20. Kroenke K, Spitzer RL, Williams JB. The PHQ-9: validity of a brief depression severity measure. *J Gen Intern Med* 2001;16(9):606-13. [published Online First: 2001/09/15]
21. Babor TF, Higgins-Biddle JC, Saunders JB, et al. The Alcohol Use Disorders Identification Test. Guidelines for Use in Primary Care: Department of Mental Health and Substance Dependence, World Health Organization, 2001.
22. Ross MW, Rosser BR. Measurement and correlates of internalized homophobia: a factor analytic study. *J Clin Psychol* 1996;52(1):15-21. doi: 10.1002/(sici)1097-4679(199601)52:1<15::aid-jclp2>3.0.co;2-v [published Online First: 1996/01/01]
23. Zou G. A modified poisson regression approach to prospective studies with binary data. *Am J Epidemiol* 2004;159(7):702-6. [published Online First: 2004/03/23]
24. STATA statistical software. 13 ed [program]. Texas, 2009.
25. Defechereux PA, Mehrotra M, Liu AY, et al. Depression and Oral FTC/TDF Pre-exposure Prophylaxis (PrEP) Among Men and Transgender Women Who Have Sex With Men (MSM/TGW). *AIDS Behav* 2016;20(7):1478-88. doi: 10.1007/s10461-015-1082-2 [published Online First: 2015/06/17]
26. Miltz AR, Lampe FC, Bacchus LJ, et al. Intimate partner violence, depression, and sexual behaviour among gay, bisexual and other men who have sex with men in the PROUD trial. *BMC Public Health* 2019;19(1):431. doi: 10.1186/s12889-019-6757-6 [published Online First: 2019/04/27]
27. Paranjothy S, Gallacher J, Amlot R, et al. Psychosocial impact of the summer 2007 floods in England. *BMC Public Health* 2011;11:145. doi: 10.1186/1471-2458-11-145 [published Online First: 2011/03/05]
28. Vanden Berghe W, Nostlinger C, Laga M. Syndemic and other risk factors for unprotected anal intercourse among an online sample of Belgian HIV negative men who have sex with men. *AIDS Behav* 2014;18(1):50-8. doi: 10.1007/s10461-013-0516-y [published Online First: 2013/05/18]
29. Weatherburn P, Hickson F, Reid D, et al. Motivations and values associated with combining sex and illicit drugs ('chemsex') among gay men in South London: findings from a qualitative study. *Sex Transm Infect* 2017;93:153-54.
30. Mimiaga MJ, Noonan E, Donnell D, et al. Childhood sexual abuse is highly associated with HIV risk-taking behavior and infection among MSM in the EXPLORE Study. *J Acquir Immune Defic Syndr* 2009;51(3):340-8. doi: 10.1097/QAI.0b013e3181a24b38 [published Online First: 2009/04/16]
31. Brown MJ, Serovich JM, Kimberly JA. Depressive Symptoms, Substance Use and Partner Violence Victimization Associated with HIV Disclosure Among Men Who have Sex with Men. *AIDS Behav* 2016;20(1):184-92. doi: 10.1007/s10461-015-1122-y [published Online First: 2015/07/01]
32. Wang HY, Wang N, Chu ZX, et al. Intimate Partner Violence Correlates With A Higher HIV Incidence Among MSM: A 12-Month Prospective Cohort Study in Shenyang, China. *Sci Rep* 2018;8(1):2879. doi: 10.1038/s41598-018-21149-8 [published Online First: 2018/02/15]
33. De Boni RB, Machado IK, De Vasconcellos MTL, et al. Syndemics among individuals enrolled in the PrEP Brasil Study. *Drug and alcohol dependence* 2018;185:168-72. doi: 10.1016/j.drugalcdep.2017.12.016 [published Online First: 2018/02/20]
34. Jiang H, Chen X, Li J, et al. Predictors of condom use behavior among men who have sex with men in China using a modified information-motivation-behavioral skills (IMB) model. *BMC Public Health* 2019;19(1):261. doi: 10.1186/s12889-019-6593-8 [published Online First: 2019/03/06]
35. Fife RS, Schrager SS. Family violence. What health care providers need to know. Sudbury, MA: Jones & Bartlett Learning, LLC 2012.
36. Beck AT. The evolution of the cognitive model of depression and its neurobiological correlates. *Am J Psychiatry* 2008;165(8):969-77. doi: 10.1176/appi.ajp.2008.08050721 [published Online First: 2008/07/17]
37. Herek GM, Sims C, editors. *Sexual orientation and violent victimization: hate crimes and intimate partner violence among gay and bisexual males in the United States*. New York: Oxford University Press, 2008.
38. Goldenberg T, Stephenson R, Freeland R, et al. “Struggling to be the alpha”: Sources of tension and intimate partner violence in same-sex relationships between men. *Culture, Health & Sexuality* 2016;18:875-89.
39. Operario D, Fiske ST. Ethnic identity moderates perceptions of prejudice: judgments of personal versus group discrimination and subtle versus blatant bias. *Personality and Social Psychology Bulleting* 2001;27:550-61.
40. Equality Act 2010. 2010 CHAPTER 15 In: Parliament U, ed.
41. Marriage (Same Sex Couples) Act 2013. 2013 CHAPTER 30. In: Parliament U, ed.

For peer review only - http://bmjopen.bmj.com/site/about/guidelines.xhtml

42. Gyimah S. Thousands officially pardoned under 'Turing's Law': Ministry of Justice, 2017.
43. BBC. Same-sex marriage now legal in Northern Ireland: BBC newsround; 2019 [Available from: https://www.bbc.co.uk/newsround/50135994 accessed October 22 2019.

For peer review only

For peer review only - http://bmjopen.bmj.com/site/about/guidelines.xhtml

STROBE Statement—Checklist of items that should be included in reports of ***cross-sectional studies***

	Item No	Recommendation	Page No
**Title and abstract**	1	(*a*) Indicate the study's design with a commonly used term in the title or the abstract	1-2
		(*b*) Provide in the abstract an informative and balanced summary of what was done and what was found	2
**Introduction**			
Background/rationale	2	Explain the scientific background and rationale for the investigation being reported	3-4
Objectives	3	State specific objectives, including any prespecified hypotheses	4
**Methods**			
Study design	4	Present key elements of study design early in the paper	5-8
Setting	5	Describe the setting, locations, and relevant dates, including periods of recruitment, exposure, follow-up, and data collection	5
Participants	6	(*a*) Give the eligibility criteria, and the sources and methods of selection of participants	5
Variables	7	Clearly define all outcomes, exposures, predictors, potential confounders, and effect modifiers. Give diagnostic criteria, if applicable	5-7
Data sources/ measurement	8*	For each variable of interest, give sources of data and details of methods of assessment (measurement). Describe comparability of assessment methods if there is more than one group	5-7
Bias	9	Describe any efforts to address potential sources of bias	7-8
Study size	10	Explain how the study size was arrived at	5
Quantitative variables	11	Explain how quantitative variables were handled in the analyses. If applicable, describe which groupings were chosen and why	7-8
Statistical methods	12	(*a*) Describe all statistical methods, including those used to control for confounding	7-8
		(*b*) Describe any methods used to examine subgroups and interactions	/
		(*c*) Explain how missing data were addressed	5-8
		(*d*) If applicable, describe analytical methods taking account of sampling strategy	7-8
		(*e*) Describe any sensitivity analyses	8
**Results**			
Participants	13*	(a) Report numbers of individuals at each stage of study—eg numbers potentially eligible, examined for eligibility, confirmed eligible, included in the study, completing follow-up, and analysed	8-9
		(b) Give reasons for non-participation at each stage	8-9
		(c) Consider use of a flow diagram	/
Descriptive data	14*	(a) Give characteristics of study participants (eg demographic, clinical, social) and information on exposures and potential confounders	8-9
		(b) Indicate number of participants with missing data for each variable of interest	8
Outcome data	15*	Report numbers of outcome events or summary measures	9
Main results	16	(*a*) Give unadjusted estimates and, if applicable, confounder-adjusted estimates and their precision (eg, 95% confidence interval). Make clear which confounders were adjusted for and why they were included	9-10

For peer review only

		(*b*) Report category boundaries when continuous variables were categorized	/
		(*c*) If relevant, consider translating estimates of relative risk into absolute risk for a meaningful time period	/
Other analyses	17	Report other analyses done—eg analyses of subgroups and interactions, and sensitivity analyses	8
**Discussion**			
Key results	18	Summarise key results with reference to study objectives	11
Limitations	19	Discuss limitations of the study, taking into account sources of potential bias or imprecision. Discuss both direction and magnitude of any potential bias	13
Interpretation	20	Give a cautious overall interpretation of results considering objectives, limitations, multiplicity of analyses, results from similar studies, and other relevant evidence	13
Generalisability	21	Discuss the generalisability (external validity) of the study results	12
**Other information**			
Funding	22	Give the source of funding and the role of the funders for the present study and, if applicable, for the original study on which the present article is based	14

*Give information separately for exposed and unexposed groups.

Note: An Explanation and Elaboration article discusses each checklist item and gives methodological background and published examples of transparent reporting. The STROBE checklist is best used in conjunction with this article (freely available on the Web sites of PLoS Medicine at http://www.plosmedicine.org/, Annals of Internal Medicine at http://www.annals.org/, and Epidemiology at http://www.epidem.com/). Information on the STROBE Initiative is available at www.strobe-statement.org.

For peer review only

BMJ Open

Prevalence and correlates of depressive symptoms among gay, bisexual and other men who have sex with men in the PROUD randomized clinical trial of HIV pre-exposure prophylaxis

Journal:	*BMJ Open*
Manuscript ID	bmjopen-2019-031085.R2
Article Type:	Original research
Date Submitted by the Author:	04-Nov-2019
Complete List of Authors:	Miltz, Ada; University College London<br>Lampe, F; University College London<br>McCormack, Sheena; University College London, MRC CTU<br>Dunn, David; University College London, MRC CTU<br>White, Ellen; University College London, MRC CTU<br>Rodger, Alison; University College London<br>Phillips, Andrew; University College London<br>Sherr, Lorraine; University College London<br>Sullivan, Ann ; Chelsea and Westminster Healthcare NHS Trust<br>Reeves, Iain; Homerton University Hospital NHS Trust, Sexual Health<br>Clarke, Amanda; Elton John Centre, Sussex House<br>Gafos, Mitzy; London School of Hygiene and Tropical Medicine
**Primary Subject Heading**:	Epidemiology
Secondary Subject Heading:	Mental health, Public health
Keywords:	MENTAL HEALTH, EPIDEMIOLOGY, GENITOURINARY MEDICINE, PUBLIC HEALTH

SCHOLARONE™
Manuscripts

Prevalence and correlates of depressive symptoms among gay, bisexual and other men who have sex with men in the PROUD randomized clinical trial of HIV pre-exposure prophylaxis

Ada Miltz[1*], Fiona C Lampe[1], Sheena McCormack[2], David Dunn[2], Ellen White[2], Alison Rodger[1], Andrew Phillips[1], Lorraine Sherr[1], Ann K Sullivan[3], Iain Reeves[4], Amanda Clarke[5], Mitzy Gafos[6]

*Corresponding author, email: Ada.Miltz.11@ucl.ac.uk, telephone number: +44 20801 68058

Author affiliations

[1]Institute for Global Health, University College London, London, UK

[2] MRC Clinical Trials Unit, University College London, London, UK

[3] Chelsea and Westminster Hospital NHS Trust, London, UK

[4] Homerton Hospital NHS Trust, London, UK

[5] Brighton & Sussex University Hospital NHS Trust, Brighton & Sussex Medical School, Brighton, UK

[6] Department of Global Health and Development, London School of Hygiene and Tropical Medicine, London, UK

For peer review only

For peer review only - http://bmjopen.bmj.com/site/about/guidelines.xhtml

Abstract

Objectives: The aim of this analysis is to: (i) assess the prevalence of clinically significant depressive symptoms at baseline and follow-up for participants in the PROUD trial of HIV pre-exposure prophylaxis (PrEP), examining changes in prevalence over time and (ii) investigate the association of socio-economic and psychosocial factors with depression.

Methods: PROUD was an open label randomised trial evaluating the benefit of PrEP for 544 HIV-negative gay, bisexual, and other men who have sex with men (GBMSM) in England. Enrolment was between 2012 and 2014, with at least two years follow-up. Prevalence of depression (score≥10 on PHQ-9, Patient Health Questionnaire) was assessed and compared across time-points (using McNemar's χ^2 tests) and between trial arms (using χ^2 tests). Cross-sectional associations with socio-economic and psychosocial factors were examined using baseline data in modified Poisson regression models and combined 12- and 24-month follow-up data in generalized estimating equations (GEEs). Prevalence ratios were presented unadjusted and adjusted (aPR) for age, UK birth, sexual identity, university education, London study clinic site, and calendar time (and follow-up time-point in GEEs).

Results: Depression increased significantly from baseline (9.1%; 49/540) to the 12-month (14.4%; 59/410) and 24-month (14.4%; 48/333) follow-ups, possibly explained by underreporting at baseline. The prevalence of depression did not differ by study trial arm, at any time-point. In the baseline analysis, younger age, unemployment, and crystal methamphetamine use, was associated with depression. In combined analysis of 12- and 24-month data, measures of intimate partner violence (IPV) (lifetime IPV victimization aPR 2.57 [95% CI: 1.71, 3.86]), internalized homophobia (aPR 1.91 [95% CI: 1.29, 2.83]), and concealment of sexual identity (aPR 1.75 [95% CI: 1.16, 2.65]), were strongly associated with depression.

Conclusions: There is a high concomitant burden of psychosocial factors with depression among GBMSM.

Trial registration: ISRCTN (ISRCTN94465371) and ClinicalTrials.gov (NCT02065986).

For peer review only - http://bmjopen.bmj.com/site/about/guidelines.xhtml

Strengths and limitations of this study

- This is the first UK study to collect data on depression prevalence over time among gay, bisexual and other men who have sex with men (GBMSM).
- This is the first UK study to investigate associations between a range of psychosocial factors and depression among GBMSM.
- Since the data were analysed cross-sectionally, the direction of associations may be unclear for some factors.
- This study may have lacked power to detect the presence of some associations.
- There is a possibility of unmeasured confounding in this study, as information on childhood sexual abuse, income, and levels of social support was not collected.

Introduction

There is consistent evidence that depression prevalence is elevated among gay and bisexual men compared to their heterosexual counterparts in high-income countries [1]. Two theoretical models have put forward explanations for the elevated prevalence observed among sexual minorities: minority stress theory and syndemic theory. Minority stress theory describes the psychosocial consequences of being in continual conflict with a discriminatory and heteronormative social environment. Perpetual negative feedback from others is thought to lead to a process of self-stigmatization termed internalized homophobia, whereby antigay social values/attitudes are directed towards the self. Internalized homophobia often results in deep conflict and poor self-regard with negative consequences such as pervasive expectations of rejection in one's life and concealment of one's sexual identity. Concealment adds to mental distress by disallowing individuals to affiliate with people of the same sexual identity [2]. Two U.S. studies of gay, bisexual and other men who have sex with men (GBMSM) found strong associations between perceived discrimination on the basis of sexual orientation and depressive symptoms [3 4]. Internalized homophobia may explain this association.

Syndemic theory proposes that concomitant widespread use of recreational drugs and experiences of intimate partner violence (IPV) among sexual minority men may be to blame for elevated levels of depression, as causal, and bidirectional, relationships are postulated between drug use, IPV, and depression. The synergistic interaction of these co-occurring factors is thought to result in an exaggerated risk of poor health outcomes [5]. A number of U.S. studies have found strong associations of recreational drug use [3 6 7] and IPV [8] with depressive symptoms. Recently, studies have also found a

For peer review only - http://bmjopen.bmj.com/site/about/guidelines.xhtml

link between chemsex and depression [9-11]. Chemsex is a cultural phenomenon among a sub-group of gay identified men, which was first described in the UK [12]. It is the intentional use of psychoactive substances (usually one or more of mephedrone, gamma-hydroxybutrate/gamma-butyrolactone [GHB/GBL] and methamphetamine) to stimulate sexual arousal, facilitate different sexual practices, and prolong sexual episodes.

A number of UK studies have examined the prevalence of, and factors associated with, depressive symptoms among GBMSM [13-18]. In several of these studies however, power to assess associations was limited due to small sample size (<200 GBMSM) [13 15 16], and one limitation of the larger studies is that data were not collected on possible psychosocial risk factors (internalized homophobia and IPV) [14 17 18]. There is a need to better understand correlates of depression among GBMSM in the UK.

This analysis uses data from the PROUD randomised clinical trial, which evaluated the impact of pre-exposure prophylaxis (PrEP) on HIV incidence, and collected data on a range of psychosocial factors. The aim is to: (i) present the prevalence of depressive symptoms at baseline and follow-up, examining changes in prevalence over time and (ii) drawing on minority stress theory and syndemic theory, investigate the association of demographic, socio-economic and psychosocial factors with depressive symptoms.

Methods

PROUD trial

The PROUD trial was a multi-centre, pragmatic open label randomised trial evaluating the benefit of PrEP as part of a package of HIV risk reduction interventions for HIV-negative GBMSM and trans women [19]. Only three trans women enrolled in PROUD, and therefore data cannot be presented separately for trans women. The study was reviewed and approved by London Bridge Research Ethics Committee (12/LO/1289).

Routine attenders at 13 genitourinary medicine (GUM) clinics in England were enrolled between November 2012 and April 2014. Volunteers were eligible if they met the following criteria: male sex at birth; aged 18 years or over; HIV-negative on the day of enrolment or in the past four weeks; reported condomless anal sex (CAS) in the past three months; and expected to have CAS again in the next three months [19]. Volunteers were randomised 1:1 to an immediate start of daily oral PrEP or a deferred start after one year. However, during follow-up, an unexpectedly high incidence of HIV was

For peer review only - http://bmjopen.bmj.com/site/about/guidelines.xhtml

observed in the deferral arm (9.0 per 100 person-years, 90% CI: 6.1, 12.8), which led to the decision by the trial steering committee in October 2014 to offer all participants PrEP. Participants had the opportunity to remain in follow-up for at least two years.

Patient and Public Involvement

The PROUD pilot study team used a number of mechanisms to promote effective and appropriate participant engagement in the research project (http://www.proud.mrc.ac.uk/about/patient-and-public-involvement-ppi/). A number of meetings or discussion forums were hosted throughout the course of the study, in which participants were actively involved in the research process. Participants were informed of these forums via the study team, advertisements in the clinics, or the study website. The types of topics participants were involved in included: (i) development of the trial protocol, including questionnaires, participant information sheets and consent forms, (ii) assessing and improving study procedures, recruitment and retention strategies, (iii) assessing and improving public involvement and communication plans, and (iv) development of plans for result dissemination and post-study access to study product. Patient and public involvement activities with a small group of PROUD participants (N=13), in the form of a short qualitative survey, was also undertaken as part of this depression project; responses are considered in the discussion.

Hypothesized relationships and selection of variables

Causal relationships with depressive symptoms were hypothesized for socio-economic factors (younger age, lower levels of educational attainment, unemployment, and having no ongoing relationship) and psychosocial measures (IPV, early age at anal sex debut [a possible marker of childhood sexual abuse], recreational drug use, alcohol use, internalized homophobia, and concealment of sexual identity) collected in PROUD. Hypotheses were based on minority stress theory and syndemic theory as well as observed relationships in previous studies, although such relationships have not been proved to be causal.

Measures

Depressive symptoms

The Patient Health Questionnaire (PHQ-9) was used to measure the prevalence of depressive symptoms at baseline and on an approximately annual basis (month-12 and 24). In the PHQ-9, participants are asked to rate how often over the past two weeks they have experienced nine specific problems (anhedonia, feeling hopeless, trouble sleeping, feeling tired, appetite problems,

For peer review only - http://bmjopen.bmj.com/site/about/guidelines.xhtml

feeling like a failure, trouble concentrating, being slow/restless, and suicidal ideation/self-harm) [20]. Response options range from 'not at all' (coded as 0) to 'nearly every day' (coded as 3). In this paper, a missing response was coded as 0. Summing responses from each question generates a depressive symptom score. A cut-off point of 10 or greater for the total score indicates a possible clinically significant depressive condition [20]. In a U.S. validation study of primary care and obstetrics/ gynaecology patients, the vast majority of patients (93%) with no depressive disorder diagnosed by a (blinded) mental health professional had a PHQ-9 score less than 10, while 88% with a major depression diagnosis had scores of 10 or greater [20].

The PROUD study team reported that part way through the trial enrolment process a change in PHQ-9 reporting policy was instated, which continued to be utilized throughout follow-up. From April 2013, participants were informed that after self-completing the questionnaire they would be asked to disclose their response to the suicidal/self-harm ideation question on the PHQ-9 ('Thoughts that you would be better off dead, or of hurting yourself in some way') to trial clinical staff. The Trial Steering Committee recommended this change in reporting policy as a result of one man attempting to take his life during the course of the study.

At the baseline visit men were asked by study staff to note down any medication they were taking. All men who specified that they were taking a medication that has been licensed for treatment of depression were identified.

Intimate partner violence

At month-12 and 24, participants were asked five questions about IPV victimization and five questions about IPV perpetration, based on the 'Health and Relationships survey' [18]. Each question had the following four response options: never, more than 1 year ago, within the last year with former partner, and within the last year with current partner. The five IPV victimization questions included the following: 'Have you ever felt frightened of the behaviour of a partner', 'Have you ever needed to ask a partner's permission to work, go shopping, visit relatives, or visit friends (i.e. beyond the usual of being considerate to and checking with a partner)', 'Have you ever been hit, slapped, kicked or otherwise physically hurt by a partner', 'Have you ever been forced to have sex or made to engage in some sexual activity when you did not want to', and 'Have you ever been forced to have sex without a condom when you did not want to'. The five IPV perpetration questions were identical but asked whether the participant had perpetrated these behaviours. A positive response to any one

For peer review only

of the five respective questions was considered to indicate lifetime IPV victimization or lifetime IPV perpetration. A missing response was included in the 'no IPV' category.

Age at first anal sex

Data on age at anal sex debut with a male were collected at month-12 and 24. Reporting anal intercourse at: (i) 12 years or younger and (ii) 15 years or younger, was investigated. Measure (i) may include experiences of childhood sexual abuse (CSA), however, since the age of the partner and the nature of the encounter was not known, this measure may not reflect or identify all sexual abuse experienced.

Recreational drug use

At baseline, participants were asked to report whether they had used recreational drugs in the past three months. Chemsex drug use was defined as use of mephedrone, GHB/GBL, and/or methamphetamine in the past three months. It is of note that men were not asked in PROUD whether these drugs had been taken before or during sex. At the 12-month and 24-month questionnaires, participants were asked whether, in the past three months, they had engaged in sex after using recreational drugs, which may also be a proxy measure for chemsex.

Alcohol use

At baseline, a measure of higher risk alcohol consumption was investigated based on the first two questions of the WHO AUDIT-C questionnaire [21]. Participants were asked about the frequency of their alcohol consumption in the past three months, with response options: never (coded as 0), monthly or less (codes as 1), 2 or 3 times a month (coded as 2), once or twice a week (coded as 3), 3 or more times a week (coded as 4). Participants were also asked about their usual alcohol intake in units on a typical day when they are drinking, with responses 1-2 units (coded as 0), 3-4 (coded as 1), 5-6 (coded as 2), 7-9 (coded as 3), and 10+ (coded as 4). Summing responses from both questions resulted in an alcohol use score ranging from 0 to 8. A score of ≥6 was considered to indicate higher risk alcohol consumption. A missing response was included in the lower risk/no alcohol consumption category (score of <6).

Internalized homophobia

At month-12 and 24, participants were asked eight questions surrounding attitudes towards gay sexuality, based on the 26-item Internalized Homophobia Scale (four scales with internal reliabilities of 0.85, 0.69, 0.64, and 0.62) [22]. Each question had the following response options: strongly agree,

For peer review only - http://bmjopen.bmj.com/site/about/guidelines.xhtml
For peer review only

agree, neutral or uncertain, disagree, and strongly disagree. The eight questions included the following: (i) 'I feel comfortable in gay bars', (ii) 'I feel comfortable being seen in public with an obviously transgender/gay person', (iii) 'I feel comfortable discussing homosexuality in a public situation', (iv)'I feel comfortable being a transgender/gay man', (v) 'Homosexuality is morally acceptable to me', (vi) 'Even if I could change my sexual orientation I wouldn't', (vii) 'Obviously effeminate homosexual men make me feel uncomfortable' and (viii) 'Social situations with transgender/gay men make me feel uncomfortable'. Responding with 'strongly agree' or 'agree' to questions (vii) or (viii), or with 'strongly disagree' or 'disagree' to questions (i), (ii), (iii), (iv), (v), or (vi) was considered to indicate internalized homophobia.

Concealment of sexual identity

At month-12 and 24, participants were also asked how many of their (i) work colleagues, (ii) friends, and (iii) close family, know that they are gay/transgender/have sex with men. Response options for groups (i)-(iii) included: all/almost all, >half, <half, few, and none. Participants who did not report being 'out' to all/almost all friends, work colleagues and close family were considered to indicate concealment of their sexual identity.

Statistical analysis

Prevalence of depressive symptoms (PHQ-9≥10) was compared between trial arms at each time-point using χ^2 tests and across time-points (trial arms combined) using McNemar's χ^2 test to account for paired responses.

Unadjusted and adjusted associations of demographic, socio-economic, and psychosocial (drinking and drug use) factors with depressive symptoms (PHQ-9≥10) at baseline were assessed using modified Poisson regression with a robust variance estimator, in order to produce prevalence ratios (PRs) [23]. Seven socio-demographic factors assumed not to be on hypothesized causal pathways were adjusted for: Age group (<25, 25-29, 30-39, or 40+ years), born in the UK (yes or no/missing), sexual identity (gay or bisexual/straight), university education (yes or no/missing), ongoing relationship (yes or no/missing), London study clinic site (yes or no), and calendar time (year and calendar quarter). For each factor investigated, separate socio-demographic adjusted models were constructed.

Generalized estimating equations (GEEs) were used to assess the cross-sectional association of psychosocial factors (sexualized drug use, age at anal sex debut, IPV, internalized homophobia, 'outness') with depressive symptoms, including data from the 12 and 24-month questionnaires.

For peer review only - http://bmjopen.bmj.com/site/about/guidelines.xhtml

GEEs were used to account for multiple responses from individual participants, and were fitted using Poisson models with a log link and compound symmetry for the correlational structure. In the GEEs, adjustment was made for the same set of baseline socio-demographic factors described above; with the exceptions that age and calendar time were included as time-updated variables, baseline 'ongoing relationship' was not adjusted for as this status may change over time, and additional adjustment was made for follow-up time-point (month-12, month-24). Use of GEE models in this way represents a pooled cross-sectional analysis. A longitudinal analysis was not feasible as the number of 'incident' cases of PHQ-9 depression was low; of the 233 men who appeared to have no history of depression based on PROUD data (PHQ-9<10 at baseline and month-12 and no antidepressant use, as captured at baseline only), 22 reported clinically significant symptoms (PHQ-9≥10) at month-24.

For each measure and at each time-point, the proportion of missing responses was <5%. For psychosocial measures (depression, drinking, drug use, sexualized drug use, internalized homophobia, and IPV), missing responses were considered to indicate that the event did not occur. There appeared to be a common response pattern in which only those experiences that had occurred were ticked on the questionnaire. Given the very low proportion of missing responses, missing values were considered to indicate a lower level of educational attainment (i.e. no university degree), no ongoing relationship, and no current employment. A sensitivity analysis was undertaken excluding missing values when defining each variable. The findings were very similar to the main analysis. All analyses were performed in STATA statistical software (version 13) [24] and reported according to the STROBE guidelines.

Results

There were 544 participants enrolled in PROUD. In total, 540 completed a baseline questionnaire, of whom 410 (76%) completed a 12-month questionnaire, and 333 (62%) completed a 24-month questionnaire. Overall, 96% of men identified as gay and 3% as bisexual (includes three trans women). Eighty-two percent of men were of white ethnicity; median age was 35 years (Interquartile range [IQR]: 29-42 years) at baseline (Table 1). The prevalence of psychosocial measures collected at baseline and follow-up time-points are presented in Tables 1 and 2. Briefly, lifetime IPV was reported by 45% of participants for victimization and 20% for perpetration at month-12, and 42% of participants indicated internalized homophobia at month-12. The prevalence of CAS with ≥2 partners in the past three months was: 76% at baseline, 74% at month-12, and 75% at month-24.

For peer review only - http://bmjopen.bmj.com/site/about/guidelines.xhtml

Prevalence of depressive symptoms across trial arms and time-points

The prevalence of depressive symptoms was 9.1% (49/540) at baseline, 14.4% (59/410) at month-12, and 14.4% (48/333) at month-24. In terms of changes in individual depression status between time-points, among the 410 men who completed the baseline and 12-month questionnaire, 38 men reported depressive symptoms (PHQ-9≥10) at baseline, 22 (57.9%) of whom continued to report this measure of depression at month-12. Of the 372 men who did not report depressive symptoms at baseline, 37 (10.0%) reported it at month-12. Among the 333 men who completed the baseline and 24-month questionnaire, 32 men who reported depressive symptoms (PHQ-9≥10) at baseline, 11 (34.4%) of whom continued to report this measure of depression at month-24. Of the 301 men who did not report depressive symptoms at baseline, 37 (12.3%) reported it at month-24. Finally, among the 307 men who completed both the 12- and 24-month questionnaire, of 43 men who reported depressive symptoms (PHQ-9≥10) at month-12, 16 (37.2%) continued to report this measure of depression at month-24. Of the 264 men who did not report depression at month-12, 29 (11.0%) reported it at month-24.

The prevalence of depressive symptoms was slightly higher in the immediate than the deferred PrEP group at baseline (10.6% versus 7.5%) and at subsequent time-points (16.7% versus 11.9% at month-12; 16.5% versus 11.9% at month-24). Differences between randomised groups were not statistically significant at any time-point (p≥0.2). The within-person increase in prevalence from 9.3% at baseline to 14.4% at month-12 (among 410 men) and from 9.6% at baseline to 14.4% at month-24 (among 333 men) was significant (p≤0.04), but there was no difference from 14.0% at month-12 to 14.7% at month-24 (among 307 men).

Overall, 8.5% (46/540) of men reported antidepressant use at baseline; 20.4% (n=10) of men with depressive symptoms (PHQ-9≥10) and 7.3% (n=36) of men without. There were 85 men (15.7%) who either had depressive symptoms (PHQ-9≥10) or reported antidepressant use, of whom 54.1% (n=46) were receiving antidepressant medication.

Association of socio-economic and psychosocial (drinking and drug use) factors with depressive symptoms (PHQ-9≥10) at baseline

Younger age, unemployment, methamphetamine use in the past three months, and completion of baseline questionnaire before the first quarter of 2013 was associated with higher prevalence of depressive symptoms in unadjusted and adjusted analysis (see Tables 3 and 4).

For peer review only - http://bmjopen.bmj.com/site/about/guidelines.xhtml

Association of psychosocial factors (sexualized drug use, age at sex debut, IPV, internalized homophobia, 'outness') with depressive symptoms at the 12- and 24-month questionnaire

The prevalence of depressive symptoms was two to three-fold higher among men who reported: ever having been a victim of IPV, IPV victimization in the past year, ever having perpetrated IPV, perpetration of IPV in the past year, internalized homophobia, and concealment of sexual identity, in unadjusted and adjusted analysis (see Table 5).

Exploration of changes in depression prevalence

The baseline prevalence of suicidal ideation on PHQ-9 was significantly higher for men who completed their questionnaire before April 1st 2013 (n=71) compared to men whose completion was on or after April 1st 2013, from which time they were asked to disclose their response (n=469) (19.7% vs. 9.6%; Pearson χ^2 test p=0.011). Similarly, the baseline prevalence of depressive symptoms (PHQ-9≥10) was significantly higher among men who completed their questionnaire before versus on/after April 1st 2013 (21.1% vs. 7.3%; Pearson χ^2 test p<0.001). A possible calendar time effect can also be seen in Table 3, and this relationship remained after adjusting for socio-demographics. There was evidence of an increase in depression from baseline to month-12 for the 353 men with baseline completion on/after April 1st 2013 and 12-month data (7.9% vs. 14.7%), but not for the 57 men with baseline completion before April 1st 2013 and 12-month data (17.5% vs. 12.3%; McNemar's χ^2 test p-values 0.366 and <0.001 respectively). When investigating the modifying effect of baseline completion date on the association between follow-up time-point and depressive symptoms in GEEs, the interaction p-value was 0.004: for month-12 vs. baseline, PR 0.61 95% CI: 0.28, 1.34 for the time period before April 1st 2013 and PR 1.97 95% CI: 1.41, 2.75 for the time period on/after April 1st 2013 respectively.

Discussion

In the PROUD trial of GBMSM reporting very high levels of CAS partners, the prevalence of depressive symptoms by PHQ-9 ranged from 9.1% at baseline to 14.4% at months 12 and 24. Younger men, those who were not employed, those who reported methamphetamine use, IPV, internalized homophobia, and concealment of sexual identity, were more likely to report depressive symptoms. Depression prevalence did not differ significantly between trial arms at any time-point, which was in line with that reported in the iPrEX (placebo controlled) trial of PrEP [25].

For peer review only - http://bmjopen.bmj.com/site/about/guidelines.xhtml

The link between psychosocial factors, including depressive symptoms, and sexual risk behaviour among GBMSM in PROUD has been addressed in a separate paper [26]. Unlike in previous studies [16 17], no link was found between depression and CAS. Given the PROUD inclusion criteria, all participants reported recent CAS partners. It may be that symptoms of depression do not distinguish between men reporting higher levels of CAS partners and lower levels of CAS partners.

In the current study, findings suggest that some men may have underreported depressive symptoms at baseline after April 2013. It is possible that newly enrolled participants may have been worried about their inclusion in the trial if they disclosed thoughts of suicide or self-harm to the investigator, and that such concerns may have dissipated over time once participation in the study was established.

Patient and public involvement activities with a small group of PROUD participants (N=13), in the form of a short qualitative survey, provided some participant perspectives on the change in depressive symptoms over time. Some highlighted the possibility that greater familiarity with the questionnaire and study staff over time may have resulted in increased reporting of depressive symptoms at follow-up visits. Additionally, partaking in a clinical trial was thought to be one of the processes that helps individuals recognize/acknowledge their own depression in an environment where access to support is provided. Other suggested explanations for an actual increase in depressive symptoms included the possibility of greater socio-economic hardship in a period of national financial instability, risk taking behaviours in relation to CAS and chemsex, or PrEP-related stigma. None of the 13 participants specifically theorized that engagement in chemsex increased over the study, but chemsex was a common feature in discourse on depression. Although these participants were informed of the lack of difference between the immediate and deferred PrEP groups with regards to depression prevalence, some men cited that PrEP related side-effects and stigma, as well as fear of loss of access to PrEP after study completion, may have increased over time.

The depression prevalence observed in PROUD at month-12 (14.4%) and 24 (14.4%) is comparable to that found in the AURAH cross-sectional survey of HIV-negative GBMSM attending GUM services in England, 2013-2014 (PHQ-9≥10): 12.4% (n=166/1340) and 15.0% (n=122/815) among GBMSM reporting recent sex (with or without a condom) and recent CAS respectively [17]. Depression prevalence at annual follow-up visits in PROUD was also broadly similar to other studies of GBMSM in the UK [13-18], although comparison is made difficult due to different study settings and measures of

For peer review only - http://bmjopen.bmj.com/site/about/guidelines.xhtml

depression, and the fact that people with HIV were included in most studies (Textbox 1). The prevalence of depressive symptoms observed at follow-up in PROUD was almost twice that observed in a random sample of men and women in England (2007/2008); 7% reported PHQ-9≥10 [27], although socio-demographic differences may confound this comparison.

Although most studies based prevalence estimates on depressive symptoms only, the proportion of participants with evidence of current depression is higher when taking into account treatment for depression as well as symptoms. In the PROUD trial, 36 (78%) of the 46 men reporting antidepressant use at baseline were not positive for PHQ-9 depressive symptoms, and the prevalence of depression when considering symptoms or treatment was 15.7%.

In recent European studies, younger age, markers of lower socio-economic status, lower levels of a supportive network, reporting a bisexual identity [14 17 28], and recreational drug use [17 28], appear to be important factors for depression in the GBMSM literature. Mephedrone and crystal methamphetamine use have also been linked to acute symptoms of depression in a qualitative study of 30 gay men in London [9]. Some men reported a sense of loss of intimacy with a sexual partner when engaging in chemsex. There is also some evidence to suggest that group sex environments may leave some individuals vulnerable to mistreatment particularly if drugs are used, given their impact on inhibition and self-regulation [29]. Chemsex associated drug use has been found to be strongly associated with depressive symptoms among UK GBMSM in the AURAH study, including after adjustment for socio-demographic factors [17]. Although the pattern of associations was similar in the PROUD trial, some of the differences observed (i.e. between sexual identities, number of recreational drugs used, and any chemsex drug use) were not statistically significant. It may be that due to the possible underreporting of depression at baseline and the limited sample size of this trial, differences that may exist were not detected. It is also important to note that this sample of men is unique - they all reported very high levels of CAS partners and agreed to participate in an RCT. Therefore, associations may differ from other samples of GBMSM.

There is some evidence to suggest that CSA is associated with depression among GBMSM in the U.S. [30]. In the PROUD trial, a very young age at anal sex debut was not associated with depression. This measure may be a poor proxy for CSA as it is not likely to reflect or pick up all sexual abuse experienced. Strong associations were found of any IPV victimization and any IPV perpetration with depressive symptoms in the PROUD trial. Evidence from a recent meta-analysis of mostly U.S. studies suggests that exposure to any kind of IPV victimization is associated with increased odds of

For peer review only - http://bmjopen.bmj.com/site/about/guidelines.xhtml

depressive symptoms (pooled OR 1.52 95% CI: 1.24, 1.86) [8]. Similar findings were observed in six recent studies [5 18 31-34]. In one UK study (GUM clinic attendees, 2010-2011), depression (HADS≥8) was significantly elevated among men reporting IPV perpetration in the past year (20.7% vs. 11.5%), but the association was attenuated after adjusting for socio-economic factors (OR 3.7 95% CI: 1.0, 14.6; *p=0.060*) [18]. Adjusting for income may in part have contributed to the attenuation. Financial insecurity may be closely linked to depression, and financial inequalities within intimate partnerships may increase the risk of controlling and abusive behaviours. It was not possible to investigate the confounding effect of income/financial security in PROUD.

As described by syndemic theory, recreational drug use, IPV and depression may influence each other in a cyclical pattern, with implications for an exaggerated risk of poor health outcomes [5]. IPV may distort one's perceived self-worth and capacity to influence important outcomes in one's life, which may lead to depressive symptoms [35], in turn depression may heighten one's vulnerability to dysfunctional relationship dynamics as adaptive coping mechanisms are distorted [36-38]. Recreational drug use may cause symptoms of depression, which in turn results in further use of drugs to induce a state of cognitive release and escape from symptoms.

In line with minority stress theory, a measure of internalized homophobia and concealment of sexual identity was strongly associated with depressive symptoms in the PROUD trial. Due to the significance of early life socialization experiences, the minority stress model suggests that residual internalized homophobia may be a permanent fixture throughout the life-course for a sexual minority individual. If individuals integrate a high degree of antigay attitudes into their perception of self, depression may follow [2]. This may in part be the result of constant preoccupation with, and cognitive effort involved in, hiding one's identity [39], which is a profoundly heavy burden to carry. Three factors are hypothesized to reverse the process of internalized homophobia and reduce the risk of mental health distress: (i) family support, (ii) personal resources including personality characteristics, and (iii) group resources/social structural factors including community solidarity and cohesiveness. Very few studies have collected this information. There is a need to better understand correlates of these protective factors, as this may be useful for informing interventions to reduce the burden of depression among sexual minority men.

Strengths and limitations

This is the first UK study to collect data on depression prevalence over time among GBMSM, and investigate associations with a range of psychosocial factors. A change in methodology part way

For peer review only - http://bmjopen.bmj.com/site/about/guidelines.xhtml

through baseline data collection may have affected the reporting of depressive symptoms, limiting the validity of findings at baseline. Given the relatively small sample size of the PROUD trial, the examination of factors associated with depression was based on cross-sectional analysis, utilising all available observations. Some participants were not included in the GEE analysis of psychosocial correlates due to missing questionnaires at months 12 and 24. However, men with and without depressive symptoms (PHQ-9≥10) at baseline had similar proportions completing the 12-month questionnaire (22.5% vs. 24.2%; χ^2 test p=0.780) and those with and without depressive symptoms at the 12-month questionnaire had similar proportions completing the 24-month questionnaire (27.1% vs. 24.8%; χ^2 test p=0.702). When stratifying by trial arm, there remained no difference between men with and without depressive symptoms in terms of proportion completing follow-up questionnaires. Even after including repeated observations in GEE models, the analysis may have lacked power to accurately detect the presence of some associations. Furthermore, the direction of any causality may be unclear for some associations. For this reason, as well as the possibility of unmeasured confounders, inferences regarding causal factors for depression are limited. Although chemsex drug use was investigated in this study, the cultural phenomenon of chemsex was not explicitly measured, as participants were not asked whether these drugs were taken to enhance sexual experiences.

There have been no longitudinal studies that are designed and powered to investigate depression among GBMSM. Future longitudinal analyses would be useful in investigating the suggested causal pathways in this paper. Such a study would require careful consideration in how to measure incident depression, as new symptoms may indicate first episode or recurrent depression. Therefore, capturing data on previous diagnoses and treatment is important. Following participants from a young age is likely to yield the most accurate data with regards to depression incidence. Finally, there is a need to examine associations with measures of family and community support, income, and abuse in childhood, as well as the role of these factors in relationships investigated in this paper.

Conclusions and implications

In the context of interviewer administered surveys or surveys in which interviewers/study staff are made aware of individual responses, participants may be reluctant to disclose information on depressive symptomatology. This may be particularly true in clinical trial settings that are subject to strict inclusion/exclusion criteria, as eligible participants may perceive the disclosure of current depressive symptoms as prohibiting study inclusion. Researchers should be aware of the potential limitations to reporting depression prevalence under these circumstances.

For peer review only - http://bmjopen.bmj.com/site/about/guidelines.xhtml

Our findings add to the growing body of research in high-income countries suggesting a high concomitant burden of sexual minority related stress, substance use, and IPV with depression among GBMSM. Since these psychosocial measures are relevant to GBMSM at high-risk of HIV and seeking PrEP, it is recommended that training on awareness and enquiry about psychosocial issues should be enhanced in sexual health services. This should include referral to substance use services and culturally appropriate structures set up to address IPV among same-sex male couples. Furthermore, findings suggest that new interventions addressing sexual minority related stresses are needed in order to foster greater community affiliation and support, and curtail stress. Intervening with sexual minority youths in schools and/or in LGBT+ youth groups may prevent the internalizing of homophobic attitudes and the onset of mental health problems. Although it is important to be cognizant of the unique forms of stress experienced by sexual minority men, findings from PROUD also emphasise the link between socio-economic disadvantage and poor mental health. Therefore, emphasis on alleviating economic hardship together with efforts to promote self-acceptance and pride towards one's sexual orientation is recommended when addressing depression among sexual minority men.

In many countries, sexual minorities are being accepted and celebrated more so than ever in history [40-43]. This social climate provides a solid platform from which to launch extensive efforts to eliminate homophobic discrimination in society at large. Such a structural change is likely to be profoundly beneficial to the health and wellbeing of sexual minority people. Efforts may include national educational campaigns and specialised training for health care providers on homophobia and heterosexism.

Ethics approval and consent to participate

The study was reviewed and approved by London Bridge Research Ethics Committee (12/LO/1289). Written informed consent was obtained from all participants. The study protocol is available online (http://www.proud.mrc.ac.uk/about/study-protocol/).

Data Sharing Statement

The PROUD data is held at MRC CTU at UCL, which encourages optimal use of data by employing a controlled access approach to data sharing, incorporating a transparent and robust system to review requests and provide secure data access consistent with the relevant ethics committee approvals. All requests for data are considered and can be initiated by contacting proud.mrcctu@ucl.ac.uk.

For peer review only - http://bmjopen.bmj.com/site/about/guidelines.xhtml

Competing interests

The PROUD study was provided drug free of charge by Gilead Sciences plc. which also distributed it to participating clinics and provided funds for additional diagnostic tests for HCV and drug levels. AP has received payments for presentations made at meetings sponsored by Gilead in spring 2015. EW has had tuition fees and a stipend paid by Gilead. AC received advisory board fees from Gilead Sciences plc. and GSK/ViiV; speaker fees from Gilead and conferences bursaries from Gilead & Janssen. SM reports grants from the European Union H2020 scheme, EDCTP 2, the National Institute of Health Research, and Gilead Sciences; other support from Gilead Sciences, and the Population Council Microbicide Advisory Board; and is Chair of the Project Advisory Committee for USAID grant awarded to CONRAD to develop tenofovir-based products for use by women (non-financial).

Authors' contributions

Conceived and designed the study: MG FCL ARM SM DD EW AR AP LS AC AKS IR. Analysed the data: ARM. Wrote the paper: ARM MG FCL. All authors read and approved the final manuscript.

Funding

Sheena McCormack and David Dunn were supported by a Medical Research Council grant (MRC_UU_12023/23).

Acknowledgements

We thank all PROUD trial participants.

The PROUD study group:

David I. Dolling, Monica Desai, Alan McOwan, Richard Gilson, Amanda Clarke, Martin Fisher, Gabriel Schembri, Ann K. Sullivan, Nicola Mackie, Iain Reeves, Mags Portman, Vanessa Apea, John Saunders, Julie Fox, Jake Bayley, Michael Brady, Killian Quinn, Christine Bowman, Clarie Dewsnap, Charles J. Lacey, Stephen Taylor, David White, Simone Antonucci, Mitzy Gafos, Sheena McCormack, Owen N. Gill, David T. Dunn, and Anthony Nardone.

PROUD clinic teams:

Drew Clark, Paul Davis, James Hand, Machel Hunt, Rebecca Neale, Jackie O'Connell, Liat Sarner, John Saunders, Louise Terry, Angelina Twumasi, Salina Tsui, Dayan Vijeratnam, Ryan Whyte, Andy Williams, Sian Gately, Gerry Gilleran, Jill Lyons, Chris McCormack, Katy Moore, Cathy Stretton, Alex

For peer review only - http://bmjopen.bmj.com/site/about/guidelines.xhtml

For peer review only

Acheampong, Michael Bramley, Marion Campbell, Ruby Chowdhry, Stewart Eastwood, Babs Fennell, Wendy Hadley, Kerry Hobbs, Sarah Kirk, Nicky Perry, Charlotte Rawlinson, Celia Richardson, Claire Richardson, Mark Roche, Emma Simpkin, Simon Shaw, Elisa Souto, Julia Williams, Elaney Youssef, Tristan Barber, Cindy Eliot, Serge Fedele, Chris Higgs, Kathryn McCormick, Alexandra Meijer, Sam Pepper, Jane Rowlands, Gurmit Singh, Alfredo SolerCarracedo, Sonali Sonecha, David Taylor, Lervina Thomas, Frederick Attakora, Marina Bourke, Richard Castles, Rebecca Clark, Anke De-Masi, Veronica Espa, Rumbidzai Hungwe, Martin Lincoln, Sifiso Mguni, Rhianon Nevin-Dolan, Hannah Alexander, Lucy Campbell, Sophie Candfield, Shema Doshi, Olivia Liddle, Larissa Mulka, Priyanka Saigal, James Stevenson, James Boateng, Brynn Chappell, Susanna Currie, Carolyn Davies, Dornubari Lebari, Matthew Phillips, Lisa Southon, Sarah Thorpe, Anna Vas, Chris Ward, Claire Warren, Stephanie Yau , Alejandro Arenas-Pinto, Asma Ashraf, Matthew Bolton, Lewis Haddow, Sara McNamara, Ana Milinkovic, June Minton, Dianne Morris, Clare Oakland, Steve O'Farrell, Pierre Pellegrino, Sarah Pett, Nina Vora, Carmel Young, Taris Zarko-Flynn, Wilbert Ayap, Ling Jun Chen, Adam Croucher, Sarah Fidler, Kristin Kuldanek, Ken Legg, Agathe Leon, Nadia Naous, Severine Rey, Judith Zhou, Margaret-Anne Bevan, Nina Francia, Eleanor Hamlyn, Lisa Hurley, Helen Iveson, Isabelle Jendrulek, Tammy Murray, Alice Sharp, Andrew Skingsley, Chi Kai Tam, Al Teague, Caroline Thomas, Juan-Manuel Tiraboschi, Christine Brewer, Richard Evans, Jan Gravely, Gary Lamont, Fabiola Martin, Georgina Morris, Sarah Russell-Sharpe, John Wightman, Anthony Bains, Gill Bell, Terry Cox, Charlie Hughes, Hannah Loftus, Naomi Sutton, Debbie Talbot, Vince Tucker.

For peer review only

For peer review only - http://bmjopen.bmj.com/site/about/guidelines.xhtml

Table 1: Prevalence of demographic, socio-economic, and psychosocial (drinking and drug use) factors at baseline in PROUD

Measure:	Category:	Baseline [N=540] n (%)
Calendar time (year & calendar quarter [Q]) of baseline data collection	2012 Q4<br>2013 Q1<br>2013 Q2<br>2013 Q3<br>2013 Q4<br>2014 Q1<br>2014 Q2	8 (1.5%)<br>63 (11.7%)<br>85 (15.7%)<br>113 (20.9%)<br>119 (22.0%)<br>125 (23.2%)<br>27 (5.0%)
Age	<25<br>25-29<br>30-34<br>35-39<br>40-44<br>45+	54 (10.0%)<br>96 (17.8%)<br>114 (21.1%)<br>97 (18.0%)<br>81 (15.0%)<br>98 (18.2%)
Born in the UK and ethnicity	Yes, white<br>Yes, BAME<br>No, white<br>No, BAME	287 (53.4%)<br>35 (6.5%)<br>152 (28.3%)<br>64 (11.9%)
Self-reported sexual identity	Gay<br>Bisexual<br>Straight	513 (95.7%)<br>17 (3.2%)<br>6 (1.1%)
University Education	Yes<br>No/missing	327 (60.6%)<br>213 (39.4%)
Employed	Yes<br>No/missing	439 (81.3%)<br>101 (18.7%)
Ongoing relationship	Yes<br>No/missing	246 (45.6%)<br>294 (54.4%)
Study region	London<br>Outside London	375 (69.4%)<br>165 (30.6%)
Higher-risk drinking	No/missing<br>Yes	367 (68.0%)<br>173 (32.0%)
Recreational drug use (past 3 months)	0/missing<br>1<br>2-4<br>5+	148 (27.4%)<br>87 (16.1%)<br>159 (29.4%)<br>146 (27.0%)
Chemsex-associated drug use (past 3 months)	No/missing<br>Yes	309 (57.2%)<br>231 (42.8%)
Crystal meth (past 3 months)	No/missing<br>Yes	442 (81.9%)<br>98 (18.2%)
Mephedrone (past 3 months)	No/missing<br>Yes	343 (63.5%)<br>197 (36.5%)
GHB/GBL (past 3 months)	No/missing<br>Yes	371 (68.7%)<br>169 (31.3%)
Ketamine (past 3 months)	No/missing<br>Yes	451 (83.5%)<br>89 (16.5%)
Cocaine (past 3 months)	No/missing<br>Yes	401 (74.3%)<br>139 (25.7%)
Ecstasy/MDMA (past 3 months)	No/missing<br>Yes	450 (83.3%)<br>90 (16.7%)

For peer review only - http://bmjopen.bmj.com/site/about/guidelines.xhtml

Table 2: Prevalence of psychosocial factors (sexualized drug use, age at sex debut, IPV, internalized homophobia, ‘outness’) at follow-up time-points in PROUD

Measure:	Category:	12-month [N=410] n (%)	24-month [N=333] n (%)
Had sex after using recreational drugs (past 3 months)	No/missing<br>Yes	186 (45.4%)<br>224 (54.6%)	174 (52.3%)<br>159 (47.8%)
Age≤15 years at anal sex debut	No<br>Yes	307 (77.7%)<br>88 (22.3%)	251 (78.2%)<br>70 (21.8%)
Age<13 years at anal sex debut	No<br>Yes	373 (94.4%)<br>22 (5.6%)	302 (94.1%)<br>19 (5.9%)
Any IPV victimization	No/missing<br>Yes	226 (55.1%)<br>184 (44.9%)	199 (59.8%)<br>134 (40.2%)
Any IPV victimization in the past year	No/missing<br>Yes	346 (84.4%)<br>64 (15.6%)	284 (85.3%)<br>49 (14.7%)
Any IPV perpetration	No/missing<br>Yes	330 (80.5%)<br>80 (19.5%)	273 (82.0%)<br>60 (18.0%)
Any IPV perpetration in the past year	No/missing<br>Yes	378 (92.2%)<br>32 (7.8%)	310 (93.1%)<br>23 (6.9%)
Internalised homophobia	No/missing<br>Yes	239 (58.3%)<br>171 (41.7%)	195 (58.6%)<br>138 (41.4%)
Concealment of sexual identity	No<br>Yes	206 (51.6%)<br>193 (48.4%)	166 (50.6%)<br>162 (49.4%)

For peer review only

For peer review only - http://bmjopen.bmj.com/site/about/guidelines.xhtml

Table 3: Unadjusted and adjusted associations of demographic and socio-demographic factors with depressive symptoms at baseline in PROUD

N=540 men at baseline		Depressive symptoms (PHQ-9≥10)					
		%	*p-value*[a]	Unadjusted PR [95% CI]	Overall *p-value*[d]	Adjusted[e] PR [95% CI]	*Overall p-value*[d]
Calendar time (year & calendar quarter [Q])[f]	2012 Q4 – 2013 Q1	21.1%	*0.001*	1	*0.003*	1	*0.004*
	2013 Q2	2.4%	*0.20*[b]	0.11 [0.03, 0.47]	*0.25*[b]	0.12 [0.03, 0.47]	*0.26*[b]
	2013 Q3	7.1%		0.34 [0.15, 0.75]		0.34 [0.15, 0.76]	
	2013 Q4	8.4%		0.40 [1.19, 0.84]		0.39 [0.18, 0.83]	
	2014 Q1 – Q2	9.2%		0.44 [0.22, 0.85]		0.45 [0.23, 0.88]	
Age	<25	22.2%	*0.003*	3.63 [1.44, 9.13]	*0.006*	3.49 [1.44, 8.44]	*0.003*
	25-29	8.3%	*0.002*[b]	1.36 [0.49, 3.78]	*0.003*[b]	1.22 [0.43, 3.51]	*0.004*[b]
	30-34	12.3%		2.01 [0.80, 5.02]		1.97 [0.78, 4.97]	
	35-39	6.2%		1.01 [0.34, 3.03]		0.96 [0.31, 3.00]	
	40-44	3.7%		0.60 [0.16, 2.35]		0.60 [0.16, 2.31]	
	45+	6.1%		1		1	
Born in the UK and white ethnicity	Yes, white	9.1%	*0.32*[c]	1	*0.37*	1	*0.44*
	Yes, BAME	14.3%		1.58 [0.65, 3.84]		0.93 [0.41, 2.09]	
	No, white	6.6%		0.73 [0.36, 1.47]		0.77 [0.36, 1.65]	
	No, BAME	12.5%		1.38 [0.65, 2.91]		1.59 [0.72, 3.54]	
Self-reported sexual identity[g]	Gay	9.0%	*0.46*[c]	1	*0.50*	1	*0.64*
	Bisexual	13.0%		1.45 [0.49, 4.33]		1.26 [0.48, 3.31]	
	Straight						
University Education	Yes	8.9%	*0.84*	1	*0.84*	1	*0.94*
	No/missing	9.4%		1.06 [0.61, 1.82]		0.98 [0.54, 1.77]	
Employed	Yes	6.8%	*<0.001*	1	*<0.001*	1	*0.001*
	No/missing	18.8%		2.75 [1.62, 4.69]		2.52 [1.45, 4.37]	
Ongoing relationship	Yes	6.5%	*0.06*	1	*0.06*	1	*0.15*
	No/missing	11.2%		1.73 [0.97, 3.06]		1.50 [0.86, 2.63]	
Study region	London	9.3%	*0.75*	1	*0.75*	1	*0.66*
	Outside London	8.5%		0.91 [0.50, 1.64]		0.86 [0.44, 1.69]	

BAME= Black, Asian and minority ethnic.

[a] Pearson χ^2 test.

[b] Test for trend.

[c] Fisher's exact test.

[d] p-value by Wald test using modified Poisson regression with a robust variance estimator in order to produce adjusted prevalence ratios.

For peer review only - http://bmjopen.bmj.com/site/about/guidelines.xhtml

[e] Age (included as four categories: <25, 25-29, 30-39, 40+), born in the UK, sexual identity (gay or bisexual/straight), university education, relationship status, London study clinic site, and calendar time.
[f] Some calendar quarters (Q4 2012 & Q1 2013; Q1-Q2 2014) were combined due to small cell counts.
[g] A dichotomized version of self-reported sexual identity is investigated in analysis due to very small numbers: gay or bisexual/straight.

For peer review only

Table 4: Unadjusted and adjusted associations of psychosocial factors (drinking and drug use) with depressive symptoms at baseline in PROUD

N=540 men at baseline		Depressive symptoms (PHQ-9≥10)					
		%	*p-value*[a]	Unadjusted PR [95% CI]	Overall *p-value*[d]	Adjusted[e] PR [95% CI]	Overall *p-value*[d]
Higher-risk drinking	No/missing<br>Yes	8.5%<br>10.4%	*0.46*	1<br>1.23 [0.71, 2.14]	*0.46*	1<br>1.25 [0.68, 2.29]	*0.48*
Recreational drug use (past 3 months)	0/missing<br>1<br>2-4<br>5+	10.1%<br>4.6%<br>6.9%<br>13.0%	*0.11*<br>*0.40*[b]	1<br>0.45 [0.16, 1.32]<br>0.68 [0.32, 1.44]<br>1.28 [0.68, 2.43]	0.13<br>*0.45*[b]	1<br>0.57 [0.20, 1.64]<br>0.89 [0.39, 2.02]<br>1.34 [0.71, 2.56]	0.32<br>*0.32*[b]
Chemsex-associated drug use	No/missing<br>Yes	8.1%<br>10.4%	*0.36*	1<br>1.28 [0.75, 2.19]	*0.36*	1<br>1.31 [0.77, 2.23]	*0.33*
Crystal meth (past 3 months)	No/missing<br>Yes	7.7%<br>15.3%	*0.02*	1<br>1.99 [1.13, 3.51]	*0.02*	1<br>1.95 [1.06, 3.61]	*0.03*
Mephedrone (past 3 months)	No/missing<br>Yes	9.0%<br>9.1%	*0.97*	1<br>1.01 [0.58, 1.76]	*0.97*	1<br>0.99 [0.56, 1.73]	*0.96*
GHB/GBL (past 3 months)	No/missing<br>Yes	8.4%<br>10.7%	*0.39*	1<br>1.27 [0.73, 2.21]	*0.39*	1<br>1.23 [0.72, 2.11]	*0.46*
Ketamine (past 3 months)	No/missing<br>Yes	8.4%<br>12.4%	*0.24*	1<br>1.47 [0.78, 2.76]	*0.24*	1<br>1.43 [0.76, 2.71]	*0.27*
Cocaine (past 3 months)	No/missing<br>Yes	8.5%<br>10.8%	*0.41*	1<br>1.27 [0.72, 2.27]	*0.41*	1<br>1.14 [0.66, 1.97]	*0.64*
Ecstasy/MDMA (past 3 months)	No/missing<br>Yes	8.4%<br>12.2%	*0.26*	1<br>1.45 [0.77, 2.72]	*0.25*	1<br>1.31 [0.69, 2.49]	*0.41*

[a] Pearson χ^2 test.
[b] Test for trend.
[c] Fisher's exact test.
[d] p-value by Wald test using modified Poisson regression with a robust variance estimator in order to produce adjusted prevalence ratios.
[e] Age (included as four categories: <25, 25-29, 30-39, 40+), born in the UK, sexual identity (gay or bisexual/straight), university education, relationship status, London study clinic site, and calendar time.

For peer review only - http://bmjopen.bmj.com/site/about/guidelines.xhtml

Table 5: Unadjusted and adjusted associations of psychosocial factors (sexualized drug use, age at sex debut, IPV, internalized homophobia, 'outness') with depressive symptoms using 12- and 24-month PROUD questionnaire data in GEE models

N=436 men, observations=743 (using data from men who completed the 12-month or 24-month questionnaire in GEE models)		Depressive symptoms (PHQ-9≥10)			
		Unadjusted PR [95% CI]	Overall p-value[a]	Adjusted[b] PR [95% CI]	Overall p-value[a]
Had sex after using recreational drugs (past 3 months)<br>N=436[c], Obs=743[d] ; N=432[e], Obs=737[f]	No/missing<br>Yes	1<br>1.38 [0.93, 2.06]	*0.11*	1<br>1.38 [0.92, 2.07]	*0.12*
Age≤15 years at anal sex debut<br>N=433[c], Obs=716[d] ; N=429[e], Obs=710[f]	No<br>Yes	1<br>1.27 [0.80, 2.02]	*0.31*	1<br>1.20 [0.75, 1.94]	*0.45*
Age<13 years at anal sex debut<br>N=433[c], Obs=716[d] ; N=429[e], Obs=710[f]	No<br>Yes	1<br>0.71 [0.26, 1.94]	0.51	1<br>0.72 [0.27, 1.97]	*0.526*
Any IPV victimization<br>N=436[c], Obs=743[d] ; N=432[e], Obs=737[f]	No/missing<br>Yes	1<br>2.45 [1.63, 3.67]	*<0.001*	1<br>2.56 [1.70, 3.85]	*<0.001*
Any IPV victimization in the past year<br>N=436[c], Obs=743[d] ; N=432[e], Obs=737[f]	No/missing<br>Yes	1<br>2.82 [1.88, 4.22]	*<0.001*	1<br>2.96 [1.97, 4.46]	*<0.001*
Any IPV perpetration<br>N=436[c], Obs=743[d] ; N=432[e], Obs=737[f]	No/missing<br>Yes	1<br>2.83 [1.89, 4.22]	*<0.001*	1<br>2.86 [1.90, 4.30]	*<0.001*
Any IPV perpetration in the past year<br>N=436[c], Obs=743[d] ; N=432[e], Obs=737[f]	No/missing<br>Yes	1<br>3.40 [2.13, 5.41]	*<0.001*	1<br>3.36 [2.06, 5.47]	*<0.001*
Internalised homophobia<br>N=436[c], Obs=743[d]; N=432[e], Obs=737[f]	No/missing<br>Yes	1<br>1.92 [1.30, 2.83]	*0.001*	1<br>1.91 [1.28, 2.83]	*0.001*
Concealment of sexual identity<br>N=434[c], Obs=727[d]; N=431[e], Obs=722[f]	No<br>Yes	1<br>1.75 [1.17, 2.62]	*0.007*	1<br>1.71 [1.13, 2.59]	*0.012*

[a] p-value by Wald test using GEE models.
[b] Age (included as four categories: <25, 25-29, 30-39, 40+), born in the UK, sexual identity (gay or bisexual/straight), university education, London study clinic site, calendar time (year & calendar quarter), and follow-up time-point (month-12, month-24).
[c] Number of men contributing observations to the unadjusted model.
[d] Number of observations examined in the unadjusted model.
[e] Number of men contributing observations to the adjusted model.
[f] Number of observations examined in the adjusted model.

For peer review only

Textbox 1: Prevalence of depression on symptom questionnaires in studies of UK GBMSM

Study	Recruitment dates	Sampling strategy	Size of GBMSM sample	Measure of depression	Depression prevalence
Cross-sectional survey of GBMSM in London[16]	1999	Consecutive sampling of GBMSM attending a GUM clinic	122	HADS score of ≥8	18.8%
Cross-sectional survey of GBMSM in London[18]	2010-2011	Consecutive sampling of GBMSM attending GUM clinics	519	HADS score of ≥8	12%
Natsal-3[13]	2010-2012	Multistage probability sampling of the general British population	190	PHQ-2 score of ≥3	8.9%
Cross-sectional survey of GBMSM in London[15]	2016	Volunteer sampling of users of a geosocial-networking app for GBMSM	179	PHQ-2 score of ≥3	22.3%
Gay & Bisexual Men's Health Survey[14]	2011	Snowball sampling of GBMSM	5416	PHQ-9 score of ≥10	21.3%
AURAH[17]	2013-2014	Consecutive sampling of HIV-negative GBMSM attending GUM clinics in England	1340 sexually active GBMSM	PHQ-9 score of ≥10	12.4%
PROUD	2012-2014 baseline<br>2013-2015 month-12<br>2014-2016 month-24	Volunteer sampling of HIV-negative GBMSM attending GUM clinics and community organizations, and GBMSM users of social media	540<br>410<br>333	PHQ-9 score of ≥10	9.1%<br>14.4%<br>14.4%

For peer review only - http://bmjopen.bmj.com/site/about/guidelines.xhtml

References:

1. King M, Semlyen J, Tai SS, et al. A systematic review of mental disorder, suicide, and deliberate self harm in lesbian, gay and bisexual people. *BMC Psychiatry* 2008;8(70) doi: 10.1186/1471-244X-8-70
2. Meyer IH. Prejudice, social stress, and mental health in lesbian, gay, and bisexual populations: conceptual issues and research evidence. *Psychol Bull* 2003;129(5):674-97. doi: 10.1037/0033-2909.129.5.674 [published Online First: 2003/09/06]
3. Klein H. Depression and HIV Risk Taking among Men Who Have Sex with Other Men (MSM) and Who Use the Internet to Find Partners for Unprotected Sex. *J Gay Lesbian Ment Health* 2014;18(2):164-89. doi: 10.1080/19359705.2013.834858 [published Online First: 2014/01/01]
4. Perdue TE, Hagan H, Thiede H, et al. Depression and HIV risk behaviour among Seattle-area injection drug users and young men who have sex with men. *AIDS Educ Prev* 2003;15(1):81-92.
5. Mimiaga MJ, Biello KB, Robertson AM, et al. High prevalence of multiple syndemic conditions associated with sexual risk behavior and HIV infection among a large sample of Spanish- and Portuguese-speaking men who have sex with men in Latin America. *Arch Sex Behav* 2015;44(7):1869-78. doi: 10.1007/s10508-015-0488-2 [published Online First: 2015/07/15]
6. Cochran SD, Mays VM. Burden of psychiatric morbidity among lesbian, gay, and bisexual individuals in the California Quality of Life Survey. *J Abnorm Psychol* 2009;118(3):647-58. doi: 10.1037/a0016501 [published Online First: 2009/08/19]
7. Stall R, Mills TC, Williamson J, et al. Association of co-occurring psychosocial health problems and increased vulnerability to HIV/AIDS among urban men who have sex with men. *Am J Public Health* 2003;93(6):939-42. [published Online First: 2003/05/30]
8. Buller AM, Devries KM, Howard LM, et al. Associations between intimate partner violence and health among men who have sex with men: a systematic review and meta-analysis. *PLoS Med* 2014;11(3):e1001609. doi: 10.1371/journal.pmed.1001609 [published Online First: 2014/03/07]
9. Bourne A, Reid D, Hickson F, et al. "Chemsex" and harm reduction need among gay men in South London. *Int J Drug Policy* 2015;26(12):1171-6. doi: 10.1016/j.drugpo.2015.07.013 [published Online First: 2015/08/25]
10. Sewell J, Miltz A, Lampe FC, et al. Poly drug use, chemsex drug use, and associations with sexual risk behaviour in HIV-negative men who have sex with men attending sexual health clinics. *Int J Drug Policy* 2017;43:33-43. doi: 10.1016/j.drugpo.2017.01.001 [published Online First: 2017/02/13]
11. Pufall EL, Kall M, Shahmanesh M, et al. Sexualized drug use ('chemsex') and high-risk sexual behaviours in HIV-positive men who have sex with men. *HIV Med* 2018;19(4):261-70. doi: 10.1111/hiv.12574 [published Online First: 2018/01/26]
12. Bourne A, Reid D, Hickson F, et al. The Chemsex Study: drug use in sexual settings among gay and bisexual men in Lambeth, Southwark & Lewisham: Sigma Research, 2014.
13. Mercer CH, Prah P, Field N, et al. The health and well-being of men who have sex with men (MSM) in Britain: Evidence from the third National Survey of Sexual Attitudes and Lifestyles (Natsal-3). *BMC Public Health* 2016;16(525) doi: 10.1186/s12889-016-3149-z
14. Hickson F, Davey C, Reid D, et al. Mental health inequalities among gay and bisexual men in England, Scotland and Wales: a large community-based cross-sectional survey. *J Public Health (Oxf)* 2017;39(2):266-73. doi: doi:10.1093/pubmed/fdw021
15. Goedel WC, Hagen D, Halkitis PN, et al. Post-exposure prophylaxis awareness and use among men who have sex with men in London who use geosocial-networking smartphone applications. *AIDS Care* 2017;29(5):579-86. doi: 10.1080/09540121.2016.1259455 [published Online First: 2016/12/03]
16. Beck A, McNally I, Petrak J. Psychosocial predictors of HIV/STI risk behaviours in a sample of homosexual men. *Sex Transm Infect* 2003;79(2):142-6. [published Online First: 2003/04/12]
17. Miltz AR, Rodger AJ, Sewell J, et al. Clinically significant depressive symptoms and sexual behaviour among men who have sex with men. *The British Journal of Psychiatry Open* 2017;3(3):127-37. doi: 10.1192/bjpo.bp.116.003574 [published Online First: 2017/05/17]
18. Bacchus LJ, Buller AM, Ferrari G, et al. Occurrence and impact of domestic violence and abuse in gay and bisexual men: A cross sectional survey. *Int J STD AIDS* 2017;28(1):16-27. doi: 10.1177/0956462415622886 [published Online First: 2016/01/09]
19. McCormack S, Dunn DT, Desai M, et al. Pre-exposure prophylaxis to prevent the acquisition of HIV-1 infection (PROUD): effectiveness results from the pilot phase of a pragmatic open-label randomised

For peer review only

trial. *Lancet* 2016;387(10013):53-60. doi: 10.1016/s0140-6736(15)00056-2 [published Online First: 2015/09/14]

20. Kroenke K, Spitzer RL, Williams JB. The PHQ-9: validity of a brief depression severity measure. *J Gen Intern Med* 2001;16(9):606-13. [published Online First: 2001/09/15]
21. Babor TF, Higgins-Biddle JC, Saunders JB, et al. The Alcohol Use Disorders Identification Test. Guidelines for Use in Primary Care: Department of Mental Health and Substance Dependence, World Health Organization, 2001.
22. Ross MW, Rosser BR. Measurement and correlates of internalized homophobia: a factor analytic study. *J Clin Psychol* 1996;52(1):15-21. doi: 10.1002/(sici)1097-4679(199601)52:1<15::aid-jclp2>3.0.co;2-v [published Online First: 1996/01/01]
23. Zou G. A modified poisson regression approach to prospective studies with binary data. *Am J Epidemiol* 2004;159(7):702-6. [published Online First: 2004/03/23]
24. STATA statistical software. 13 ed [program]. Texas, 2009.
25. Defechereux PA, Mehrotra M, Liu AY, et al. Depression and Oral FTC/TDF Pre-exposure Prophylaxis (PrEP) Among Men and Transgender Women Who Have Sex With Men (MSM/TGW). *AIDS Behav* 2016;20(7):1478-88. doi: 10.1007/s10461-015-1082-2 [published Online First: 2015/06/17]
26. Miltz AR, Lampe FC, Bacchus LJ, et al. Intimate partner violence, depression, and sexual behaviour among gay, bisexual and other men who have sex with men in the PROUD trial. *BMC Public Health* 2019;19(1):431. doi: 10.1186/s12889-019-6757-6 [published Online First: 2019/04/27]
27. Paranjothy S, Gallacher J, Amlot R, et al. Psychosocial impact of the summer 2007 floods in England. *BMC Public Health* 2011;11:145. doi: 10.1186/1471-2458-11-145 [published Online First: 2011/03/05]
28. Vanden Berghe W, Nostlinger C, Laga M. Syndemic and other risk factors for unprotected anal intercourse among an online sample of Belgian HIV negative men who have sex with men. *AIDS Behav* 2014;18(1):50-8. doi: 10.1007/s10461-013-0516-y [published Online First: 2013/05/18]
29. Weatherburn P, Hickson F, Reid D, et al. Motivations and values associated with combining sex and illicit drugs ('chemsex') among gay men in South London: findings from a qualitative study. *Sex Transm Infect* 2017;93:153-54.
30. Mimiaga MJ, Noonan E, Donnell D, et al. Childhood sexual abuse is highly associated with HIV risk-taking behavior and infection among MSM in the EXPLORE Study. *J Acquir Immune Defic Syndr* 2009;51(3):340-8. doi: 10.1097/QAI.0b013e3181a24b38 [published Online First: 2009/04/16]
31. Brown MJ, Serovich JM, Kimberly JA. Depressive Symptoms, Substance Use and Partner Violence Victimization Associated with HIV Disclosure Among Men Who have Sex with Men. *AIDS Behav* 2016;20(1):184-92. doi: 10.1007/s10461-015-1122-y [published Online First: 2015/07/01]
32. Wang HY, Wang N, Chu ZX, et al. Intimate Partner Violence Correlates With A Higher HIV Incidence Among MSM: A 12-Month Prospective Cohort Study in Shenyang, China. *Sci Rep* 2018;8(1):2879. doi: 10.1038/s41598-018-21149-8 [published Online First: 2018/02/15]
33. De Boni RB, Machado IK, De Vasconcellos MTL, et al. Syndemics among individuals enrolled in the PrEP Brasil Study. *Drug and alcohol dependence* 2018;185:168-72. doi: 10.1016/j.drugalcdep.2017.12.016 [published Online First: 2018/02/20]
34. Jiang H, Chen X, Li J, et al. Predictors of condom use behavior among men who have sex with men in China using a modified information-motivation-behavioral skills (IMB) model. *BMC Public Health* 2019;19(1):261. doi: 10.1186/s12889-019-6593-8 [published Online First: 2019/03/06]
35. Fife RS, Schrager SS. Family violence. What health care providers need to know. Sudbury, MA: Jones & Bartlett Learning, LLC 2012.
36. Beck AT. The evolution of the cognitive model of depression and its neurobiological correlates. *Am J Psychiatry* 2008;165(8):969-77. doi: 10.1176/appi.ajp.2008.08050721 [published Online First: 2008/07/17]
37. Herek GM, Sims C, editors. *Sexual orientation and violent victimization: hate crimes and intimate partner violence among gay and bisexual males in the United States*. New York: Oxford University Press, 2008.
38. Goldenberg T, Stephenson R, Freeland R, et al. "Struggling to be the alpha": Sources of tension and intimate partner violence in same-sex relationships between men. *Culture, Health & Sexuality* 2016;18:875-89.
39. Operario D, Fiske ST. Ethnic identity moderates perceptions of prejudice: judgments of personal versus group discrimination and subtle versus blatant bias. *Personality and Social Psychology Bulleting* 2001;27:550-61.
40. Equality Act 2010. 2010 CHAPTER 15 In: Parliament U, ed.
41. Marriage (Same Sex Couples) Act 2013. 2013 CHAPTER 30. In: Parliament U, ed.

For peer review only - http://bmjopen.bmj.com/site/about/guidelines.xhtml

42. Gyimah S. Thousands officially pardoned under 'Turing's Law': Ministry of Justice, 2017.
43. BBC. Same-sex marriage now legal in Northern Ireland: BBC newsround; 2019 [Available from: https://www.bbc.co.uk/newsround/50135994 accessed October 22 2019.

For peer review only

For peer review only - http://bmjopen.bmj.com/site/about/guidelines.xhtml

STROBE Statement—Checklist of items that should be included in reports of ***cross-sectional studies***

	Item No	Recommendation	Page No
**Title and abstract**	1	(*a*) Indicate the study's design with a commonly used term in the title or the abstract	1-2
		(*b*) Provide in the abstract an informative and balanced summary of what was done and what was found	2
**Introduction**			
Background/rationale	2	Explain the scientific background and rationale for the investigation being reported	3-4
Objectives	3	State specific objectives, including any prespecified hypotheses	4
**Methods**			
Study design	4	Present key elements of study design early in the paper	5-8
Setting	5	Describe the setting, locations, and relevant dates, including periods of recruitment, exposure, follow-up, and data collection	5
Participants	6	(*a*) Give the eligibility criteria, and the sources and methods of selection of participants	5
Variables	7	Clearly define all outcomes, exposures, predictors, potential confounders, and effect modifiers. Give diagnostic criteria, if applicable	5-7
Data sources/ measurement	8*	For each variable of interest, give sources of data and details of methods of assessment (measurement). Describe comparability of assessment methods if there is more than one group	5-7
Bias	9	Describe any efforts to address potential sources of bias	7-8
Study size	10	Explain how the study size was arrived at	5
Quantitative variables	11	Explain how quantitative variables were handled in the analyses. If applicable, describe which groupings were chosen and why	7-8
Statistical methods	12	(*a*) Describe all statistical methods, including those used to control for confounding	7-8
		(*b*) Describe any methods used to examine subgroups and interactions	/
		(*c*) Explain how missing data were addressed	5-8
		(*d*) If applicable, describe analytical methods taking account of sampling strategy	7-8
		(*e*) Describe any sensitivity analyses	8
**Results**			
Participants	13*	(a) Report numbers of individuals at each stage of study—eg numbers potentially eligible, examined for eligibility, confirmed eligible, included in the study, completing follow-up, and analysed	8-9
		(b) Give reasons for non-participation at each stage	8-9
		(c) Consider use of a flow diagram	/
Descriptive data	14*	(a) Give characteristics of study participants (eg demographic, clinical, social) and information on exposures and potential confounders	8-9
		(b) Indicate number of participants with missing data for each variable of interest	8
Outcome data	15*	Report numbers of outcome events or summary measures	9
Main results	16	(*a*) Give unadjusted estimates and, if applicable, confounder-adjusted estimates and their precision (eg, 95% confidence interval). Make clear which confounders were adjusted for and why they were included	9-10

For peer review only

For peer review only - http://bmjopen.bmj.com/site/about/guidelines.xhtml

		(*b*) Report category boundaries when continuous variables were categorized	/
		(*c*) If relevant, consider translating estimates of relative risk into absolute risk for a meaningful time period	/
Other analyses	17	Report other analyses done—eg analyses of subgroups and interactions, and sensitivity analyses	8
**Discussion**			
Key results	18	Summarise key results with reference to study objectives	11
Limitations	19	Discuss limitations of the study, taking into account sources of potential bias or imprecision. Discuss both direction and magnitude of any potential bias	13
Interpretation	20	Give a cautious overall interpretation of results considering objectives, limitations, multiplicity of analyses, results from similar studies, and other relevant evidence	13
Generalisability	21	Discuss the generalisability (external validity) of the study results	12
**Other information**			
Funding	22	Give the source of funding and the role of the funders for the present study and, if applicable, for the original study on which the present article is based	14

*Give information separately for exposed and unexposed groups.

Note: An Explanation and Elaboration article discusses each checklist item and gives methodological background and published examples of transparent reporting. The STROBE checklist is best used in conjunction with this article (freely available on the Web sites of PLoS Medicine at http://www.plosmedicine.org/, Annals of Internal Medicine at http://www.annals.org/, and Epidemiology at http://www.epidem.com/). Information on the STROBE Initiative is available at www.strobe-statement.org.

For peer review only
